# Effectiveness of Nonpharmacological Interventions for Improving the Mental Health and Other Psychosocial Outcomes of Parents with Perinatal Loss: A Systematic Review and Meta-Analysis

**DOI:** 10.1155/2024/2181544

**Published:** 2024-08-28

**Authors:** Si Ni Li, Li Yang, Qing Yu Wang, Si Pan, Mei Hui Xiao, Jiao Xie

**Affiliations:** ^1^Clinical Nursing Teaching and Research Section, The Second Xiangya Hospital, Central South University, Changsha, China; ^2^The Nethersole School of Nursing, Faculty of Medicine, The Chinese University of Hong Kong, Ma Liu Shui, Hong Kong; ^3^Department of Metabolism and Endocrinology, The Second Xiangya Hospital, Central South University, Changsha, Hunan, China

## Abstract

The objective of this study was to assess the effectiveness of nonpharmacological interventions in enhancing the mental health and psychosocial outcomes of parents who have experienced perinatal loss, in comparison to active/inactive control groups. It also sought to identify the characteristics and components of effective interventions in the included studies. A comprehensive search was conducted across seven databases from January 2008 to December 2023. The analysis included randomized controlled trials (RCTs) published in English journals and dissertations that reported at least one mental health outcome postintervention for parents with perinatal loss. Twenty-one RCTs were included. The findings demonstrated significant improvements of nonpharmacological interventions in the grief, posttraumatic stress disorder, depression, anxiety, and perceived social support of parents with perinatal loss, with moderate-to-high certainty of evidence. Optimal characteristics of nonpharmacological interventions for parents with perinatal loss were identified to inform future studies, including the intervention approaches (cognitive-based interventions), delivery modalities (face-to-face), and formats (individual-based), and a recommended number of sessions of four or fewer. To validate these findings and explore the process of nonpharmacological interventions in improving health outcomes for parents with perinatal loss from diverse backgrounds, further rigorous RCTs with larger sample sizes and longer follow-up periods are required.

## 1. Introduction

Perinatal loss refers to the unintended or involuntary loss of a fetus or infant during pregnancy or shortly after birth. It encompasses various types of loss, including ectopic pregnancies, miscarriage (prior to 20 weeks of gestation), recurrent miscarriage (experiencing two or more consecutive miscarriages within 24 weeks of gestation), stillbirth (occurring after 20 weeks gestation with a birth weight of over 500 g), termination of pregnancy due to fetal anomaly (TOPFA), or neonatal loss (occurring within the first 28 days of life) [1]. In the United States, there are approximately 2.4 million fetal and neonatal deaths annually during the perinatal period—4 times higher than the number of cancer deaths [2]. In China, the stillbirth rate was 13.2 per 1,000 births in 2015–2016, with an estimated one-third of these potentially preventable [3]. Globally, there are over 6.3 million perinatal deaths reported each year [4], including approximately 2.6 million stillbirths [5]. Moreover, it is estimated that 15%–50% of all pregnancies end in miscarriage [6]. These statistics highlight the significant number of families who are affected by perinatal loss each year. Despite its prevalence, perinatal loss remains a topic that is often surrounded by silence and stigma, leading to a lack of awareness and support for affected families [7].

Perinatal loss is a complex and multifaceted experience that can have profound emotional, psychological, and social impacts on parents and their families. The loss of a child during the perinatal period is often unexpected and can elicit a range of intense emotions, including sadness, guilt, anger, and confusion [8]. These emotional difficulties can increase the risk of developing mental health disorders, such as grief, depression, anxiety, posttraumatic stress disorder (PTSD), and psychological distress [9, 10, 11]. Furthermore, these mental health disorders are associated with higher rates of hospitalization, prolonged hospital stays, physical distress, and poorer treatment compliance, which can significantly affect parents' ability to engage in their daily activities and fulfill their roles and responsibilities [12, 13]. The impact of perinatal loss extends beyond immediate emotional and psychological consequences for parents, as it can also have long-term effects on their physical health, relationships, and overall well-being. Previous studies have shown that parents who have experienced perinatal loss often report high levels of insomnia [14], marital strain (e.g., poor sexual intimacy) [15], social isolation [16], and reduced well-being and quality of life [17].

Recognizing the significant impact of perinatal loss on parents and families, there has been a growing interest in understanding and addressing the needs of those affected. Nonpharmacological interventions, such as counseling [18], cognitive-based interventions (including cognitive behavioral therapy (CBT; [6] and relative third-wave therapies [19]), supportive interventions [20], and mind–body exercises [21], have emerged as potential approaches to support parents in coping with their emotional difficulties and mental health disorders and promoting their psychological well-being. These interventions aim to provide emotional and informational support, facilitate the expression of grief, enhance coping skills, and promote resilience/personal growth in parents who have experienced perinatal loss.

While there is a growing body of literature on nonpharmacological interventions for perinatal loss, there is still a need for a comprehensive synthesis of the existing evidence. However, there is a lack of systematic reviews that include randomized controlled trials (RCTs). Only two systematic reviews were identified that included RCT to explore the effectiveness of psychosocial interventions on the psychological outcomes of parents with perinatal loss. The systematic review performed by [22] included 6 RCTs and showed no significant follow-up effect on psychological well-being, including anxiety, grief, depression avoidance, and self-blame. However, the latest systematic review and meta-analysis by [23] showed inconsistent findings. This study included a total of 17 RCTs in the systematic review and 15 RCTs in the meta-analysis and demonstrated that psychosocial interventions significantly reduced depression for parents with perinatal loss after a 6-week follow-up. Additionally, this study found that psychosocial interventions significantly reduced parental depression, anxiety, and grief for parents with perinatal loss postintervention, compared to control groups [23]. However, these reviews had some methodological limitations. First, it is important to note that previous reviews did not explore the effectiveness of nonpharmacological interventions on key mental health outcomes, such as PTSD and psychological distress, as well as well-being outcomes, including sleep quality and social support. Therefore, the impact of these interventions on these specific outcomes remains unclear. Second, the optimal characteristics of nonpharmacological interventions, such as intervention approaches, delivery formats, and ideal intervention sessions, remain unknown. Therefore, further research is needed to address these gaps in the literature and provide a more comprehensive understanding of the potential benefits of nonpharmacological interventions for parents experiencing perinatal loss.

Given the high prevalence of perinatal loss and the significant emotional difficulties and mental health disorders experienced by parents, the aim of this study is to conduct a comprehensive assessment and synthesis of nonpharmacological interventions' effects on enhancing the mental health and well-being of parents (both mothers and fathers) who have encountered perinatal loss. Additionally, this study seeks to identify the key characteristics and elements of successful interventions in order to inform healthcare professionals, policymakers, and researchers in the development and implementation of evidence-based interventions that address the unique needs of this population.

## 2. Materials and Methods

This study follows the guidelines of the Preferred Reporting Items for Systematic Reviews and Meta-Analysis (PRISMA) [24] and is conducted according to The Cochrane Handbook for Systematic Reviews of Interventions [25]. Moreover, this review was formally registered in the PROSPERO International Prospective Register of Systematic Reviews (CRD42023483291).

### 2.1. Research Strategy

A systematic search was conducted from January 2008 to 19 December 2023 across seven English electronic databases/registers, specifically PubMed, Embase (Ovid), Scopus, Cumulated Index to Nursing and Allied Health Literature (CINAHL), PsycINFO, ProQuest, and Cochrane Central Register databases. The keywords and Medical Subject Headings (MeSH) were selected in accordance with the PICOS (Population, Intervention, Comparison, Outcomes, and Study) design framework (Table S1). Table S2 displays the search procedures and results for all databases/registers. Furthermore, we carried out manual examinations of the reference lists of pertinent articles and did an online search using Google Scholar. In order to enhance the inclusivity of our findings and mitigate the possibility of publication bias, the researchers implemented the following measures [25].

### 2.2. Eligibility Criteria

The eligible criteria are displayed in Table 1.

### 2.3. Study Selection

The search results from all databases were imported into Endnote (Version 20 for MAC) for the screening process. Following the removal of duplicates, first, two authors independently assessed the titles and abstracts for relevance based on the eligibility criteria outlined in Table 1. Second, in cases where a study was deemed potentially relevant, the full text was retrieved for further evaluation of eligibility. Any conflicts or discrepancies between the two reviewers were resolved through discussion, and a third reviewer was consulted if necessary to reach a consensus [26].

### 2.4. Data Extraction

Two authors independently gathered data, which included fundamental study details (e.g., name of authors, year of publication, location, study design, and number of participants each group), participant characteristics (e.g., type of perinatal loss, age), intervention characteristics/elements (e.g., recipient, content, modality, format, intensity, time points of data collection, retention rate, setting, provider, and theoretical framework), outcome measures, and statistical results (e.g., mean and standard deviation (SD)). This information was collected using a self-developed form based on the Cochrane data collection form for RCT review [27]. The first author emailed the corresponding authors to request clarification in cases where the information presented in their publications was lacking or ambiguous. The publications of authors who did not provide a response were excluded from the review or meta-analysis. All conflicts that arose during this procedure were effectively addressed through deliberation between the two evaluators.

### 2.5. Quality Appraisal and Certainty of Evidence

According to Sterne et al. [28], the updated Cochrane Risk of Bias instrument (RoB2) for RCTs was utilized to evaluate the quality assessment of the published studies. This tool evaluates the risk for bias in the following five areas: randomization process, deviations from the intended intervention, missing outcome data, measurement of the outcome, and selection of the reported result. The risk of bias is categorized as “low,” “unclear,” or “high.” The certainty of evidence was evaluated utilizing the Grading of Recommendation, Assessment, Development, and Evaluation (GRADE) profiler Guideline Creation Tool [29], which categorizes the certainty of evidence as high, moderate, low, and very low. The quality appraisals were conducted by two authors independently, and any inconsistencies were handled through consensus between them.

### 2.6. Data Synthesis

The “meta” and “metaphor” packages in the R software were employed for the purpose of conducting meta-analytical studies. The utilization of these software packages enabled the computation of Hedges' g standard mean differences (SMDs) in conjunction with 95% confidence intervals (CIs) for every study. The meta-analytical analysis utilized the random-effects model. The effect sizes were determined by the comparison of group differences and were classified into three categories: small (0.2 ≤ *d* < 0.5), medium (0.5 ≤ *d* < 0.8), or large (*d* ≥ 0.8) effect size based on Cohen's criterion [30]. Furthermore, the scores associated with parental sleep quality and perceived social support were reversed for clarity. This determination of the effect direction ensured that positive effect sizes were always associated with positive clinical results, while negative effect sizes were always associated with negative clinical results (e.g., grief, stress, PTSD, depression, anxiety, and distress).

To evaluate the heterogeneity among the included studies, we calculated the *I*^2^ and Cochrane's *Q* statistics. A distribution of effect sizes was considered heterogeneous if the *I*^2^ statistic exceeded 50% and the *p*-value was ≥0.05 [25]. The meta-analysis incorporated five subgroup studies to investigate the source of heterogeneity. The purpose of these analyses was to assess whether the effectiveness of interventions differed among various approaches (such as cognitive-based interventions, counseling, supportive interventions, and mind–body exercises), delivery modalities (such as face-to-face, eHealth, and blended), delivery formats (individual-based versus group-based formats), types of perinatal loss (including miscarriage, stillbirth, TOPFA, mixed types, and recurrent miscarriage), and the number of intervention sessions (≤four sessions versus >four sessions). The Knapp–Hartung adjustment was performed for the meta-analyses if the number of studies in a group or subgroup was ≤10.

Sensitivity analyses were conducted by removing one study at a time to determine if the exclusion of studies with significantly different effect sizes had a substantial impact on the overall results. Additionally, Egger's test and visual inspection of funnel plots were used to evaluate publication bias in meta-analyses with 10 or more studies. An asymmetrical funnel plot indicated possible publication bias and a *p*-value <0.05 in Egger's test was considered statistically significant evidence of asymmetry [31]. Outliers were identified by visually examining the forest plots, and their inclusion was considered if their removal did not have a significant impact on the outcomes. In cases where trials or outcomes were excluded from the meta-analyses, a narrative synthesis was conducted instead.

## 3. Results

### 3.1. Study Selection

The process of selecting studies is illustrated in the PRISMA flow diagram (refer to Figure S1), which provides a depiction of the inclusion and exclusion of articles at each stage. Initially, 5,77 duplicate records were eliminated out of a total of 3,070 records that were found. Following the evaluation of the titles and abstracts of the remaining 2,493 articles, a total of 59 articles were chosen for subsequent examination of their full-text content. Thirty-seven reports were excluded for various reasons, such as not being an RCT, being published more than 15 years ago, including pregnant parents, lacking relevant outcomes, not being published in English, focusing on perinatal loss beyond 1 year, or involving parents whose child died after the neonatal phase. Ultimately, this review incorporated a total of 23 publications, which consisted of 21 trials. This count includes an RCT that reported two outcomes (grief and PTSD) in two separate publications [32, 33], while another RCT was reported in both a journal article [34] and a dissertation [35].

### 3.2. Risk of Bias

The evaluation of the included studies revealed that each study had “some concerns” regarding its quality, as shown in Figure S2. Specifically, 12 trials were found to have “some concerns” related to bias in the randomization process, primarily due to insufficient information about allocation concealment. On the other hand, the remaining nine trials were assessed as having a “low” risk of bias. Deviations from the intended intervention were observed in all trials. Regarding missing data, two trials had “some concerns” due to insufficient information, while the other 19 trials were considered to have a “low” risk of bias in this aspect. The use of self-reported instruments in all trials raised concerns about outcome measurement. Two trials were found to have “some concerns” regarding the selection of reported results, mainly due to inadequate information in the prespecified proposal. In contrast, the remaining 21 trials were assessed as having a “low” risk of bias in this regard.

### 3.3. Study Characteristics

The characteristics of the 21 trials included in the study were reported in Table S3. The period of publication spanned from 2009 until 2023. The study encompassed a variety of locales, including United States (*n* = 5) [21, 34, 35, 36, 37, 38], China (*n* = 5) [39, 40, 41, 42, 43], Iran (*n* = 4) [18, 20, 32, 33, 44], Germany (*n* = 2) [6, 45], Denmark (*n* = 1) [19], France (*n* = 1) [46], India (*n* = 1) [47], Portugal (*n* = 1) [48], and Turkey (*n* = 1) [49]. Three of the 21 RCTs were pilot trials [21, 36, 40]. Eighty percent of the trials used 2-arm designs (*n* = 18), whereas one was a 3-arm pilot RCT [21] and another study was a four-arm RCT [38].

### 3.4. Characteristics of the Participants

All 21 trials reported the number of participants, which comprised a total of 2,430 participants, with 1,210 participants in the treatment group and 1,220 participants in the control group. The sample sizes varied from 40 [34, 35] to 341 [38] (mean: 115.71; SD: 75.73; median: 91). The mean age of parents with perinatal loss ranged from 18.29 (SD: 8.87) [20] to 34.71 years (SD: 4.58) [39]. Sixteen trials only involved mothers as targeted participants, while four trials included both mothers and fathers as the targeted population, with the percentage of male participants ranging from 8% to 50%. Moreover, one study included family members to provide social support for women who experienced perinatal loss [42]. Five different types of perinatal loss were identified among the participants, including miscarriage (*n* = 7), stillbirth (*n* = 4), TOPFA (*n* = 4), recurrent miscarriage (*n* = 3), and mixed types (*n* = 3).

### 3.5. Characteristics of the Interventions

The study identified five nonpharmacological intervention approaches: cognitive-based interventions (*n* = 7), counseling (*n* = 7), supportive interventions (*n* = 4), mind–body exercise (*n* = 2), and interpersonal psychotherapy (*n* = 1). Cognitive-based interventions utilized techniques such as CBT, mindfulness, meditation, metaphors, and education related to perinatal loss and coping skills. Supportive interventions primarily focused on providing emotional and informational support for parents experiencing perinatal loss. Two components of mind–body exercise, namely mindfulness/meditation and Yoga, were also identified. Moreover, nine trials included in this review applied various theoretical frameworks to guide the intervention development. These frameworks/models/theories included Worden's principles of grief, CBT theory, Roy adaptation model, Ottawa decision framework, cognitive narrative model, Betty Neuman's systems model, Psychological stress theory, Swanson's caring theory, meaning of miscarriage model, and transactional model of stress and coping.

The interventions spanned between 1 day and 12 weeks, with a mean of 37 days (SD: 28.61, median: 35). However, five trials did not provide information on the duration. The number of intervention sessions varied from 1 to 14 sessions, with an average of 5 sessions (SD: 4.06, median: 4). However, five trials did not specify the number of sessions. On average, the length of intervention sessions was 79 min (SD: 28.96, median: 67.5), ranging from 0.5 to 2 hr. However, information on session length was not disclosed in the majority of trials (*n* = 13). Additionally, seven trials did not report the interval between intervention sessions. Among the trials that did report this information, the majority conducted interventions on a weekly basis (*n* = 9), while two trials had a monthly interval (*n* = 2), and three trials had a 1-day intervention without a specific interval.

There were three main delivery modalities for the interventions: face-to-face (*n* = 10), eHealth (*n* = 5, including DVD and online platforms), and blended (*n* = 6, combining face-to-face and eHealth formats). A total of 17 research employed an individual format for delivering interventions, whereas three trials utilized a group format, and one study employed a blended format that combined individual and group sessions. The nonpharmacological interventions were administered by trained professionals such as nurses, midwives, psychologists, doctors specializing in habitual abortion, or Yoga teachers. The intervention settings exhibited a range of diversity, encompassing hospital/clinic (*n* = 15), participant's home (*n* = 1), and online platforms (*n* = 5). The mean retention rate of the included trials was 85.96% (median: 91.94%, SD: 0.13, range: 53.33%–100%) at postintervention assessment and 75.69% (median: 76.43%, SD: 0.12, range: 63.6%–100%) at the complete follow-up assessment.

### 3.6. Characteristics of the Control Groups

Nineteen trials incorporated inactive control groups, wherein the control groups were assigned to receive routine care (*n* = 15), being on a waitlist (*n* = 3), or receiving a placebo (*n* = 1). The two remaining trials incorporated active control groups, with one group focusing on coping with depression and the other on stretch and tone exercises.

### 3.7. Outcome Measures

The measurement scales utilized in the trials included in this analysis are presented in Table S4, and each scale was a questionnaire that had been standardized and verified. The outcomes were divided into two main categories: (1) psychological outcomes, which included measures of parental grief, stress, PTSD, depression, anxiety, and distress; and (2) well-being outcomes, which included measures of sleep quality and perceived social support.

All of the included trials utilized multiple time points to measure results. Moreover, while a large portion of the trials (*n* = 18) included pre- and posttest assessments, three trials solely conducted a posttest or follow-up test. One study included a short-term follow-up assessment at 1.5 months, five trials used a medium-term follow-up assessment ranging from 3 to 6 months, and five trials used a long-term follow-up assessment of 12 months or more.

### 3.8. The Results of Meta-Analysis

A total of 19 studies were included in the meta-analyses, as two trials were unable to obtain data outcomes based on mean and SD [36, 38].  Table 2 provides an overview of the main findings of the meta-analysis.

#### 3.8.1. Parental Grief

Ten trials involving 976 participants were analyzed, revealing that nonpharmacological interventions significantly reduced grief levels postintervention for parents with perinatal loss postintervention, compared to the control groups (Hedges' *g* = −0.68, 95% CI: (−0.98, −0.38), *p*-value for chi-squared test <0.01, *I*^2^ = 79%; Figure S3). No publication bias was detected based on the funnel plot and Egger's test (*t* = −2.08, *p*=0.072; Figure S5).

#### 3.8.2. Parental Stress

Five trials with 388 participants showed that nonpharmacological interventions did not significantly reduce parental stress for parents with perinatal loss postintervention when compared to control groups (*g* = −1.34, 95% CI: (−2.75, 0.07), *p* < 0.01, *I*^2^ = 96%; Figure S3).

#### 3.8.3. Parental PTSD

Five trials with 532 participants indicated that nonpharmacological interventions significantly alleviated parental PTSD symptoms for parents with perinatal loss postintervention when compared to control groups (*g* = −0.69, 95% CI: (−0.92, −0.46), *p*=0.21, *I*^2^ = 31%; Figure S3).

#### 3.8.4. Parental Depression

Fifteen trials with 1,521 participants reported a medium effect of nonpharmacological interventions in reducing parental depression postintervention when compared to control groups (*g* = −0.63, 95% CI: (−0.95, −0.30), *p* < 0.01, *I*^2^ = 87%; Figure S3). No potential publication bias was seen based on the results of the funnel plot and Egger's test (*t* = −1.12, *p*=0.28; Figure S6).

#### 3.8.5. Parental Anxiety

Ten trials with 960 participants showed that nonpharmacological interventions significantly decreased anxiety levels for parents with perinatal loss postintervention when compared to control groups (*g* = −0.72, 95% CI: (−1.19, −0.24), *p* < 0.01, *I*^2^ = 89%; Figure S4). No potential publication bias was observed based on the results of the funnel plot and Egger's test (*t* = −0.54, *p*=0.61; Figure S7).

#### 3.8.6. Parental Distress

Three trials with 474 participants found no significant improvement in distress levels for parents with perinatal loss postintervention (*g* = −1.89, 95% CI: (−4.08, 0.31), *p*  < .01, *I*^2^ = 98%; Figure S4).

#### 3.8.7. Parental Sleep Quality

This outcome was measured in four trials with 214 participants. The pooled results showed that nonpharmacological interventions did not significantly improve sleep quality for parents with perinatal loss postintervention when compared to control groups (*g* = 0.19, 95% CI: (−0.34, 0.73), *p*=0.01, *I*^2^ = 72%; Figure S4).

#### 3.8.8. Perceived Social Support

Five trials with 333 participants showed a small positive effect of nonpharmacological interventions on perceived social support for parents with perinatal loss postintervention when compared to control groups (*g* = 0.31, 95% CI: (0.08, 0.55), *p*=0.31, *I*^2^ = 16%; Figure S4).

### 3.9. Sensitivity Analysis

The sensitivity analyses conducted in this meta-analysis revealed that the results pertaining to parental grief, PTSD, depression, anxiety, and sleep quality were robust and consistent. Nevertheless, a degree of variability was observed in the evaluations of parental stress, distress, and perceived social support, where positive effects were either observed or reversed with the exclusion of certain studies. The results of all sensitivity analyses are presented in Figures S8, S9, S10, S11, S12, S13, S14, and S15.

### 3.10. Subgroup Analysis

Tables S5, S6, S7, S8, and S9 and Figures S16, S17, S18, S19, S20, S21, S22, S23, S24, S25, S26, S27, S28, S29, S30, S31, S32, S33, S34, S35, S36, S37, S38, S39, S40, S41, S42, S43, S44, S45, and S46 contain the outcomes of the five-subgroup analysis.

#### 3.10.1. Comparison of Treatment Effects among Four Intervention Approaches

The results of subgroup analyses showed that cognitive-based interventions were the most effective approach for parents with perinatal loss compared to counseling, supportive interventions, and mind–body exercise. Cognitive-based interventions significantly decreased parental grief (*k*_number of trials_ = 4, *g* = −0.58, 95% CI: (−0.77, −0.40), *p*=0.34, *I*^2^ = 11%), stress (*k* = 2, *g* = −1.10, 95% CI: (−2.10, −0.11), *p*  < 0.01, *I*^2^ = 89%), PTSD (*k* = 3, *g* = −0.71, 95% CI: (−1.03, −0.38), *p*=0.09, *I*^2^ = 58%), depression (*k* = 5, *g* = −0.66, 95% CI: (−0.90, −0.42), *p*=0.13, *I*^2^ = 44%), and anxiety (*k* = 5, *g* = −0.63, 95% CI: (−1.06, −0.20), *p*  < 0.01, *I*^2^ = 81%) postintervention in parents with perinatal loss. Counseling significantly decreased depression (*k* = 6, g = −0.74, 95% CI: (−1.42, −0.06), *p*  < 0.01, *I*^2^ = 92%) and improved perceived social support (*k* = 2, *g* = 0.38, 95% CI: (0.05, 0.71), *p*=0.50, *I*^2^ = 0%), while supportive interventions significantly reduced depression (*k* = 2, *g* = −0.42, 95% CI: (−2.04, 1.19), *p*  < 0.01, *I*^2^ = 96%) in these parents.

#### 3.10.2. Comparison of Treatment Effects among Three Intervention Modalities

When comparing three intervention modalities, face-to-face nonpharmacological interventions were more effective in alleviating parental grief (*k* = 4, *g* = −0.66, 95% CI: (−0.91, −0.41), *p*=0.26, *I*^2^ = 25%), stress (*k* = 3, *g* = −1.66, 95% CI: (−3.30, −0.02), *p*  < 0.01, *I*^2^ = 96%), depression (*k* = 7, *g* = −0.92, 95% CI: (−1.49, −0.35), *p*  < 0.01, *I*^2^ = 89%), anxiety (*k* = 6, *g* = −1.08, 95% CI: (−1.72, −0.44), *p*  < 0.01, *I*^2^ = 90%), and distress (*k* = 2, *g* = −2.80, 95% CI: (−5.01, −0.60), *p*  < 0.01, *I*^2^ = 97%). In contrast, the implementation of eHealth nonpharmacological therapies demonstrated efficacy in mitigating parental grief (*k* = 4, *g* = −0.51, 95% CI: (−0.97, −0.04), *p*  < 0.01, *I*^2^ = 83%), PTSD (*k* = 3, *g* = −0.87, 95% CI: (−1.09, −0.65), *p*=0.99, *I*^2^ = 0%), and anxiety (*k* = 3, *g* = −0.38, 95% CI: (−0.59, −0.16), *p*=0.52, *I*^2^ = 0%). The blended modality of nonpharmacological interventions significantly decreased depression (*k* = 3, *g* = −0.33, 95% CI: (−0.58, −0.07), *p*=0.23, *I*^2^ = 31%) and increased perceived social support (*k* = 2, *g* = 0.57, 95% CI: (0.23, 0.90), *p*=0.53, *I*^2^ = 0%) postintervention for parents who experienced perinatal loss.

#### 3.10.3. Comparison of Treatment Effects between Two Intervention Formats

Based on the available evidence, individual-based interventions were found to be effective in alleviating parental grief (*k* = 9, *g* = −0.67, 95% CI: (−1.01, −0.33), *p*  < 0.01, *I*^2^ = 81%), PTSD (*k* = 5, *g* = −0.69, 95% CI: (−0.92, −0.46), *p*=0.21, *I*^2^ = 31%), depression (*k* = 14, *g* = −0.59, 95% CI: (−0.94, −0.25), *p*  < 0.01, *I*^2^ = 88%), and anxiety (*k* = 9, *g* = −0.63, 95% CI: (−1.12, −0.13), *p*  < 0.01, *I*^2^ = 88%), as well as in improving perceived social support (*k* = 5, *g* = 0.31, 95% CI: (0.08, 0.55), *p*=0.31, *I*^2^ = 16%). In contrast, the impact of group-based therapies on mitigating parental stress was shown to be statistically significant (*k* = 2, *g* = −1.10, 95% CI: (−2.10, −0.11), *p*  < 0.01, *I*^2^ = 89%) postintervention for these group of parents.

#### 3.10.4. Comparison of Treatment Effects among Five Types of Perinatal Loss

Subgroup analysis revealed significant effects of nonpharmacological interventions on parental grief (*k* = 2, *g* = −0.73, 95% CI: (−1.10, −0.36), *p*=0.14, *I*^2^ = 0%), stress (*k* = 2, *g* = −2.83, 95% CI: (−5.25, −0.40), *p*  < 0.01, *I*^2^ = 97%), depression (*k* = 5, *g* = −1.07, 95% CI: (−1.85, −0.29), *p*  < 0.01, *I*^2^ = 95%), and anxiety (*k* = 3, *g* = −1.66, 95% CI: (−2.55, 0.77), *p*  < 0.01, *I*^2^ = 90%) when the interventions included parents with miscarriage. Significant enhancements were observed in nonpharmacological therapies for parental stress (*k* = 4, *g* = −0.58, 95% CI: (−1.08, −0.07), *p*  < 0.01, *I*^2^ = 85%) and anxiety (*k* = 2, *g* = −0.43, 95% CI: (−0.78, −0.08), *p*=0.33, *I*^2^ = 0%) in interventions involving parents with stillbirth. Significant enhancements were observed in PTSD (*k* = 2, *g* = −0.43, 95% CI: (−0.72, −0.13), *p*=0.66, *I*^2^ = 0%) and depression (*k* = 3, *g* = −0.44, 95% CI: (−0.69, −0.18), *p*=0.78, *I*^2^ = 0%) in therapies involving parents with TOPFA. In interventions involving parents with mixed types of perinatal loss, significant enhancements were identified for grief (*k* = 2, *g* = −0.59, 95% CI: (−0.82, −0.37), *p*=0.58, *I*^2^ = 0%), PTSD (*k* = 2, *g* = −0.87, 95% CI: (−1.10, −0.64), *p*=0.88, *I*^2^ = 0%), depression (*k* = 2, *g* = −0.67, 95% CI: (−0.90, −0.44), *p*=0.61, *I*^2^ = 0%), and anxiety (*k* = 2, *g* = −0.40, 95% CI: (−0.63, −0.18), *p* = 0.35, *I*^2^ = 0%). In interventions involving parents with recurrent miscarriage, significant enhancements were identified for stress (*k* = 3, *g* = −0.37, 95% CI: (−0.67, −0.07), *p* = 0.36, *I*^2^ = 3%) postintervention.

#### 3.10.5. Comparison of Treatment Effects between Two Intervention Sessions Numbers

The results of the subgroup analysis indicated that a minimum of four sessions of nonpharmacological therapies was considered favorable. Intervention session of four or less significantly decreased parental grief (*k* = 6, *g* = −0.80, 95% CI: (−1.20, −0.40), *p*  < 0.01, *I*^2^ = 70%), stress (*k* = 2, *g* = −1.10, 95% CI: (−2.10, −0.11), *p*  < 0.01, *I*^2^ = 89%), PTSD (*k* = 3, *g* = −0.71, 95% CI: (−1.03, −0.38), *p*=0.09, *I*^2^ = 58%), depression (*k* = 10, *g* = −0.74, 95% CI: (−1.14, −0.32), *p*  < 0.01, *I*^2^ = 88%), and anxiety (*k* = 5, *g* = −0.80, 95% CI: (−1.37, −0.23), *p*  < 0.01, *I*^2^ = 89%), as well as effectively boosted perceived social support (*k* = 2, *g* = 0.38, 95% CI: (0.05, 0.71), *p*=0.50, *I*^2^ = 0%). In contrast, interventions that involved more than four nonpharmacological sessions resulted in significant improvements in depression (*k* = 2, *g* = −1.03, 95% CI: (−1.38, −0.68), *p*=0.76, *I*^2^ = 0%) for parents with perinatal loss postintervention.

### 3.11. Narrative Synthesis

Two included studies conducted narrative synthesis as they did not report mean and SD values in their studies [36, 38]. Swanson et al. [38] conducted a four-arm RCT and reported that a three-session nurse counseling had the largest effect on resolving grief (women: Bayesian odds, ranged 3.1–7.0; men: 18.7–22.6) and depression (women: 3–7.9; men: 3.2–6.6) for couples who experienced miscarriage, compared to the no treatment control group. The combined care approach, which included one counseling session plus three self-care modules, had the second largest effect, while the three self-care modules alone had the least preferred approach. Similar results were reported in Johnson et al. [36], where a blended (consisting of one individual session, 12 group-based sessions, and one individual booster after 1 month) interpersonal psychotherapy was found to reduce maternal grief (*d* = −0.33) compared to those in the coping with depression control group. However, no significant effects were identified in alleviating maternal depression and couple distress, as well as boosting social support.

### 3.12. Certainty of the Evidence

The outcomes in the included trials (*n* = 21) were assessed using a GRADE scale, which ranged from “High” to “Low” certainty, as shown in Table 2. Nonpharmacological interventions were found to have a “high” certainty of evidence for reducing parental PTSD (*n* = 5). The evidence regarding parental grief (*n* = 10), depression (*n* = 15), anxiety (*n* = 10), distress (*n* = 3), and perceived social support (*n* = 3) was ranked as “moderate” certainty. This ranking was attributed to inconsistencies (e.g., high heterogeneity) or imprecision (e.g., small sample sizes) in the findings. On the other hand, the evidence on parental stress (*n* = 5) and sleep quality (*n* = 4) was evaluated as having “low” certainty primarily due to the aforementioned inconsistencies and imprecision.

## 4. Discussion

This systematic review and meta-analysis included 23 studies, which covered 21 RCTs, while among them, 19 trials were further included in the meta-analysis. This systematic review and meta-analysis involved a total of 2,430 participants who assessed the effectiveness of nonpharmacological interventions in improving grief, stress, PTSD, depression, anxiety, distress, sleep quality, and perceived social support among parents who have experienced perinatal loss, in comparison to active/inactive control groups, while also identifying optimal intervention characteristics/components.

Results from the meta-analysis indicate that nonpharmacological interventions are effective in alleviating parental psychological symptoms, including grief, PTSD, depression, and anxiety, while also increasing perceived social support postintervention when compared to active/inactive control groups. These findings are consistent with previous reviews conducted by Huberty et al. [50] and Shaohua and Shorey [23]. Huberty et al. [50] performed a systematic review of two articles and reported that psychosocial interventions effectively reduced grief in women with stillbirth. This finding was further supported by Shaohua and Shorey. [23], who conducted a systematic review of 17 RCTs (of which 15 RCTs were assessed in meta-analysis) and found that psychosocial interventions significantly alleviated grief (Cohen's *d* = 0.51), depression (*d* = 0.46) and anxiety (*d* = 0.34) for parents with perinatal loss postintervention, compared to inactive control groups. Moreover, our study also identified that nonpharmacological interventions significantly decreased PTSD and enhanced perceived social support for parents with perinatal loss postintervention, which was not explored in previous systematic reviews of RCTs. Previous experimental evidence has shown that insufficient informal (support from partners, family members, friends, colleagues, and significant others) and formal (support from health professionals and services) social support is associated with a higher risk of PTSD, depression, and anxiety symptoms [51, 52, 53]. Therefore, our findings imply that providing social support, both formal and informal, in terms of informational and emotional support, to parents with perinatal loss can help buffer against these mental health disorders [54]. However, it is crucial to acknowledge that the findings about the effect of nonpharmacological interventions on perceived social support were unstable, necessitating further meticulously designed RCTs to validate their effectiveness. Furthermore, while this analysis offers a thorough examination of the overall efficacy of nonpharmacological therapies in mitigating psychiatric symptoms and perceived social support among parents experiencing perinatal loss, we did find significant statistical heterogeneity. Therefore, further subgroup analyses were performed in our study to explore the sources of these heterogeneities.

### 4.1. Optimal Intervention Approaches

Interestingly, our subgroup analysis revealed that cognitive-based interventions are the most effective approach for improving grief, stress, PTSD, depression, and anxiety in parents with perinatal loss, when compared to counseling, supportive interventions, and mind–body interventions. This result echoed with a previous systematic review and meta-analysis that included eight RCTs, indicating that internet-based CBT significantly improved stress, anxiety, and depressive symptoms in postpartum women compared to inactive control groups [55]. Specifically, these included cognitive-based interventions in our review not only provide emotional support for parents with perinatal loss, such as using CBT, mindfulness-based interventions, or psychological support to help them coping with their emotional difficulties but also equip them with information or knowledge related to perinatal loss [44, 46]. These findings are consistent with previous experimental and theoretical evidence that providing combined emotional/psychological and informational supports can affect negative appraisals and the emotional and neuroendocrine stress response [52, 56]. Furthermore, considering the needs and challenges of physical recovery for parents with perinatal loss, although existing evidence of mind–body interventions did not significantly reduce symptomatology for parental with perinatal loss due to a limited number of included trials, it is important for studies to consider how to effectively combine postnatal exercise (e.g., low-to-moderate exercises, such as Yoga), into cognitive-based interventions to help mothers in physical recovery [57] and enhance sleep quality [40]. Nevertheless, when it comes to cognitive-based therapies for parents experiencing perinatal loss, it remains notably difficult to differentiate the benefits of various intervention components from one another. Therefore, it is recommended that future research endeavors focus on investigating the impacts of cognitive-based therapies and, of equal significance, the underlying mechanisms that contribute to their efficacy across diverse groups.

### 4.2. Optimal Modality and Format of Implementation

The subgroup analysis found that parents who experienced perinatal loss may benefit more from face-to-face nonpharmacological interventions compared to eHealth or blended modalities in terms of parental grief, stress, depression, anxiety, and distress. However, eHealth interventions were found to significantly alleviate parental grief, PTSD, and anxiety, while blended interventions showed significant improvements in parental depression and perceived social support. These findings are in line with a study by Shaohua and Shorey [23], which demonstrated that both face-to-face and technology-based psychosocial interventions were effective in reducing grief, depression, and anxiety in parents with perinatal loss. Indeed, the effectiveness of face-to-face, eHealth, and blended interventions in reducing psychological problems for parents experiencing perinatal loss can be attributed to their ability to provide tailored support that addresses the diverse needs of this population. Face-to-face interventions allow direct observation and identification of clinically relevant behaviors in therapy that help to contextualize and provide the client with the appropriate exercise according to the clinical conversation [58]. On the other hand, eHealth/internet-based interventions offer advantages such as flexibility in time and location, better treatment adherence, cost saving for both service providers and parents, and visual anonymity [59], while blended models combine the benefits of both to deliver comprehensive and continuous care. Thus, considering the benefits of both modalities and the feasibility and effectiveness of blended nonpharmacological interventions for parents with perinatal loss, future studies are recommended to explore the effectiveness of nonpharmacological interventions in this population.

In addition, a subgroup analysis showed that individual-based nonpharmacological interventions significantly improved parental grief, PTSD, depression, anxiety, and perceived social support in parents with perinatal loss postintervention. On the other hand, group-based nonpharmacological interventions revealed potential effectiveness in reducing parental stress. This difference can be attributed to the personalized support provided in an individual-based format, which caters to the unique needs and circumstances for each parent [60]. This format also offers healthy coping strategies tailored to each individual and family, such as postpartum guidance, self-care practices, relaxation techniques, and stress management strategies [42]. Furthermore, individual-based interventions provide a safe space for parents to process their emotions, which is crucial for healing and progressing through the grieving process [61]. Nevertheless, group-based interventions offer distinct benefits, such as facilitating the exchange of knowledge, enabling parents experiencing perinatal loss to share experiences with peers, and providing mutual support in overcoming challenges [44]. Thus, it is advised that further research should be conducted to verify the efficacy of various intervention formats in order to provide more practical, acceptable, affordable, accessible, and economical means of supporting parents who have experienced perinatal loss.

### 4.3. Effectiveness of Interventions for Different Types of Perinatal Loss

Considering the diverse experiences and meanings associated with different types of perinatal loss, we conduct a subgroup analysis focusing on different types of perinatal loss to inform future study design. The results of the subgroup analysis showed the effectiveness of nonpharmacological interventions in reducing grief, stress, depression, and anxiety among parents with miscarriages, while these interventions are also found to be effective in decreasing stress among parents with recurrent miscarriages. Notably, couple-focused interventions were particularly beneficial for these parents, as they addressed the psychological difficulties faced by both men and women [6, 37, 38, 45]. By targeting the needs of both partners, these interventions enhanced their capacity and resources to provide support to one another (especially to women), as evidenced by therapist responses and couples' interactions [62]. Furthermore, previous evidence-based management for recurrent miscarriages has highlighted the importance of lifestyle modifications, such as abstaining from tobacco, alcohol, and illicit drugs, maintaining a healthy diet, engaging in regular exercise, getting sufficient sleep, and managing stress and grief [62]. These lifestyle changes have been shown to improve the chances of a successful pregnancy for couples experiencing recurrent miscarriage.

Furthermore, nonpharmacological interventions have demonstrated effectiveness in reducing PTSD and depression in parents who decide to undergo TOPFA. These interventions have also been found to be effective in reducing grief, PTSD, depression, and anxiety among parents with mixed types of perinatal loss, including TOPFA. This finding aligns with Shaohua and Shoreyʼs [23] study, which also revealed the effectiveness of psychosocial interventions in reducing depression among parents who decide to TOP. This emphasizes the importance of regular monitoring during pregnancy and the provision of medical information. Providing parents with information about the cause of prenatal abnormalities is crucial in helping them understand the situation, enhancing their perception of psychological support and guidance, and minimizing long-term mental health disorders [63]. Equipping individuals with sufficient knowledge can aid in emotional adaptation [23]. Therefore, expanding the availability of medical information to cover various types of perinatal losses may be beneficial. Additionally, one of the studies included in our review implemented a family support program to reduce depression and PTSD among women who chose TOPFA, highlighting the significance of social support provided by both healthcare providers and family members in facilitating women's physical and psychological recovery [42].

In general, lifestyle modification, social support, particularly from partners and family members, the provision of medical information, and psychological support are beneficial for parents experiencing different types of perinatal loss. These elements facilitate physical and psychological recovery, cognitive reappraisal, knowledge acquisition, and help parents overcome challenging times. Therefore, future nonpharmacological interventions should focus on addressing these aspects to provide better support for parents experiencing perinatal loss.

### 4.4. Favorable Number of Sessions

Additionally, our subgroup analysis revealed that nonpharmacological interventions with a maximum of four sessions were found to significantly improve grief, stress, PTSD, depression, anxiety, distress, and perceived social support among parents with perinatal loss postintervention, compared to interventions with more than four sessions. However, interventions with more than four sessions were found to have a significantly larger effect on depression for these parents. These findings contradict the results of a study by Shaohua and Shorey [23], which found no significant difference in the efficacy of single- and multisession psychosocial interventions in reducing depression and anxiety among parents with perinatal loss postintervention. Based on previous evidence, it is recommended to provide timely interventions for parents with perinatal loss, with the first session typically scheduled within the first or second day after loss [20, 34, 35, 38, 42]. These interventions should ideally consist of one to four sessions and be followed up with postdischarge support to promote the maintenance of therapeutic rapport, continuity of care, and reinforcement [34, 41, 45, 48]. It is important to note that longer treatment durations may result in a decline in the dose–response effect [46]. Therefore, more RCTs with different durations are recommended to ascertain the positive dose–response amongst parents with different perinatal loss and on different parental outcomes.

### 4.5. Study Limitations and Implications

This study has several limitations that should be acknowledged. First, our search was limited to English databases, which may have introduced publication bias. Second, the included studies had methodological shortcomings, namely in terms of insufficient data on concealment, intervention procedure/manual, and intention-to-treat analysis. Moreover, the utilization of self-report questionnaires to assess outcomes may have added potential bias. The presence of these constraints diminishes the overall quality of the evidence and heightens the potential for bias in our findings. Thirdly, the limited number of studies included in the analysis introduces further uncertainty concerning specific outcomes, including parental stress, distress, and sleep quality. However, the pooled results for these outcomes showed promising results for nonpharmacological interventions in reducing parental stress and distress, with positive outcomes observed after removing outliers. Future studies should use well-designed RCTs to further confirm the effectiveness of nonpharmacological interventions on these outcomes. Fourth, our meta-analysis did not include a subgroup analysis that examined various durations of follow-up. This omission was due to the scarcity of studies exploring long-term impacts, including those spanning over a period of 6 months, which limits our ability to explore the follow-up benefits of nonpharmacological interventions for these groups of parents. Therefore, large-scale RCTs of nonpharmacological interventions with long-term follow-up for both intervention and control groups are needed to investigate the long-term benefits. Furthermore, the majority of included studies were conducted in Western or developed countries. However, considering the high proportion of perinatal loss occurring in low- and middle-income countries and rural areas, future research and practice are needed to examine the effectiveness of nonpharmacological interventions for those underprivileged parents. Last but not least, considering the encouraging results regarding different outcomes and the identification of the optimal elements of nonpharmacological therapies, it is recommended that future research investigates and assesses these findings/components, with the aim of developing interventions that are both user-friendly and cost-effective for clients.

## 5. Conclusion

The results of this review provide support for the effectiveness of nonpharmacological interventions in improving parental grief, PTSD, depression, anxiety, and perceived social support in parents who have experienced perinatal loss. The subgroup analysis identified that cognitive-based interventions delivered in a face-to-face, individual-based format with a maximum of four sessions were most beneficial. Additional well-designed RCTs with larger sample sizes and longer-term follow-up are necessary to validate these positive findings and investigate the mechanisms through which nonpharmacological interventions effectively enhance health outcomes for parents with a wider range of sociodemographic and ethnic backgrounds.

## Figures and Tables

**Table 1:** Eligibility criteria of the included studies.

PICO	Inclusion criteria	Exclusion criteria
Participants	(i) Studies involving parents (both father and mother or either parent) who had lost a child due to miscarriage, stillbirth, neonatal death, ectopic pregnancy, or termination of pregnancy (TOP) due to detection of fetal health problems or fetal abnormality (ii) Studies involving parents who were in the postpartum period of less than 1 year	(i) Studies including parents who were pregnant and intending to continue the pregnancy (ii) Studies including parents whose child died beyond the neonatal phase (first 28 days of life)

Intervention	(i) All nonpharmacological interventions	(i) Studies assessing the efficacy of pharmacological interventions

Comparators	(i) Waitlisted or no treatment (ii) Usual/standard care or attention control (iii) Alternative treatment (other nonpharmacological interventions) (iv) Placebo control	(i) Studies that did not have any control or comparison groups

Outcomes	(i) Studies reporting at least one parental mental health (e.g., grief, stress, depression, anxiety, and distress) and/or well-being (e.g., sleep quality and perceived social support) outcome(s) measured with valid instruments at least postintervention	(i) Studies that did not include any targeted parent outcomes (ii) Attrition rate over 50%

Study design	(i) Pilot studies with parallel-group and random allocation (ii) Randomized controlled trials (RCTs), including cluster RCTs, with two or more than two arms (iii) Studies published in English (iv) Published between January 2008 and December 2023 (~15 years)	(i) Non-RCTs, single-group comparisons, pretest and posttest studies (ii) Protocols, reviews, conference abstracts, books, and case reports (iii) Published before 2008

**Table 2:** Summary of findings.

Outcomes	Meta-analyses			Narrative syntheses			Certainty of the evidence
	No. of participants (no. of RCTs)	SMD (Hedges' *g*)	References	No. of participants	References	Cohen's *d* (95% CI)	
Parental grief	976 (10)	**−0.68 (95% CI: −0.98, −0.38; *p*-value for heterogeneity chi-squared test <0.01, *I*** ^**2**^ **= 79%)**	[6, 20, 21, 32, 33, 34, 35, 37, 45, 47, 48, 49]	50 682	[36, 38]	−0.33 (−0.03, −0.62) Not report, significantly improvement	Moderate^a^

Parental stress	388 (5)	−1.34 (95% CI: −2.75, 0.07; *p* < 0.01, *I*^2^ = 96%)	[18, 19, 39, 40, 44]	—	—	—	Low^a,b^

Parental PTSD	532 (5)	**−0.69 (95% CI: −0.92, −0.46; **p**=0.21, *I*** ^**2**^ **= 31%)**	[6, 21, 42, 45, 46]	—	—	—	High

Parental depression	1,521 (15)	**−0.63 (95% CI: −0.95, −0.30; **p** < 0.01, *I*** ^**2**^ **= 87%)**	[6, 14, 18, 20, 21, 37, 39, 40, 41, 42, 44, 45, 46, 47, 48]	50 862	[36, 38]	−0.04 (−0.32, 0.24) Not report, significantly improvement	Moderate^a^

Parental anxiety	960 (10)	**−0.72 (95% CI: −1.19, −0.24; **p** < 0.01, *I*** ^***2***^ **= 89%)**	[6, 18, 20, 21, 44, 45, 46, 47, 48, 49]	—	—	—	Moderate^a^

Parental distress	474 (3)	−1.89 (95% CI: −4.08, 0.31; *p* < 0.01, *I*^2^ = 98%)	[18, 41, 44]	—	—	—	Moderate^a^

Parental sleep quality	214 (4)	0.19 (95% CI: −0.34, 0.73; *p*=0.01, *I*^2^ = 72%)	[21, 39, 40, 49]	—	—	—	Low^a,b^

Perceived social support	333 (5)	**0.31 (95% CI: 0.08, 0.55; **p**=0.31, *I*** ^***2***^ **= 16%)**	[14, 37, 39, 40, 49]	50	[36]	0.27 (−0.01, 0.56)	Moderate^b^

^a^Certainty rated down for statistical criteria for heterogeneity (Inconsistency; *I*^2^ >50% and chi-squared test *p*  < 0.05). ^b^Certainty rated down for limited number of participants (Imprecision; number of participants <400). The SMD values in bold indicate significant changes.

## Data Availability

The Supplementary Information files contain all of the data created or analyzed during the investigation.

## Supplementary Materials

Supplementary Materials All the additional results of data analysis, figures, and tables mentioned in this manuscript were displayed in the supplementary material.
