# Supplementary material for: Effectiveness of Nonpharmacological Interventions for Improving the Mental Health and Other Psychosocial Outcomes of Parents with Perinatal Loss: A Systematic Review and Meta-Analysis
Source: Depress Anxiety. 2024 Aug 28;2024:2181544. doi: 10.1155/2024/2181544 (PMC11919000; doi:10.1155/2024/2181544)
Supplement: Supplementary Materials — All the additional results of data analysis, figures, and tables mentioned in this manuscript were displayed in the supplementary material. [file 2181544.f1.docx]

**Supplementary Contents**

[Supplementary Table 1. PICO framework 5](#_Toc173927390)

[Supplementary Table 2. Research strategies for all databases 7](#_Toc173927391)

[Supplementary Table 3. Characteristics of the included studies 18](#_Toc173927392)

[Supplementary Table 4. Measurement instruments used in the non-pharmacological interventions 33](#_Toc173927393)

[Supplementary Figure 1. Flow Diagram for Searching and Selection of Articles 36](#_Toc173927394)

[Supplementary Figure 2. (a) Risk of bias graph about each risk of bias item presented as percentages across all included studies; (b) Summary of risk of bias for each study assessed by Cochrane Collaboration's tool. 37](#_Toc173927395)

[Supplementary Figure 3. Forest plots: effect of non-pharmacological interventions on (a) parental grief; (b) stress; (c) post-traumatic stress disorder; and (d) depression for parents with pregnancy loss. 38](#_Toc173927396)

[Supplementary Figure 4. Forest plots: effect of non-pharmacological interventions on (a) parental anxiety; (b) distress; (c) sleep quality; and (d) perceived social support for parents with pregnancy loss. 39](#_Toc173927397)

[Supplementary Figure 5. The funnel plot and Egger's test for the effects of the non-pharmacological interventions on grief for parents with perinatal loss. 40](#_Toc173927398)

[Supplementary Figure 6. The funnel plot and Egger's test for the effects of the non-pharmacological interventions on depression for parents with perinatal loss. 41](#_Toc173927399)

[Supplementary Figure 7. The funnel plot and Egger's test for the effects of the non-pharmacological interventions on anxiety for parents with perinatal loss. 42](#_Toc173927400)

[Supplementary Figure 8. Sensitivity analysis for the effects of the non-pharmacological interventions on parental grief for parents with perinatal loss. 43](#_Toc173927401)

[Supplementary Figure 9. Sensitivity analysis for the effects of the non-pharmacological interventions on parental stress for parents with perinatal loss. 43](#_Toc173927402)

[Supplementary Figure 10. Sensitivity analysis for the effects of the non-pharmacological interventions on parental post-traumatic stress disorder for parents with perinatal loss. 43](#_Toc173927403)

[Supplementary Figure 11. Sensitivity analysis for the effects of the non-pharmacological interventions on parental depression for parents with perinatal loss. 44](#_Toc173927404)

[Supplementary Figure 12. Sensitivity analysis for the effects of the non-pharmacological interventions on parental anxiety for parents with perinatal loss. 44](#_Toc173927405)

[Supplementary Figure 13. Sensitivity analysis for the effects of the non-pharmacological interventions on parental distress for parents with perinatal loss. 45](#_Toc173927406)

[Supplementary Figure 14. Sensitivity analysis for the effects of the non-pharmacological interventions on parental sleep quality for parents with perinatal loss. 45](#_Toc173927407)

[Supplementary Figure 15. Sensitivity analysis for the effects of the non-pharmacological interventions on parental perceived social support for parents with perinatal loss. 45](#_Toc173927408)

[Explanation for the Results of Sensitivity Analysis 46](#_Toc173927409)

[Supplementary Figure 16. Subgroup analysis to compare the effects of different intervention types of the non-pharmacological interventions on parental grief for parents with perinatal loss. 47](#_Toc173927410)

[Supplementary Figure 17. Subgroup analysis to compare the effects of different intervention types of the non-pharmacological interventions on parental stress for parents with perinatal loss. 47](#_Toc173927411)

[Supplementary Figure 18. Subgroup analysis to compare the effects of different intervention types of the non-pharmacological interventions on parental post-traumatic stress disorder for parents with perinatal loss. 48](#_Toc173927412)

[Supplementary Figure 19. Subgroup analysis to compare the effects of different intervention types of the non-pharmacological interventions on parental depression for parents with perinatal loss. 48](#_Toc173927413)

[Supplementary Figure 20. Subgroup analysis to compare the effects of different intervention types of the non-pharmacological interventions on parental anxiety for parents with perinatal loss. 49](#_Toc173927414)

[Supplementary Figure 21. Subgroup analysis to compare the effects of different intervention types of the non-pharmacological interventions on perceived social support for parents with perinatal loss. 49](#_Toc173927415)

[Supplementary Table 5. Comparison of treatment effects among different intervention types of the non-pharmacological interventions for parents with perinatal loss. 50](#_Toc173927416)

[Supplementary Figure 22. Subgroup analysis to compare the effects of different delivery modalities of the non-pharmacological interventions on parental grief for parents with perinatal loss. 51](#_Toc173927417)

[Supplementary Figure 23. Subgroup analysis to compare the effects of different delivery modalities of the non-pharmacological interventions on parental stress for parents with perinatal loss. 51](#_Toc173927418)

[Supplementary Figure 24. Subgroup analysis to compare the effects of different delivery modalities of the non-pharmacological interventions on parental post-traumatic stress disorder for parents with perinatal loss. 52](#_Toc173927419)

[Supplementary Figure 25. Subgroup analysis to compare the effects of different delivery modalities of the non-pharmacological interventions on parental depression for parents with perinatal loss. 52](#_Toc173927420)

[Supplementary Figure 26. Subgroup analysis to compare the effects of different delivery modalities of the non-pharmacological interventions on parental anxiety for parents with perinatal loss. 53](#_Toc173927421)

[Supplementary Figure 27. Subgroup analysis to compare the effects of different delivery modalities of the non-pharmacological interventions on parental distress for parents with perinatal loss. 53](#_Toc173927422)

[Supplementary Figure 28. Subgroup analysis to compare the effects of different delivery modalities of the non-pharmacological interventions on perceived social support for parents with perinatal loss. 54](#_Toc173927423)

[Supplementary Table 6. Comparison of treatment effects among different delivery modalities of the non-pharmacological interventions for parents with perinatal loss. 54](#_Toc173927424)

[Supplementary Figure 29. Subgroup analysis to compare the effects of different delivery formats of the non-pharmacological interventions on parental grief for parents with perinatal loss. 55](#_Toc173927425)

[Supplementary Figure 30. Subgroup analysis to compare the effects of different delivery formats of the non-pharmacological interventions on parental stress for parents with perinatal loss. 55](#_Toc173927426)

[Supplementary Figure 31. Subgroup analysis to compare the effects of different delivery formats of the non-pharmacological interventions on parental post-traumatic stress disorder for parents with perinatal loss. 56](#_Toc173927427)

[Supplementary Figure 32. Subgroup analysis to compare the effects of different delivery formats of the non-pharmacological interventions on parental depression for parents with perinatal loss. 56](#_Toc173927428)

[Supplementary Figure 33. Subgroup analysis to compare the effects of different delivery formats of the non-pharmacological interventions on parental anxiety for parents with perinatal loss. 57](#_Toc173927429)

[Supplementary Figure 34. Subgroup analysis to compare the effects of different delivery formats of the non-pharmacological interventions on perceived social support for parents with perinatal loss. 57](#_Toc173927430)

[Supplementary Table 7. Comparison of treatment effects among different delivery formats of the non-pharmacological interventions for parents with perinatal loss. 58](#_Toc173927431)

[Supplementary Figure 35. Subgroup analysis to compare the effects of different types of perinatal loss of the non-pharmacological interventions on parental grief for parents with perinatal loss. 59](#_Toc173927432)

[Supplementary Figure 36. Subgroup analysis for the effects of different types of perinatal loss the non-pharmacological interventions on parental stress for parents with perinatal loss. 59](#_Toc173927433)

[Supplementary Figure 37. Subgroup analysis for the effects of different types of perinatal loss the non-pharmacological interventions on parental post-traumatic stress disorder for parents with perinatal loss. 60](#_Toc173927434)

[Supplementary Figure 38. Subgroup analysis for the effects of different types of perinatal loss the non-pharmacological interventions on parental depression for parents with perinatal loss. 60](#_Toc173927435)

[Supplementary Figure 39. Subgroup analysis for the effects of different types of perinatal loss the non-pharmacological interventions on parental anxiety for parents with perinatal loss. 61](#_Toc173927436)

[Supplementary Table 8. Comparison of treatment effects among different types of perinatal loss of the non-pharmacological interventions for parents with perinatal loss. 62](#_Toc173927437)

[Supplementary Figure 40. Subgroup analysis for the effects of different intervention sessions the non-pharmacological interventions on parental grief for parents with perinatal loss. 63](#_Toc173927438)

[Supplementary Figure 41. Subgroup analysis for the effects of different intervention sessions the non-pharmacological interventions on parental stress for parents with perinatal loss. 63](#_Toc173927439)

[Supplementary Figure 42. Subgroup analysis for the effects of different intervention sessions the non-pharmacological interventions on parental post-traumatic stress disorder for parents with perinatal loss. 64](#_Toc173927440)

[Supplementary Figure 43. Subgroup analysis for the effects of different intervention sessions the non-pharmacological interventions on parental depression for parents with perinatal loss. 64](#_Toc173927441)

[Supplementary Figure 44. Subgroup analysis for the effects of different intervention sessions the non-pharmacological interventions on parental anxiety for parents with perinatal loss. 65](#_Toc173927442)

[Supplementary Figure 45. Subgroup analysis for the effects of different intervention sessions the non-pharmacological interventions on perceived social support for parents with perinatal loss. 65](#_Toc173927443)

[Supplementary Table 9. Comparison of treatment effects among different intervention sessions of the non-pharmacological interventions for parents with perinatal loss. 66](#_Toc173927444)

[Reference 67](#_Toc173927445)

# Supplementary Table 1. PICO framework

| Population | Parents with perinatal loss |
| --- | --- |
| Interventions | All non-pharmacological interventions  Psychotherapies   - Cognitive behavioral therapy (CBT) - Dialectical behavioral therapy (DBT) - Acceptance and Commitment therapy (ACT) - Relaxation - Skills training - Guided self-help - Mindfulness-based cognitive therapy - Mindfulness (other than mindfulness-based cognitive therapy) - Psychosocial interventions - Psychoeducation - Behavioral activation - Counselling - Arts/creative psychotherapy   - Art therapy   - Psychodrama   - Music therapy   - Dance therapy |
| Comparator | **Active comparator**:  Any alternative interventions  Attention care  **Inactive comparator**:  Usual care/standard care  Waiting list  No intervention |
| Outcomes | Not limited |
| Studies | Randomized controlled trials (RCTs)  Pilots RCT with parallel-group and random allocation |

# Supplementary Table 2. Research strategies for all databases

**Table 1a. Research strategy – PubMed (19 Dec 2023)**

| #ID | Topic or intervention | Query | Records |
| --- | --- | --- | --- |
| #1 | Population - parents | (parents[Mesh]) OR (parent*[Title/Abstract] OR mother*[Title/Abstract] OR wom?n[Title/Abstract] OR father*[Title/Abstract] OR maternal*[Title/Abstract] OR caregiver*[Title/Abstract]) OR (parental[Title/Abstract]) | 1,050024 |
| #2 | Population - perinatal loss | (Abortion, Spontaneous[Mesh]) OR (perinatal death[Mesh]) OR (Fetal Death[Mesh]) OR (spontaneous abortion*[Title/Abstract] OR pregnancy loss[Title/Abstract] OR reproductive loss[Title/Abstract] OR miscarriage*[ Title/Abstract] OR perinatal death*[ Title/Abstract] OR perinatal loss[Title/Abstract] OR perinatal grief[Title/Abstract] OR stillbirth[Title/Abstract] OR Fetal Resorption[Title/Abstract] OR fetal death[Title/Abstract] OR foetal death[Title/Abstract] OR infant death[Title/Abstract]) | 105,962 |
| #3 | Intervention – Non-pharmacological interventions | psychotherapy[Mesh] OR (psychotherapy*) OR (psychological) OR (psychosocial) OR (cognitive behavioral) OR (CBT) OR (Dialectical behavioral therapy) OR (Acceptance and Commitment) OR (ACT) OR (mindfulness*) OR (mindful*) OR (psychoeducation) OR (relaxation) OR (Guided self-help) OR (conselling) OR (Positive psychology) OR (Social support) OR (Writing disclose*) OR (Behavioral activation) OR (Art therapy) OR (psychodrama) OR (music therapy) OR (dance therapy) OR (bereavement care) | 3,096,938 |
| #4 | Study type - RCT | (Randomized Controlled Trial [Publication Type]) OR (Controlled Clinical Trial[Publication Type]) OR (Randomized Clinical Trials as Topic[Mesh]) OR (Clinical Trials as Topic[Mesh]) OR Randomi*[Title/Abstract] OR (Random Allocation[Title/Abstract]) OR (Clinical Trial[Title/Abstract]) OR (Double-Blind Method[Title/Abstract]) OR (Single-Blind Method[Title/Abstract]) NOT (protocol*[Title/Abstract]) | 1,339,096 |
| #5 | Results | #1 AND #2 AND #3 AND #4 | 162 |

**Table 2b. Research strategy – Embase (20 Dec 2023)**

| #ID | Topic or intervention | Query | Records |
| --- | --- | --- | --- |
| #1 | Population - parents | exp parent/ | 289963 |
| #2 |  | (parent* or mother* or wom?n or father* or maternal* or caregiver*or parental).tw. | 12984452 |
| #3 |  | #1 or #2 | 12985041 |
| #4 | Population - perinatal loss | exp abortion / or exp perinatal death / or exp fetus death / | 153323 |
| #5 |  | (spontaneous abortion* OR pregnancy loss OR reproductive loss OR miscarriage* OR perinatal death* OR perinatal loss OR perinatal grief OR stillbirth OR fetal resorption OR fetal death OR foetal death OR infant death) .tw. | 144650 |
| #6 |  | #4 or #5 | 188812 |
| #7 | Intervention - Non-pharmacological interventions | exp psychotherapy / | 319796 |
| #8 |  | (psychotherapy* OR psychological OR psychosocial OR cognitive behavioral OR CBT OR Dialectical behavioral therapy OR Acceptance and Commitment OR ACT OR mindfulness* OR mindful* OR psychoeducation OR relaxation OR Guided self-help OR conselling OR Positive psychology OR Social support OR Writing disclose* OR Behavioral activation OR Art therapy OR psychodrama OR music therapy OR dance therapy OR bereavement care) .tw. | 2264105 |
| #9 |  | #7 or #8 | 2347425 |
| #10 | Study type- RCT | exp Randomized Controlled Trial/ or exp Controlled Clinical Trial/ or exp Clinical Trials as Topic/ | 1410511 |
| #11 |  | (Randomi* or Random Allocation or Clinical Trial or Double-Blind or Single-Blind or Placebo* or Cross-Over Stud*).tw. | 3491123 |
| #12 |  | #10 or #11 | 3505353 |
| #13 | Results | #3 and #6 and #9 and #12 | 1295 |

**Table 3c. Research strategy – PsycINFO (23 Dec 2023)**

| #ID | Topic or intervention | Query | Records |
| --- | --- | --- | --- |
| #1 | Population - parents | MAINSUBJECT.EXACT.EXPLODE("Parents") OR tiab(parent* or mother* or wom?n or father* or maternal* or caregiver*or parental) | 698519 |
| #2 | Population - perinatal loss | MAINSUBJECT.EXACT.EXPLODE("Absorption (Physiological)") OR tiab(perinatal death OR fetus death OR spontaneous abortion* OR pregnancy loss OR reproductive loss OR miscarriage* OR perinatal death* OR perinatal loss OR perinatal grief OR stillbirth OR fetal resorption OR fetal death OR foetal death OR infant death) | 8688 |
| #3 | Intervention - Non-pharmacological interventions | (MAINSUBJECT.EXACT("Clinical Methods Training") OR MAINSUBJECT.EXACT.EXPLODE("Training")) OR tiab(psychotherapy* OR psychological OR psychosocial OR cognitive behavioral OR CBT OR Dialectical behavioral therapy OR Acceptance and Commitment OR ACT OR mindfulness* OR mindful* OR psychoeducation OR relaxation OR Guided self-help OR conselling OR Positive psychology OR Social support OR Writing disclose* OR Behavioral activation OR Art therapy OR psychodrama OR music therapy OR dance therapy OR bereavement care) | 910074 |
| #4 | Study type- RCT | (MAINSUBJECT.EXACT.EXPLODE("Randomized Clinical Trials") OR MAINSUBJECT.EXACT.EXPLODE("Randomized Controlled Trials")) OR MAINSUBJECT.EXACT.EXPLODE("Clinical Trials") OR tiab(Controlled Clinical Trial or Randomi* or Random Allocation or Clinical Trial or Double-Blind or Single-Blind or Placebo* or Cross-Over Stud*) | 169070 |
| #5 | Results | #1 and #2 and #3 and #4 | 118 |

**Table 4d. Research strategy – CINAHL (23 Dec 2023)**

| #ID | Topic or intervention | Query | Records |
| --- | --- | --- | --- |
| S1 | Population - parents | (MH “parents+”) | 117579 |
| S2 |  | TI (parent* or mother* or father* or maternal* or caregiver* or parental) OR AB (parent* or mother* or father* or maternal* or caregiver* or parental) | 373022 |
| S3 |  | S1 OR S2 | 405168 |
| S4 | Population - perinatal loss | (MH “abortion, spontaneous+”) OR (MH “perinatal death+”) | 15660 |
| S5 |  | TI(spontaneous abortion* OR pregnancy loss OR reproductive loss OR miscarriage* OR perinatal death* OR perinatal loss OR perinatal grief OR stillbirth OR fetal resorption OR fetal death OR foetal death OR infant death) OR AB(spontaneous abortion* OR pregnancy loss OR reproductive loss OR miscarriage* OR perinatal death* OR perinatal loss OR perinatal grief OR stillbirth OR fetal resorption OR fetal death OR foetal death OR infant death) | 22109 |
| S6 |  | S4 OR S5 | 28871 |
| S7 | Intervention - Non-pharmacological interventions | (MH "Psychosocial Intervention") OR (MH "Psychotherapy+") OR TI ( (psychotherapy* OR psychological OR psychosocial OR cognitive behavioral OR CBT OR Dialectical behavioral therapy OR Acceptance and Commitment OR ACT OR mindfulness* OR mindful* OR psychoeducation OR relaxation OR Guided self-help OR conselling OR Positive psychology OR Social support OR Writing disclose* OR Behavioral activation OR Art therapy OR psychodrama OR music therapy OR dance therapy OR bereavement care ) OR AB ( (psychotherapy* OR psychological OR psychosocial OR cognitive behavioral OR CBT OR Dialectical behavioral therapy OR Acceptance and Commitment OR ACT OR mindfulness* OR mindful* OR psychoeducation OR relaxation OR Guided self-help OR conselling OR Positive psychology OR Social support OR Writing disclose* OR Behavioral activation OR Art therapy OR psychodrama OR music therapy OR dance therapy OR bereavement care ) | 497619 |
| S8 | Study type – RCT | (MH "Randomized Controlled Trials+") OR (MH "Clinical Trials+") | 351387 |
| S9 |  | TI (Randomi* or Random Allocation or Clinical Trial or Double-Blind or Single-Blind or Placebo* or Cross-Over Stud*) OR AB (Randomi* or Random Allocation or Clinical Trial or Double-Blind or Single-Blind or Placebo* or Cross-Over Stud*) | 422999 |
| S10 |  | S8 OR S9 | 567474 |
| S11 | Results | S3 AND S6 AND S7 AND S10 | 78 |

**Table 5e. Research strategy – ProQuest (24 Dec 2023)**

| #ID | Topic or intervention | Query | Records |
| --- | --- | --- | --- |
| S1 | Population - parents | title(parent* or mother* or father* or maternal* or caregiver* or parental) OR abstract(parent* or mother* or father* or maternal* or caregiver* or parental) | 731661 |
| S2 | Population - perinatal loss | title('spontaneous abortion*' OR 'pregnancy loss' OR 'reproductive loss' OR 'miscarriage*' OR 'perinatal death*' OR 'perinatal loss' OR 'perinatal grief' OR 'stillbirth' OR 'fetal resorption' OR 'fetal death' OR 'foetal death' OR 'infant death') OR abstract('spontaneous abortion*' OR 'pregnancy loss' OR 'reproductive loss' OR 'miscarriage*' OR 'perinatal death*' OR 'perinatal loss' OR 'perinatal grief' OR 'stillbirth' OR 'fetal resorption' OR 'fetal death' OR 'foetal death' OR 'infant death') | 56758 |
| S3 | Intervention - Non-pharmacological interventions | title('Psychosocial Intervention' OR 'Psychotherapy' OR psychotherapy* OR psychological OR psychosocial OR 'cognitive behavioral' OR CBT OR 'Dialectical behavioral therapy' OR 'Acceptance and Commitment' OR ACT OR mindfulness* OR mindful* OR psychoeducation OR relaxation OR 'Guided self-help' OR conselling OR 'Positive psychology' OR 'Social support' OR 'Writing disclose*' OR 'Behavioral activation' OR 'Art therapy' OR psychodrama OR 'music therapy' OR 'dance therapy' OR 'bereavement care') OR abstract('Psychosocial Intervention' OR 'Psychotherapy' OR psychotherapy* OR psychological OR psychosocial OR 'cognitive behavioral' OR CBT OR 'Dialectical behavioral therapy' OR 'Acceptance and Commitment' OR ACT OR mindfulness* OR mindful* OR psychoeducation OR relaxation OR 'Guided self-help' OR conselling OR 'Positive psychology' OR 'Social support' OR 'Writing disclose*' OR 'Behavioral activation' OR 'Art therapy' OR psychodrama OR 'music therapy' OR 'dance therapy' OR 'bereavement care') | 1283903 |
| S4 | Intervention - Non-pharmacological interventions | title('Randomized Controlled Trial*' or Randomi* or 'Random Allocation' or 'Clinical Trial' or 'Double-Blind' or 'Single-Blind' or Placebo* or 'Cross-Over Stud*') OR abstract('Randomized Controlled Trial*' or Randomi* or 'Random Allocation' or 'Clinical Trial' or 'Double-Blind' or 'Single-Blind' or Placebo* or 'Cross-Over Stud*') | 687403 |
| S5 | Results | S1 AND S2 AND S3 AND S4 | 116 |

**Table 6f. Research strategy – Cochrane Central Register (24 Dec 2023)**

| #ID | Topic or intervention | Query | Records |
| --- | --- | --- | --- |
| #1 | Population - Caregivers | MeSH descriptor: [Parents] explode all trees | 7542 |
| #2 |  | (parent* or mother* or father* or maternal* or caregiver* or parental):ti,ab,kw | 105432 |
| #3 |  | #1 OR #2 | 105432 |
| #4 | Population - Children | MeSH descriptor: [Abortion, Spontaneous] explode all trees | 1218 |
| #5 |  | MeSH descriptor: [perinatal death] explode all trees | 165 |
| #6 |  | MeSH descriptor: [Fetal Death] explode all trees | 546 |
| #7 |  | (spontaneous abortion* OR pregnancy loss OR reproductive loss OR miscarriage* OR perinatal death* OR perinatal loss OR perinatal grief OR stillbirth OR fetal resorption OR fetal death OR foetal death OR infant death):ti,ab,kw | 16107 |
| #8 |  | #4 or #5 or #6 or #7 | 16314 |
| #9 | Intervention - ACT | MeSH descriptor: [Psychotherapy] explode all trees | 33651 |
| #10 |  | (psychotherapy* OR psychological OR psychosocial OR cognitive behavioral OR CBT OR Dialectical behavioral therapy OR Acceptance and Commitment OR ACT OR mindfulness* OR mindful* OR psychoeducation OR relaxation OR Guided self-help OR conselling OR Positive psychology OR Social support OR Writing disclose* OR Behavioral activation OR Art therapy OR psychodrama OR music therapy OR dance therapy OR bereavement care):ti,ab,kw | 258196 |
| #11 |  | #9 OR #10 | 261575 |
| #12 | Study type – RCT | MeSH descriptor: [Randomized Controlled Trial] explode all trees | 25732 |
| #13 |  | MeSH descriptor: [Controlled Clinical Trial] explode all trees | 38477 |
| #14 |  | MeSH descriptor: [Clinical Trials as Topic] explode all trees | 84414 |
| #15 |  | (Randomi* or Random Allocation or Clinical Trial or Double-Blind or Single-Blind or Placebo* or Cross-Over Stud*):ti,ab,kw | 1393551 |
| #16 |  | #12 or #13 or #14 or #15 | 1393692 |
| #17 | Results | #3 AND #8 AND #11 AND #16 | 595 |

**Table 7g. Research strategy –** **Scopus (24 Dec 2023)**

| #ID | Topic or intervention | Query | Records |
| --- | --- | --- | --- |
| #1 | Population - parents | TITLE-ABS-KEY (parent* or mother* or father* or maternal* or caregiver* or parental) | 1865387 |
| #2 | Population - perinatal loss | TITLE-ABS-KEY ( ( spontaneous AND abortion* ) OR ( pregnancy AND loss ) OR ( reproductive AND loss ) OR miscarriage* OR ( perinatal AND death* ) OR ( perinatal AND loss ) OR ( perinatal AND grief ) OR stillbirth OR ( fetal AND resorption ) OR ( fetal AND death ) OR ( foetal AND death ) OR ( infant AND death ) ) | 287807 |
| #3 | Intervention - Non-pharmacological interventions | TITLE-ABS-KEY ( psychotherapy* OR psychological OR psychosocial OR ( cognitive AND behavioral ) OR cbt OR ( dialectical AND behavioral AND therapy ) OR ( acceptance AND commitment ) OR act OR mindfulness* OR mindful* OR psychoeducation OR relaxation OR ( guided AND self-help ) OR conselling OR ( positive AND psychology ) OR ( social AND support ) OR ( writing AND disclose* ) OR ( behavioral AND activation ) OR ( art AND therapy OR psychodrama ) OR ( music AND therapy ) OR ( dance AND therapy ) OR ( bereavement AND care ) ) | 3845779 |
| #4 | Intervention - Non-pharmacological interventions | TITLE-ABS-KEY ( ( randomized AND controlled AND trials ) OR ( clinical AND trials ) OR randomi* OR ( random AND allocation ) OR ( clinical AND trial ) OR ( double-blind ) OR ( single-blind ) OR placebo* OR ( cross-over AND stud* ) ) | 2913928 |
| #5 | Results | #1 and #2 and #3 and #4 | 706 |

# Supplementary Table 3. Characteristics of the included studies

| **Study ID, location** | **Design;**  **Categories (Number of participants)** | **Women characteristics; Types of perinatal loss; Age: mean (SD)** | **Description of intervention (recipient, content, modality, format, intensity, time point of data collection, retention rate, setting, provider, and theoretical framework)** | **Outcomes (instruments)** |
| --- | --- | --- | --- | --- |
| (Barat et al., 2020), Iran | 2-arm RCT; **Tx:** Supportive intervention (39)  **Ct:** Placebo control + Standard medical care (40) | Miscarriage  Mean age: 18.29 (8.87) | - **Recipient:** Mothers - **Content:**   - **Tx:** The common elements of supportive intervention approach for treatment include making an emotional connection, following the affect, letting it linger, encouraging catharsis, building alliance, and emphasizing the patient’s strengths (but not avoiding negative affect).   - **Ct:** two counseling sessions - **Modality:** Face-to-face - **Format**: Individual-based - **Intensity**: 1-session, 2h per session - **Time point of data collection**: pre-test, posttest (4-month post-intervention) - **Retention rate**: post-intervention: 94.94%; follow-up: None. - **Setting:** Hospital - **Provider(s)**: Psychologist - **Theoretical framework:** NA | - Grief: Perinatal Grief Scale (PGS) - Depression: Hospital Anxiety and Depression Scale (HADS) - Anxiety: Hospital Anxiety and Depression Scale (HADS) |
| (Chang et al., 2021), China | 2-arm RCT; **Tx:** Counseling (31)  **Ct:** Routine care (31) | Recurrent miscarriage  Mean age: 34.71 (4.58) | - **Recipient:** Mothers - **Content:**   - **Tx:** The theoretical basis of the intervention is mainly empathy and caring with the objectives of discussing stressful events and emotions, and of constructing a safe environment and a positive emotional atmosphere in an interactive social context. The counseling principles of empathic caring were designed to facilitate self-awareness, reduce self-blame, clarify doubts, listen to patient concerns, promote positive thinking, and encourage life planning.   - **Ct:** Routine care - **Modality:** Face-to-face - **Format**: Individual-based - **Intensity**: 3-session, 12-week, NA, monthly interval - **Time point of data collection**: pre-test, posttest - **Retention rate**: post-intervention: 95.55%; follow-up: None. - **Setting:** Hospital - **Provider(s)**: Midwife - **Theoretical framework:** NA | - Stress: Perceived Stress Scale (PSS) - Depression: Edinburgh Prenatal Depression Scale (EPDS) - Sleep quality: The Pittsburgh Sleep Quality Index (PSQI) - Perceived social support: Interpersonal Support Evaluation List (ISEL) Short Form |
| (Haghighi et al., 2022), Iran | 2-arm RCT; **Tx:** Counseling (50)  **Ct:** Routine care (50) | Miscarriage  Mean age: 28.94 (5.84) | - **Recipient:** Mothers - **Content:**   - **Tx:** 1) improving the psychological aspects of pregnancy loss; and 2) helping bereaved mothers cope with their grief.   - **Ct:** Routine care - **Modality:** Face-to-face - **Format**: Individual-based - **Intensity**: 4-sessionn, 4-week, 1h per session, weekly interval - **Time point of data collection**: pre-test, posttest - **Retention rate**: post-intervention: NA; follow-up: None. - **Setting:** Hospital - **Provider(s)**: Midwifery consultant - **Theoretical framework:** Worden’s principles of grief | - Stress: The Depression, Anxiety and Stress Scale - 42 Items – stress subscale - Depression: The Depression, Anxiety and Stress Scale - 42 Items – depression subscale - Anxiety: The Depression, Anxiety and Stress Scale - 42 Items – anxiety subscale - Distress: The Depression, Anxiety and Stress Scale - 42 Items |
| (Huberty et al., 2020), USA | 3-arm pilot RCT; **Tx:** Mind-Body intervention (moderate doseage:30; low dosage: 30)  **Ct:** Stretch and tone control (30) | Stillbirth  Mean age: NA | - **Recipient:** Mothers - **Content:**   - **Tx:** mindfulness, Yoga   - **Ct:** Participants in the stretch and tone control (STC) group were asked to follow a 12-week online stretching/toning exercise prescription for 60 min per week to match the low doseage intervention group. The research team developed 12, 30-min videos for the STC group prescription (produced and filmed by Udaya). - **Modality:** eHealth (online platform) - **Format**: Individual-based - **Intensity**: 12-session, 12-week, NA, weekly interval - **Time point of data collection**: pre-test, posttest, 5-month follow-up - **Retention rate**: post-intervention: 53.33%; follow-up: None. - **Setting:** No limitation - **Provider(s)**: Yoga teachers - **Theoretical framework:** NA | - Grief: Perinatal Grief Scale (PGS) - Post-traumatic stress disorder: Impact of Event Scale (IES-R) - Depression: Patient Health Questionnaire-9 (PHQ-9) - Anxiety: State-Trait Anxiety Inventory (STAI) - Sleep quality: Pittsburgh Sleep Quality Index (PSQI) |
| (Hung et al., 2023), China | 2-arm pilot RCT; **Tx:** Mind-Body intervention (31)  **Ct:** Routine care (31) | Recurrent miscarriage  Mean age: 34.5 (5.08) | - **Recipient:** Mothers - **Content:**   - **Tx:** Six cmponents education PowerPoint presentations: (1) Health education PowerPoint presentations: Understanding Recurrent Miscarriage, and Psychological Adjustment; (2) Health education leaflets: A series of eight RM-related health education leaflets was developed. Each respectively addressed the role of vitamins in embryonic development, the role of thrombophilia in RM, the role of premature ovarian failure in RM, the effect of sperm on embryo quality, the role of chronic infections in RM, facing miscarriage with a positive attitude, strategies for improving sleep quality and the role of stress in miscarriage; (3) Audio-visual presentation on "experience sharing by people with a history of RM": a women and a couple with personal experience with RM were invited to share their thoughts and journeys; (4) Yoga exercise video: A 30-minute yoga exercise video was developed for the participants to use to reduce their stress and improve sleep quality; (5) hyperlinks to external websites such as those of community mental health services for additional information; and (6) Message board: An interactive online forum was designed to allow participants to message and interact with one another.   - **Ct:** Routine care - **Modality:** eHealth (online platform) - **Format**: Individual-based - **Intensity**: NA, 12-week, NA, NA - **Time point of data collection**: pre-test, posttest - **Retention rate**: post-intervention: 91.94%; follow-up: None. - **Setting:** No limitation - **Provider(s)**: Doctor specializing in habitual abortion and nursing lecturer with experience providing nursing counseling to women with recurrent miscarriage - **Theoretical framework:** NA | - Stress: Perceived Stress Scale (PSS) - Depression: Edinburgh Prenatal Depression Scale (EPDS) - Sleep quality: The Pittsburgh Sleep Quality Index (PSQI) - Perceived social support: Interpersonal Support Evaluation List (ISEL) Short Form |
| (Jensen et al., 2021), Denmark | 2-arm RCT; **Tx:** Cognitive-based intervention (38)  **Ct:**  Routine care (38) | Recurrent miscarriage  Mean age: 33.69 (5.26) | - **Recipient:** Mothers - **Content:**   - **Tx:** Breathing exercises, body scans, conscious movements, attention to experiences through the senses, and teaching in the origins of meditation.   - **Ct:** Routine care - **Modality:** Face-to-face - **Format**: Group-based - **Intensity**: 7-session, 7-week, ~11h for 7-session, weekly interval; one-booster - **Time point of data collection**: - **Retention rate**: post-intervention: 88.16%; follow-up: 84.21%. - **Setting:** Hospital - **Provider(s)**: Nurse - **Theoretical framework:** NA | - Stress: Perceived Stress Scale (PSS) |
| (J. E. Johnson et al., 2016), USA | 2-arm pilot RCT; **Tx:** Interpersonal psychotherapy (25)  **Ct:** Coping with Depression (25) | Mixed types  Mean age: 30.3 (6.6) | - **Recipient:** Mothers - **Content:**   - **Tx:** Each woman had the opportunity to participate in groups covering the four topics (the emotions of grief, understanding what happened, grieving with others, and holding the memory and moving forward; see Table 1) three times: once to begin work on each issue, once to assess progress and problem-solve any difficulties that arise, and then a final time to address any remaining issues and solidify gains.   - **Ct:** The coping with depression course is a highly structured, manualized psychoeducational group treatment for MDD. - **Modality:** Face-to-face - **Format**: Blended - **Intensity**: 14-session, NA, NA, NA - **Time point of data collection**: - **Retention rate**: post-intervention: 64%; follow-up: 58%. - **Setting:** No limitation - **Provider(s)**: Nurse and clinical psychology - **Theoretical framework:** NA | - Grief: Perinatal Bereavement Grief Scale (PBGS) - Depression: The modified Hamilton Rating Scale for Depression (HRSD) - Perceived social support: 12-item Multidimensional Scale for Perceived Social Support (MSPSS) - Distress: Dyadic Adjustment Scale (DAS) |
| (O. Johnson, 2009; O. P. Johnson & Langford, 2015), USA | 2-arm RCT; **Tx:** Supportive intervention (20)  **Ct:** Routine care (20) | Miscarriage  Mean age: 27.0 (7.0) | - **Recipient:** Mothers - **Content:**   - **Tx:** (a) early identification and labeling of the participant’s room and chart for acknowledgement of the loss; (b) offer of chaplain services or notification of the woman’s personal spiritual leader; (c) honor of any special request such as baptism, special ceremony, or prayer; (d) a packet of flower seeds of remembrance to be planted at home; (e) a soft plush care bear; (f) other physical mementos, if applicable; (g) participation in a naming ceremony; and (h) completion of a self-addressed sympathy card.   - **Ct:** - **Modality:** Blended (Face-to-face + phone call) - **Format**: Group-based - **Intensity**: 1-session, 2-week, 1h intervention and 15-minute telephone call, weekly interval - **Time point of data collection**: posttest (1-week post-intervention) - **Retention rate**: post-intervention: 100%; follow-up: 100%. - **Setting:** Hospital - **Provider(s)**: NA - **Theoretical framework:** NA | - Grief: Perinatal Grief Scale (PGS) |
| (A. Kersting et al., 2013), Germany | 2-arm RCT; **Tx:** Cognitive-based intervention (115)  **Ct:** Waitlist (113) | Mixed types  Mean age: 34.18 (5.15) | - **Recipient:** Parents (92% female) - **Content:**   - **Tx:** Writing assignments: (a) self-confrontation, (b) cognitive reappraisal, and (c) social sharing   - **Ct:** Waitlist + Cognitive-based intervention - **Modality:** eHealth (online platform) - **Format**: Individual-based - **Intensity**: 2-session, 5-week, 0.75 h per session, NA - **Time point of data collection**: pre-test, posttest, and 3-, 12-month follow-ups - **Retention rate**: post-intervention: 71.08%; follow-up: None. - **Setting:** No limitation - **Provider(s)**: Therapist - **Theoretical framework:** Cognitive behavioral therapy theory | - Grief: Inventory of Complicated Grief (ICG) - Post-traumatic stress disorder: German version of the Impact of Event Scale-Revised (IES-R) - Depression: The Brief Symptom Inventory (BSI) – Depression subscale - Anxiety: The Brief Symptom Inventory (BSI) – Axiety subscale |
| (Anette Kersting et al., 2011) , Germany | 2-arm pilot RCT; **Tx:** Cognitive-based intervention (48)  **Ct:** Waitlist (35) | Mixed types  Mean age: NA | - **Recipient:** Parents (92% female) - **Content:**   - **Tx:** Writing assignments: (a) self-confrontation, (b) cognitive reappraisal, and (c) social sharing   - **Ct:** Waitlist + Cognitive-based intervention - **Modality:** eHealth (online platform) - **Format**: Individual-based - **Intensity**: 2-session, 5-week, 0.75 h per session, NA - **Time point of data collection**: pre-test, posttest, and 3-, 12-month follow-ups - **Retention rate**: post-intervention: 71.08%; follow-up: None. - **Setting:** No limitation - **Provider(s)**: Therapist - **Theoretical framework:** Cognitive behavioral therapy theory | - Grief: Inventory of Complicated Grief (ICG) - Post-traumatic stress disorder: German version of the Impact of Event Scale-Revised (IES-R) - Depression: The Brief Symptom Inventory (BSI) – Depression subscale - Anxiety: The Brief Symptom Inventory (BSI) – Axiety subscale |
| (Kong et al., 2014), China | 2-arm RCT; **Tx:** Counseling (140)  **Ct:** Routine care (140) | Miscarriage  Mean age: NA | - **Recipient:** Mothers - **Content:**   - **Tx:** The basic elements used in the counselling program included listening, explaining and giving information on miscarriage, guidance and advice on further pregnancy, and encouraging hope.   - **Ct:** Routine care - **Modality:** Blended (Face-to-face + phone call) - **Format**: Individual-based - **Intensity**: 2-session, 2-week, 1h intervention + 0.5 h phone call, weekly interval - **Time point of data collection**: pre-test, posttest, and 3- and 6-month follow-up - **Retention rate**: post-intervention: 95.71%; follow-up: 76.43%. - **Setting:** Hospital - **Provider(s)**: Nurse - **Theoretical framework:** NA | - Depression: Beck Depression Inventory (BDI) - Distress: General Health Questionnaire (GHQ-12) |
| (Mecdi Kaydirak & Aslan, 2021), Turkey | 2-arm RCT; **Tx:** Supportive intervention (38)  **Ct:** Routine care (39) | Termination of pregnancy  Mean age: 30.67 (5.68) | - **Recipient:** Mothers - **Content:**   - **Tx:** The main goal of the TNSP is to make it easier for women to overcome mourning by increasing their adaptation to Roy’s compliance areas (physiologic, self-concept, role function, and mutual dependence).   - **Ct:** Routine care - **Modality:** Blended (Face-to-face + phone call) - **Format**: Individual-based - **Intensity**: 5-session, 5-week, Four 60-minute face-to-face interventions and one 20-minuite phone call, weekly interval - **Time point of data collection**: pre-test, posttest - **Retention rate**: post-intervention: 77.92%; follow-up: None. - **Setting:** Hospital - **Provider(s)**: Nurse - **Theoretical framework:** Roy adaptation model | - Grief: Perinatal Grief Scale (PGS) - Anxiety: State Anxiety Inventory-2 (STAI-2) score - Sleep quality: Visual Analog Scale - Perceived social support: The Scale of Ways of Coping with Stress (SWCS) - social support subscale |
| (Nasrollahi et al., 2022), Iran | 2-arm RCT; **Tx:** Cognitive-based intervention (53)  **Ct:** Routine care (53) | Miscarriage  Mean age: 29.12 (5.96) | - **Recipient:** Mothers - **Content:**   - **Tx:** Mindfulness‑based stress reduction: counseling, mindfulness, meditation, mindful yoga   - **Ct:** Routine care - **Modality:** Face-to-face - **Format**: Group-based - **Intensity**: 8-session, 8-week, 2h per session, weekly interval - **Time point of data collection**: pre-test, posttest - **Retention rate**: post-intervention: NA; follow-up: None. - **Setting:** Hospital - **Provider(s)**: Midwife - **Theoretical framework:** NA | - Stress: The Depression, Anxiety and Stress Scale - 21 Items – stress subscale - Depression: The Depression, Anxiety and Stress Scale - 21 Items – depression subscale - Anxiety: The Depression, Anxiety and Stress Scale - 21 Items – anxiety subscale - Distress: The Depression, Anxiety and Stress Scale - 21 Items |
| (Navidian & Saravani, 2018; Navidian et al., 2017), Iran | 2-arm RCT; **Tx:** Cognitive-based intervention (50)  **Ct:** Routine care (50) | Stillbirth  Mean age: 29.53 (6.49) | - **Recipient:** Mothers - **Content:**   - **Tx:** education, cognitive behavioral therapy   - **Ct:** Routine care - **Modality:** Face-to-face - **Format**: Group-based - **Intensity**: 4-session, 2-week, NA, weekly interval - **Time point of data collection**: pre-test, posttest - **Retention rate**: post-intervention: NA; follow-up: None. - **Setting:** Hospital - **Provider(s)**: Midwife - **Theoretical framework:** NA | - Grief: Perinatal Grief Scale (PGS) |
| (Rocha et al., 2018), Portugal | 2-arm RCT; **Tx:** Cognitive-based intervention (24)  **Ct:** Routine care (67) | Termination of pregnancy  Mean age: 33.04 (6.46) | - **Recipient:** Mothers - **Content:**   - **Tx:** Cognitive narrative therapy, including decision, subjectivation, metaphorization, and projecting   - **Ct:** Routine care - **Modality:** Face-to-face - **Format**: Individual-based - **Intensity**: 4-session, 4-week, 1h per session, weekly interval - **Time point of data collection**: pre-test, posttest (6-month post-intervention) - **Retention rate**: post-intervention: 80.22%; follow-up: None. - **Setting:** Hospital - **Provider(s)**: Psychologists - **Theoretical framework:** Ottawa decision framework/cognitive narrative model | - Grief: Perinatal Grief Scale (PGS) - Depression: Beck Depression Inventory (BDI) - Anxiety: Zung Anxiety Scale |
| (Rosenbaum et al., 2015), USA | 2-arm RCT; **Tx:** Supportive intervention (51)  **Ct:** Routine care (56) | Stillbirth  Mean age: NA | - **Recipient:** Parents (68.5% female) - **Content:**   - **Tx:** The 90-minute DVD (Grieving in the NICU – Mending Broken Hearts When a Baby Dies) is divided into 5 chapters to permit parental choice of viewing topic and timing of viewing. The DVD also includes a nationally recognized grief counselor’s and chaplain’s perspectives of the grief process.   - **Ct:** Routine care includes care by an interdisciplinary NICU team of neonatologists, nurses, social workers, chaplains, and patient-care associates, a CD created with photographs, a memory packet, locks of baby’s hair, plaster hand- and foot-prints, the gown in which the baby is dressed after death, a ring for the mother, and engraved necklace for the father.SBC also includes follow-up meetings, which usually occur 8-12 weeks after the baby’s death. - **Modality:** eHealth (DVD) - **Format**: Individual-based - **Intensity**: 90 minutes - **Time point of data collection**: follow-up (3 and 12 months after the baby’s death) - **Retention rate**: post-intervention: 92.52%; follow-up: 68.22%. - **Setting:** Hospital - **Provider(s)**: NICU team of neonatologists, nurses, social workers, chaplains, and patient-care associates - **Theoretical framework:** Betty Neuman's systems Model | - Grief: Perinatal Grief Scale (PGS) - Depression: Center for Epidemiologic Studies-Depression (CES-D) - Perceived social support: 24-item Social Provisions Scale (SPS) |
| (Séjourné et al., 2010), France | 2-arm RCT; **Tx:** Cognitive-based intervention (66)  **Ct:** Waitlist (68) | Termination of pregnancy  Mean age: 31.82（4.99） | - **Recipient:** Mothers - **Content:**   - **Tx:** support, psychoeducation, Cognitive behavioral therapy   - **Ct:** Waitlist + Cognitive-based intervention - **Modality:** Face-to-face - **Format**: Individual-based - **Intensity**: 1-session, NA, NA - **Time point of data collection**: 3 weeks, 10 weeks, 6 months post-miscarriage - **Retention rate**: post-intervention: NA; follow-up: 50%. - **Setting: S**emi-private clinics - **Provider(s)**: psychologist - **Theoretical framework:** NA | - Post-traumatic stress disorder: Impact of Event Scale-Revised (IES-R) - Depression: Hospital Anxiety Depression Scale - Depression subscale (HADS-D) - Anxiety: Hospital Anxiety Depression Scale - Anxiety subscale (HADS-A) |
| (Simpson et al., 2015), India | 2-arm RCT; **Tx:** Counseling (45)  **Ct:** Routine care (45) | Stillbirth  Mean age: NA | - **Recipient:** Mothers - **Content:**   - **Tx:** Counseling   - **Ct:** Routine care - **Modality:** Face-to-face - **Format**: Individual-based - **Intensity**: 3-session, 3-week, 0.5 to 0.75 hours, NA - **Time point of data collection**: pre-test, posttest (4-6 weeks post-discharge) - **Retention rate**: post-intervention: 100%; follow-up: None. - **Setting:** Hospital - **Provider(s)**: Nurse - **Theoretical framework:** NA | - Grief: Perinatal Grief Scale (PGS) - Depression: The Hamilton Depression Scale (HAM-D) - Anxiety: The Hamilton Anxiety Scale (HAM-A) |
| (Sun et al., 2018), China | 2-arm RCT; **Tx:** Counseling (62)  **Ct:** Routine care (62) | Termination of pregnancy  Mean age: NA | - **Recipient:** Mothers and other family members - **Content:**   - **Tx:** Information support package, education in family support, postpartum guidance, online real‐time guidance and communication, and follow‐up and consultation   - **Ct:** The medical staff provided graded psychological care according to the results of psychological assessment at the time of admission. - **Modality:** Blended (Face-to-face + online [WeChat public platform and WeChat group]) - **Format**: Individual-based - **Intensity**: NA, 6-Week, NA, NA - **Time point of data collection**: pre-test, posttest (6-week post-intervention) - **Retention rate**: post-intervention: 95.16%; follow-up: 64.52%. - **Setting:** Hospital - **Provider(s)**: Nurse - **Theoretical framework:** Psychological stress theory | - Post-traumatic stress disorder: The Chinese version of the Impact of Event Scale-Revised (IES-R) - Depression: Edinburgh Postnatal Depression Scale (EPDS) |
| (Swanson et al., 2009), USA | 4-arm RCT; **T1:** Combined caring (170), **T2:** Nurse caring (168), **T3:** Self-caring (172), **Ct:** Placebo (172) | Miscarriage  Mean age: NA | - **Recipient:** Parents (50% female) - **Content:**   - **Tx:** **T1:** Combined caring: one counseling session plus three self-caring modules, **T2:** Nurse caring: three counseling sessions, **T3:** Self-caring: three counseling sessions   - **Ct:** no treatment - **Modality:** Combined (T1), face-to-face (T2), videos (T3) - **Format**: Individual-based - **Intensity**: One counseling session + three self-care sessions (T1), three counseling session (T2), three counseling sessions (T3); NA, NA, Monthly - **Time point of data collection**: pre-test, and 3, 5, and 13 months after miscarriage. - **Retention rate**: post-intervention: 96.19%; follow-up: 85.34%. - **Setting:** Participants’ homes - **Provider(s)**: nurse counselors - **Theoretical framework:** Swanson’s Caring Theory and Meaning of Miscarriage Model | - Grief: Miscarriage Grief Inventory (MGI) - Depression: Center for Epidemiological Studies-Depression scale (CES-D) |
| (Wang et al., 2023), China | 2-arm RCT; **Tx:** Counseling (55)  **Ct:** Routine care (55) | Miscarriage  Mean age: 32.0 (6.3) | - **Recipient:** Mothers - **Content:**   - **Tx:** STress-And-coping suppoRT (START) intervention: 1) Consultation (after women made their abortion appointment, 30 mins); 2) Information booklet (during the consultation, one copy per participant); 3) The START platform (unlimited access till the closure of the project); 4) Hotline (available during work hours)   - **Ct:** Routine care - **Modality:** Blended (Face-to-face+ Online Wechat public profile page) - **Format**: Individual-based - **Intensity**: 1-session, 30-minnute - **Time point of data collection**: pre-test, posttest, and 6-week follow-up - **Retention rate**: post-intervention: 77.27%; follow-up: 74.55%. - **Setting:** Hospital - **Provider(s)**: Nurse - **Theoretical framework:** Transactional Model of Stress and Coping | - Depression: Patient Health Questionnaire-9 (PHQ-9) - Perceived social support: Medical Outcomes Study Social Support Scale (MOSSS-5) |

# Supplementary Table 4. Measurement instruments used in the non-pharmacological interventions

| Outcomes | Measurements | References |
| --- | --- | --- |
| Grief (n_number of RCTs_=12) | Perinatal Grief Scale (PGS; Toedter et al. [1988]) (n=7) | (Barat et al., 2020; Huberty et al., 2020; Johnson, 2009; Johnson & Langford, 2015; Mecdi Kaydirak & Aslan, 2021; Navidian & Saravani, 2018; Navidian et al., 2017; Rocha et al., 2018; Rosenbaum et al., 2015) |
|  | Perinatal Bereavement Grief Scale (PBGS; Ritsher & Neugebauer [2002] ) (n=1) | (Johnson et al., 2016) |
|  | Self-developed Perinatal Grief Scale (Simpson et al., 2015) (n=1) | (Simpson et al., 2015) |
|  | Inventory of Complicated Grief (ICG; Prigerson et al. [1995] ) (n=2) | (Kersting et al., 2013; Kersting et al., 2011) |
|  | Miscarriage Grief Inventory (MGI; Nikcevic et al. [1999]) (n=1) | (Swanson et al., 2009) |
| Stress (n=5) | Perceived Stress Scale (PSS; Sheldon Cohen et al. [1983]) (n=3) | (Chang et al., 2021; Hung et al., 2023; Jensen et al., 2021) |
|  | Depression Anxiety Stress Scale - 21-Stress Subscale (DASS-21; Lovibond & Lovibond [1995]) (n=1) | (Nasrollahi et al., 2022) |
|  | Depression Anxiety Stress Scale - 42 -Stress Subscale (DASS-42; Lovibond & Lovibond [1995]) (n=1) | (Haghighi et al., 2022) |
| Post-traumatic stress disorder (n=5) | The Impact of Event Scale (IES-R; Creamer et al. [2003]) (n=5) | (Huberty et al., 2020; Kersting et al., 2013; Kersting et al., 2011; Séjourné et al., 2010; Sun et al., 2018) |
| Depression (n=17) | Beck Depression Inventory (BDI; Beck et al. [1961]) (n=1) | (Kong et al., 2014) |
|  | Beck Depression Inventory-II (BDI-II; (Beck et al. [1996] ) (n=2) | (Johnson et al., 2016; Rocha et al., 2018) |
|  | Brief Symptom Inventory (BSI) – Depression subscale (Derogatis, 1975) (n=2) | (Kersting et al., 2013; Kersting et al., 2011) |
|  | Center for Epidemiologic Studies-Depression (CES-D; Radloff [1977]) (n=2) | (Rosenbaum et al., 2015; Swanson et al., 2009) |
|  | Depression Anxiety Stress Scale - 21-Depression Subscale (Lovibond & Lovibond, 1995) (n=1) | (Nasrollahi et al., 2022) |
|  | Depression Anxiety Stress Scale - 42 - Anxiety Subscale (DASS-42; Lovibond & Lovibond [1995]) (n=1) | (Haghighi et al., 2022) |
|  | Edinburgh Prenatal Depression Scale (EPDS; Cox et al. [1987]) (n=3) | (Chang et al., 2021; Hung et al., 2023; Sun et al., 2018) |
|  | Hamilton Depression Scale (HAM-D; Hamilton [1960]) (n=1) | (Simpson et al., 2015) |
|  | Hospital Anxiety and Depression Scale (HADS; Zigmond & Snaith [1983]) (n=2) | (Barat et al., 2020; Séjourné et al., 2010) |
|  | Patient Health Questionnaire-9 (PHQ-9; Kroenke et al. [2001]) (n=2) | (Huberty et al., 2020; Wang et al., 2023) |
| Anxiety (n=10) | Brief Symptom Inventory (BSI) - Anxiety Subscale (Derogatis, 1975) (n=2) | (Kersting et al., 2013; Kersting et al., 2011) |
|  | Depression Anxiety Stress Scale - 21-Anxiety Subscale (DASS-21; Lovibond & Lovibond [1995]) (n=1) | (Nasrollahi et al., 2022) |
|  | Depression Anxiety Stress Scale - 42 - Anxiety Subscale (DASS-42; Lovibond & Lovibond [1995]) (n=1) | (Haghighi et al., 2022) |
|  | Hamilton Anxiety Scale (HAM-A; Hamilton [1960]) (n=1) | (Simpson et al., 2015) |
|  | Hospital Anxiety and Depression Scale - Anxiety Subscale (HADS; Zigmond & Snaith [1983]) (n=2) | (Barat et al., 2020; Séjourné et al., 2010) |
|  | State-Trait Anxiety Inventory (STAI; Newham et al. [2012]) (n=1) | (Huberty et al., 2020) |
|  | Zung Anxiety Scale (Zung, 1971) (n=1) | (Rocha et al., 2018) |
|  | State Anxiety Inventory-2 (STAI-2; Spielberger et al. [1971]) (n=1) | (Mecdi Kaydirak & Aslan, 2021) |
| Distress (n=4) | Depression Anxiety Stress Scale -21 (DASS-21; Lovibond & Lovibond [1995]) (n=1) | (Nasrollahi et al., 2022) |
|  | Depression Anxiety Stress Scale - 42 (DASS-42; Lovibond & Lovibond [1995]) (n=1) | (Haghighi et al., 2022) |
|  | Dyadic Adjustment Scale (DAS; Antill & Cotton [1982]) (n=1) | (J. E. Johnson et al., 2016) |
|  | General Health Questionnaire (GHQ-12; Goldberg [1978]) (n=1) | (Kong et al., 2014) |
| Sleep quality (n=4) | Pittsburgh Sleep Quality Index (PSQI; Buysse et al. [1989]) (n=3) | (Chang et al., 2021; Huberty et al., 2020; Hung et al., 2023) |
|  | Visual Analog Scale (VAS; Wewers & Lowe [1990]) (n=1) | (Mecdi Kaydirak & Aslan, 2021) |
| Perceived social support (n=6) | Interpersonal Support Evaluation List- Short Form (ISEL-SF; Cohen & Hoberman [1983]) (n=2) | (Chang et al., 2021; Hung et al., 2023) |
|  | Medical Outcomes Study Social Support Scale (MOSSS-5; Sherbourne & Stewart [1991]) (n=1) | (Wang et al., 2023) |
|  | Multidimensional Scale for Perceived Social Support (MSPSS; Dahlem et al. [1991] ) (n=1) | (Johnson et al., 2016) |
|  | Scale of Ways of Coping with Stress (SWCS; Sahın & Durak [1995]) (n=1) | (Mecdi Kaydirak & Aslan, 2021) |
|  | Social Provisions Scale (SPS; Cutrona [1986] ) (n=1) | (Rosenbaum et al., 2015) |

**
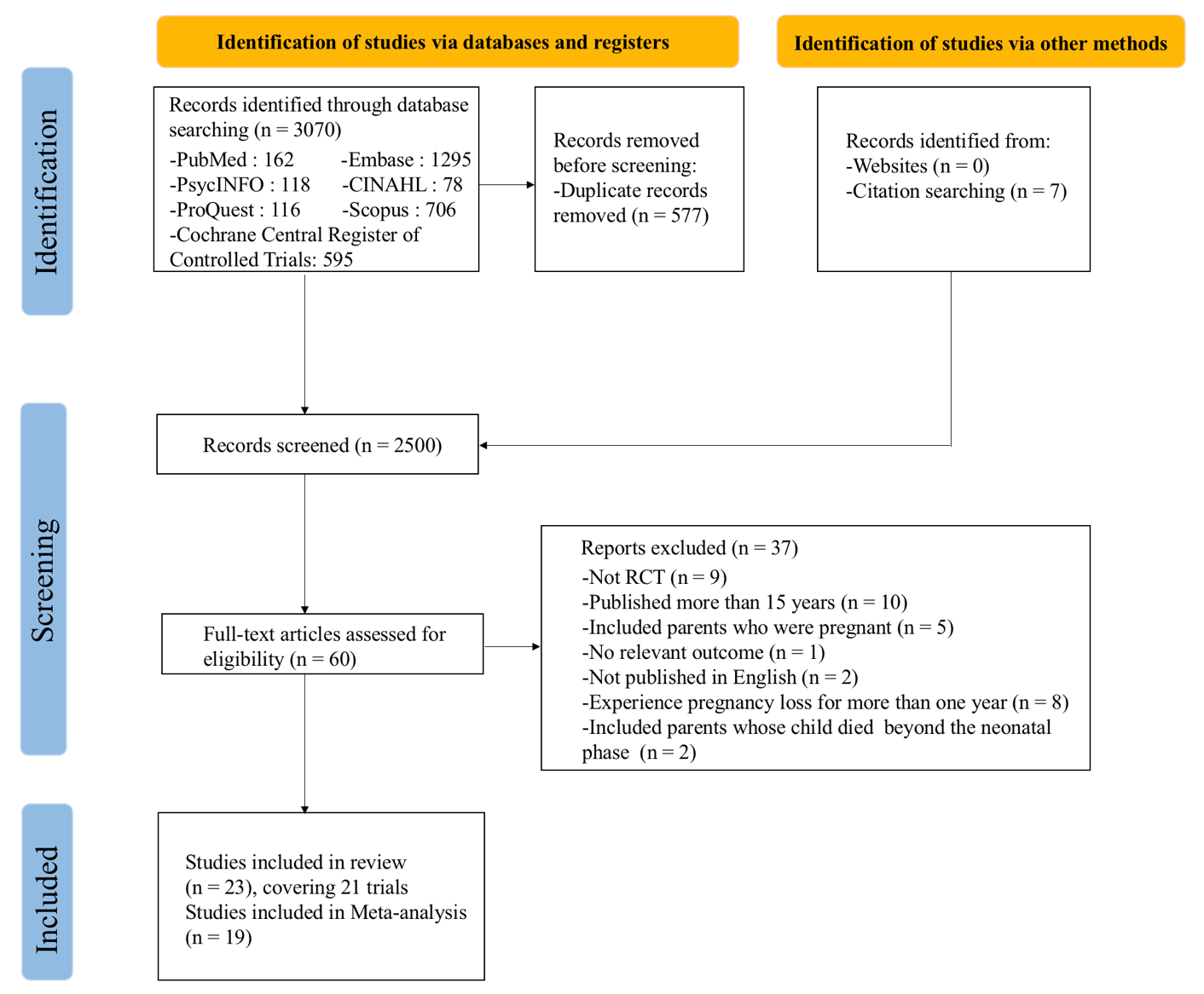
**

# Supplementary Figure 1. Flow Diagram for Searching and Selection of Articles

**
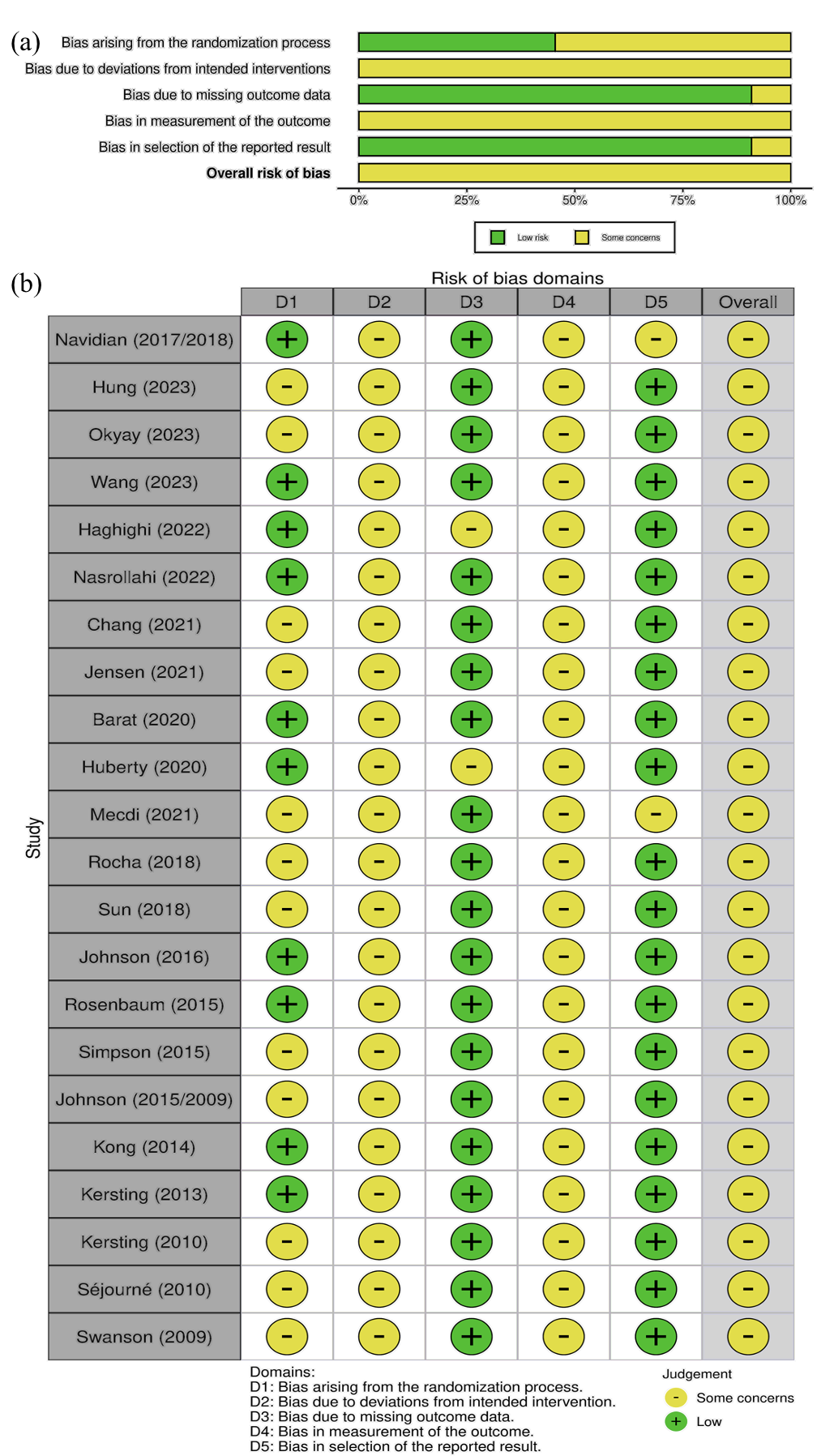
**

# Supplementary Figure 2. (a) Risk of bias graph about each risk of bias item presented as percentages across all included studies; (b) Summary of risk of bias for each study assessed by Cochrane Collaboration's tool.

**
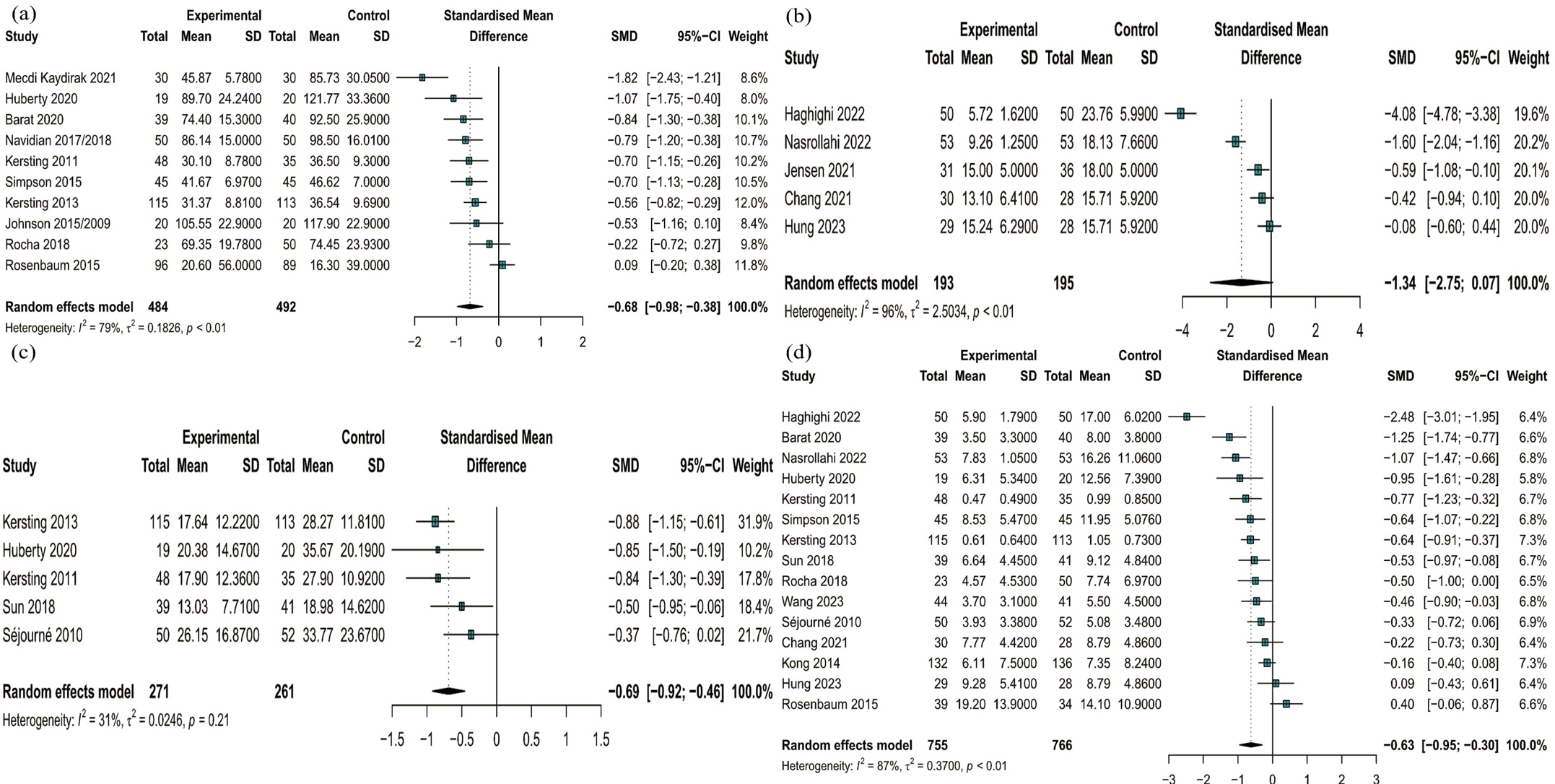
**

# Supplementary Figure 3. Forest plots: effect of non-pharmacological interventions on (a) parental grief; (b) stress; (c) post-traumatic stress disorder; and (d) depression for parents with pregnancy loss.

**
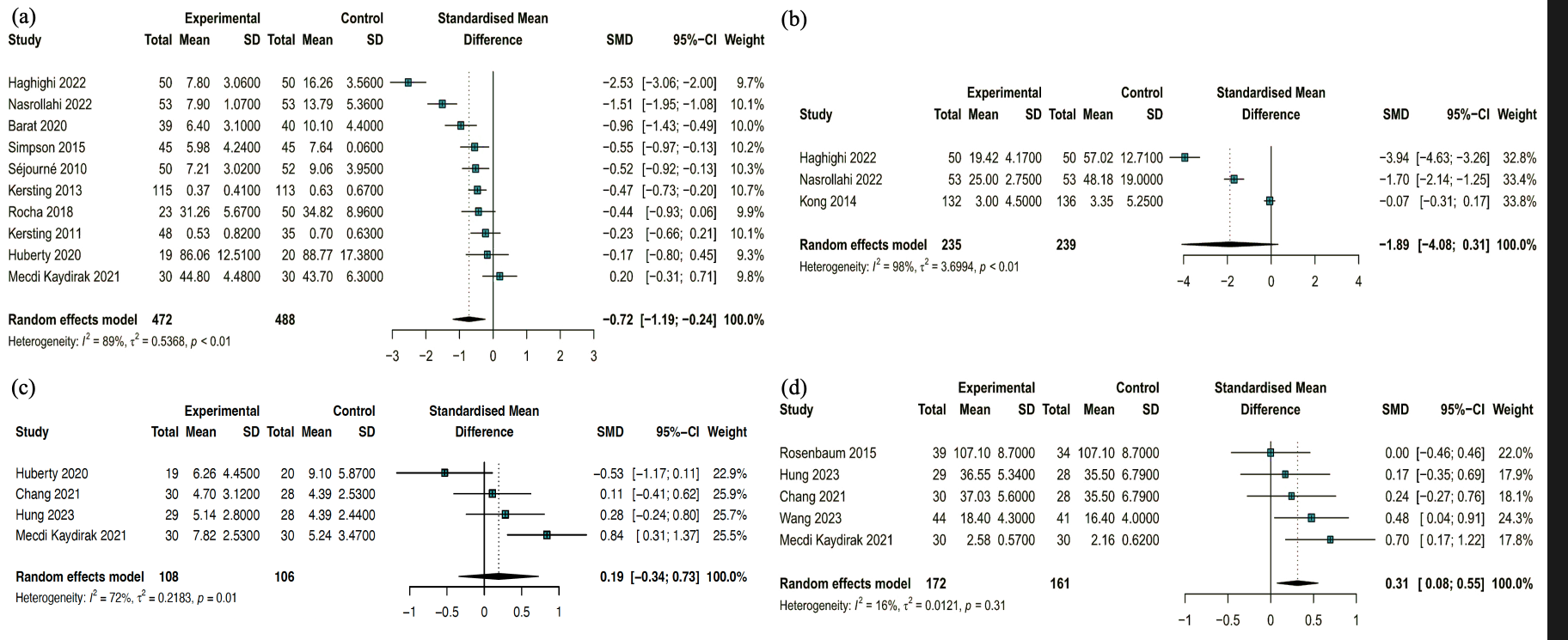
**

# Supplementary Figure 4. Forest plots: effect of non-pharmacological interventions on (a) parental anxiety; (b) distress; (c) sleep quality; and (d) perceived social support for parents with pregnancy loss.


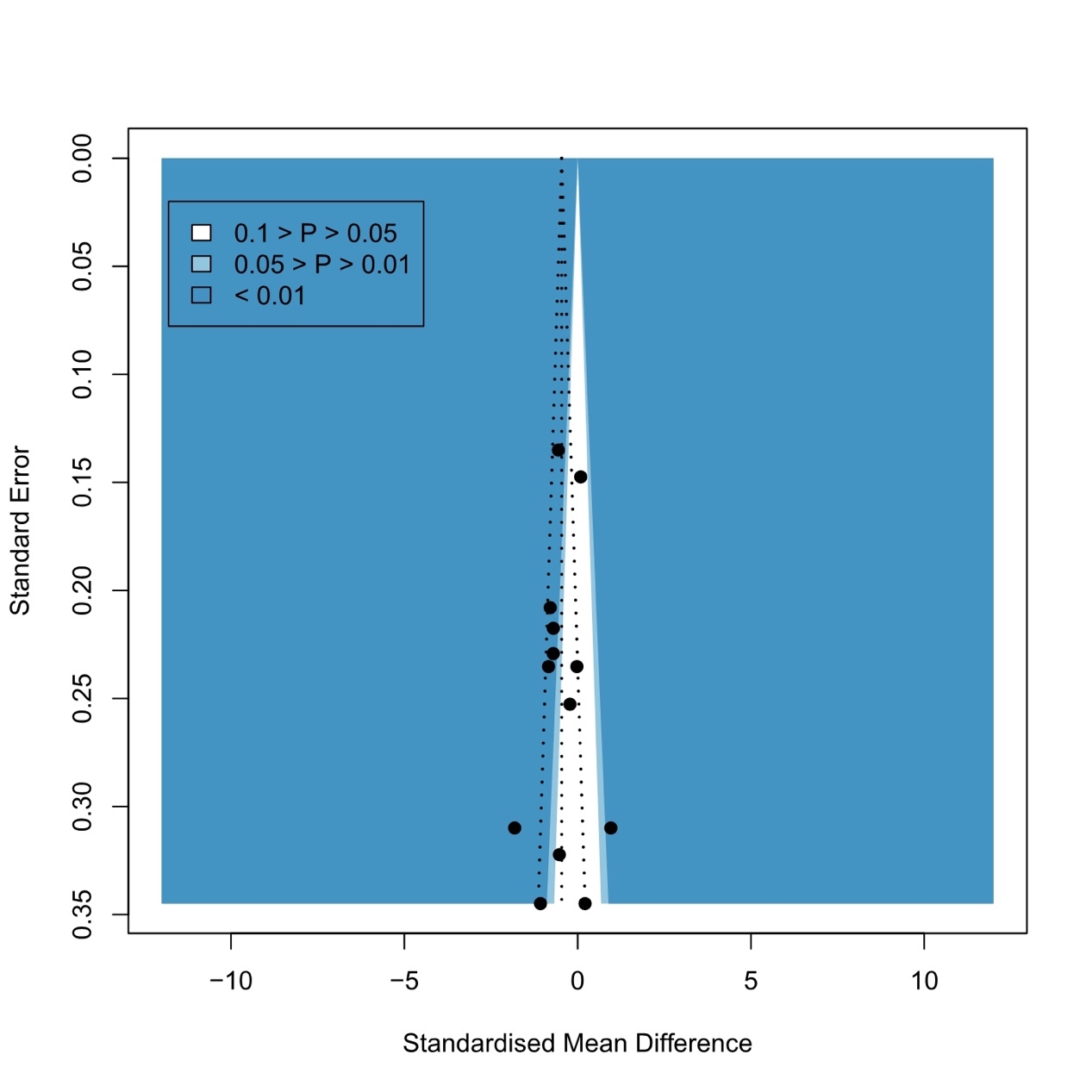


*Egger test -Test for Funnel Plot Asymmetry: t=-2.08, df=8, p=0.0715

# Supplementary Figure 5. The funnel plot and Egger's test for the effects of the non-pharmacological interventions on grief for parents with perinatal loss.


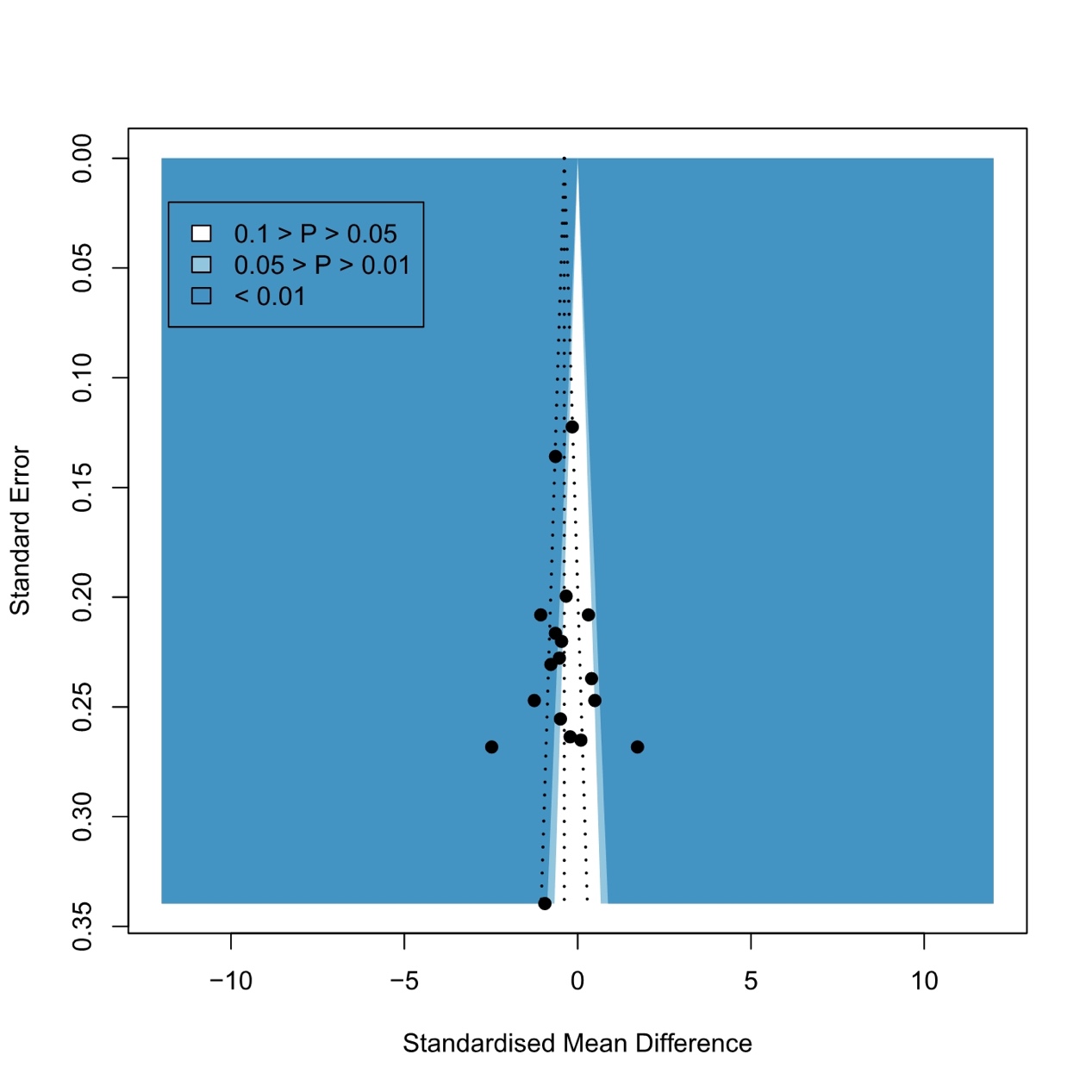


*Egger test -Test for Funnel Plot Asymmetry: t=-1.12, df=13, p=0.2840

# Supplementary Figure 6. The funnel plot and Egger's test for the effects of the non-pharmacological interventions on depression for parents with perinatal loss.


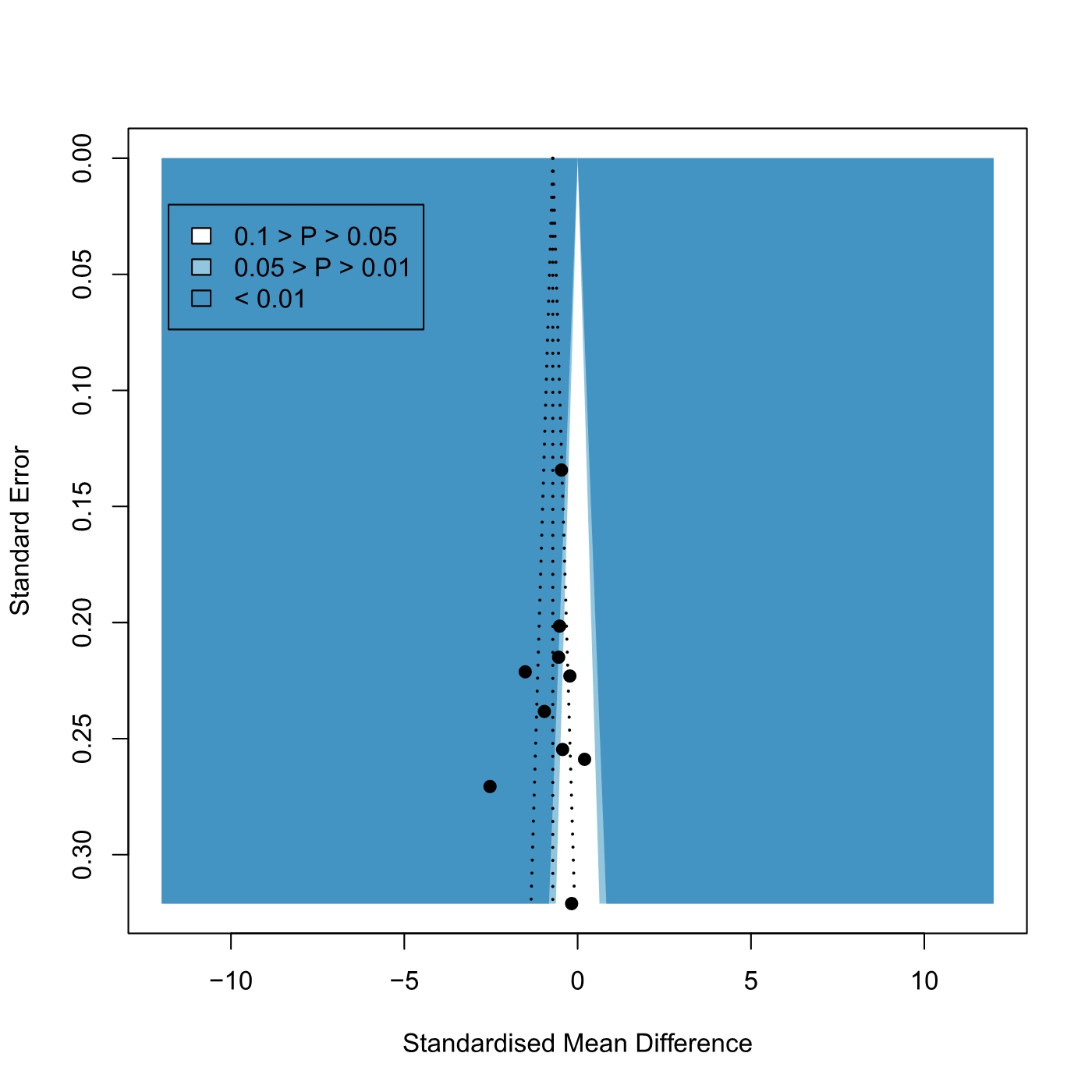


*Egger test -Test for Funnel Plot Asymmetry: t=-0.54, df=8, p=0.6070

# Supplementary Figure 7. The funnel plot and Egger's test for the effects of the non-pharmacological interventions on anxiety for parents with perinatal loss.


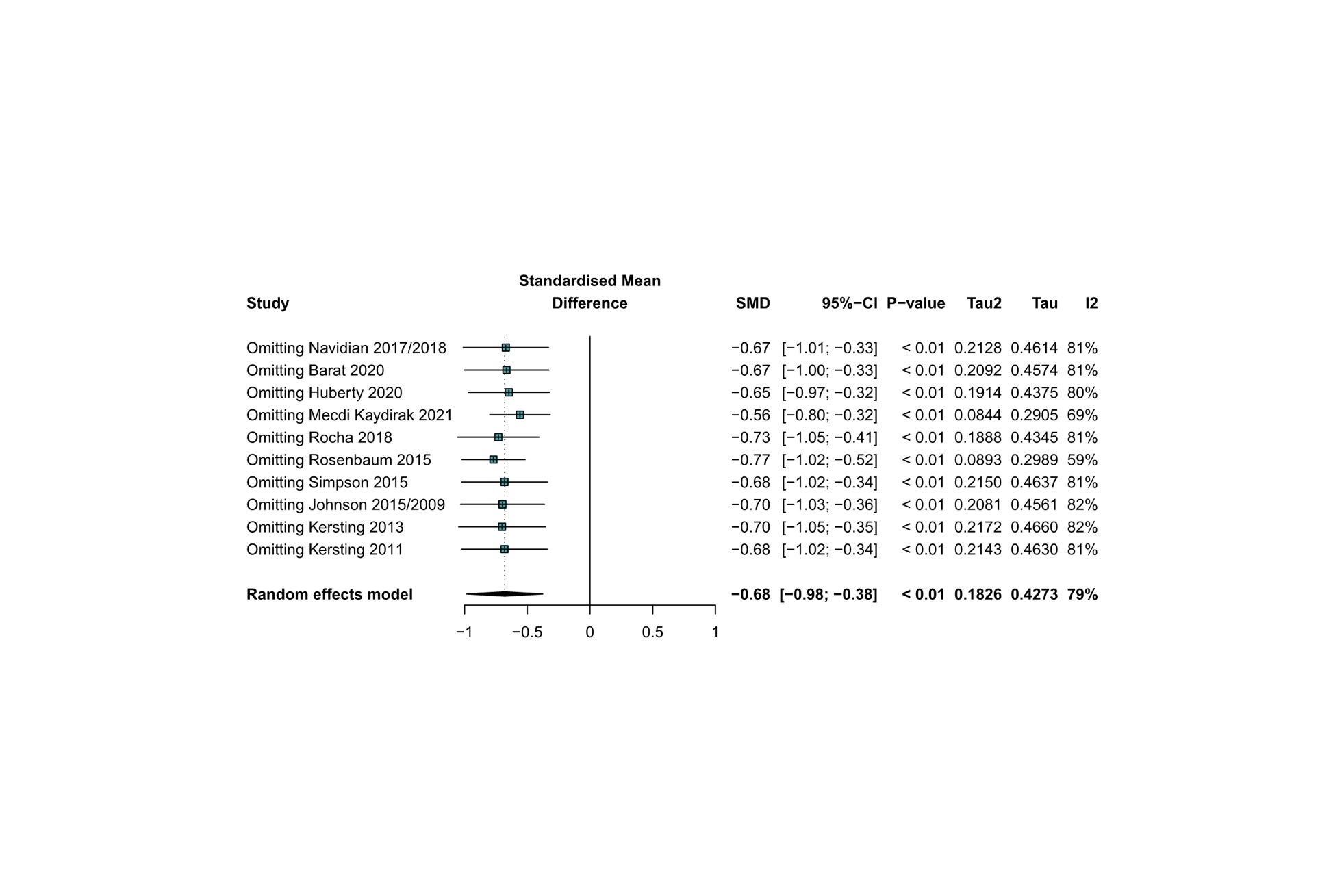


# Supplementary Figure 8. Sensitivity analysis for the effects of the non-pharmacological interventions on parental grief for parents with perinatal loss.


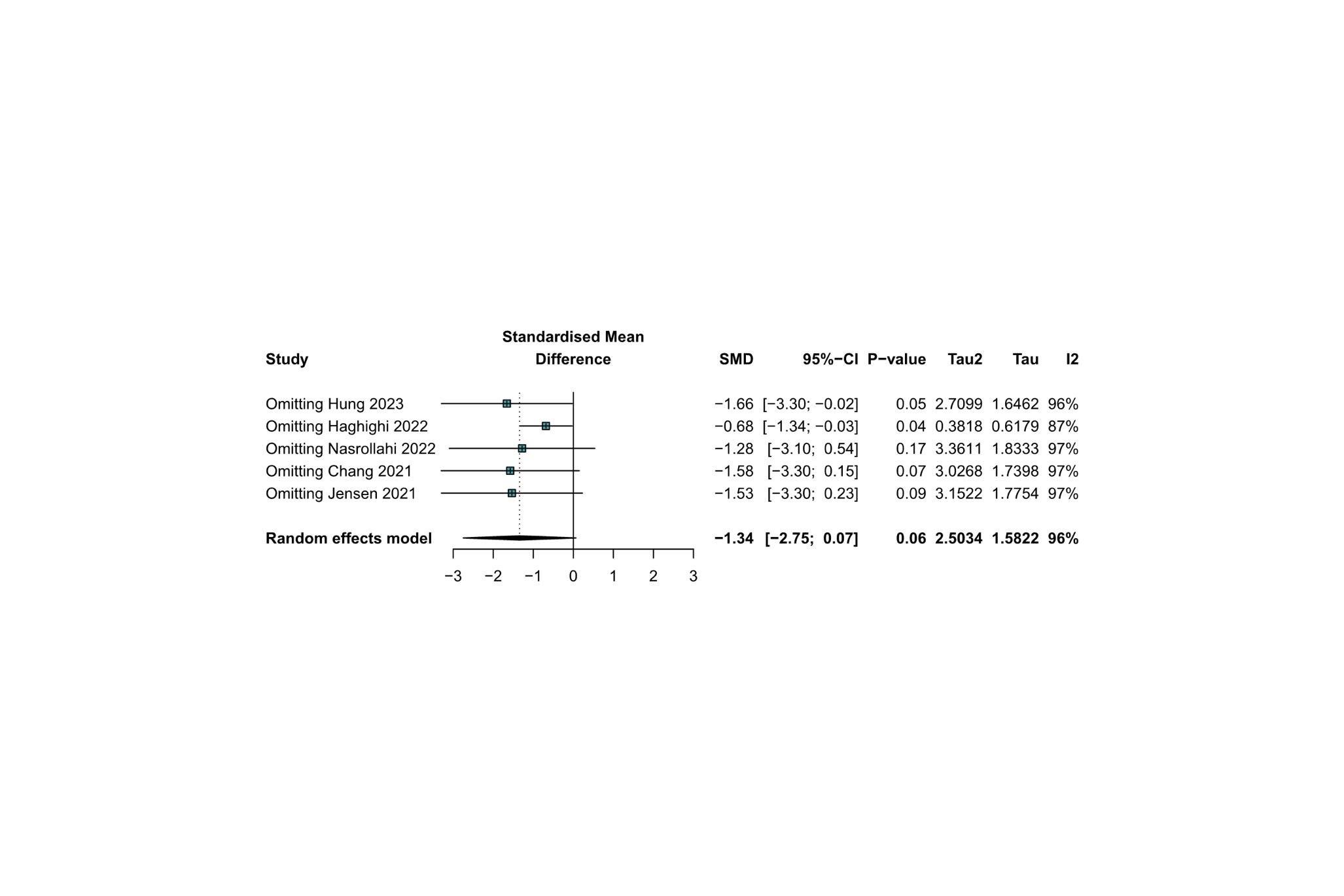


# Supplementary Figure 9. Sensitivity analysis for the effects of the non-pharmacological interventions on parental stress for parents with perinatal loss.


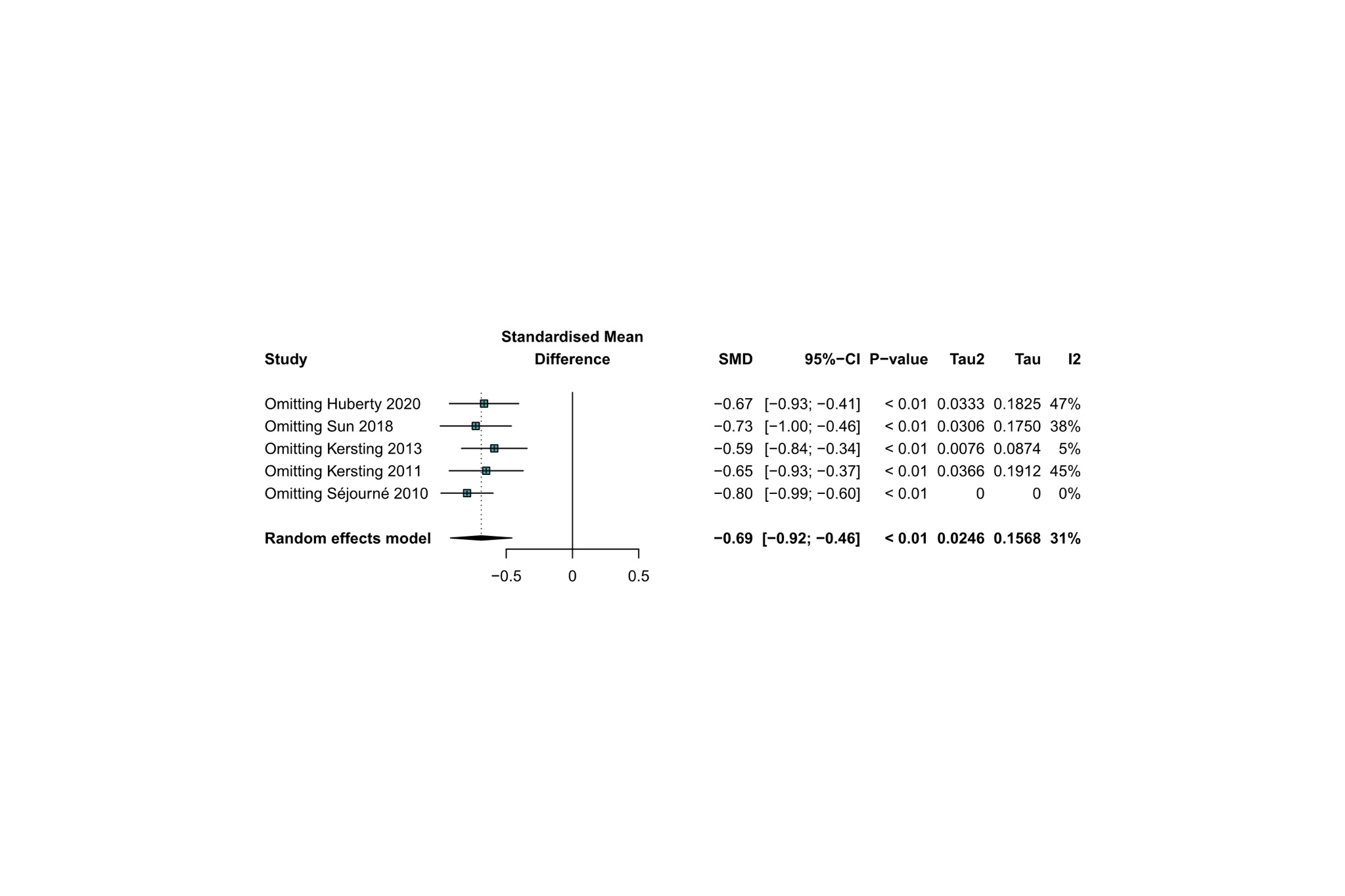


# Supplementary Figure 10. Sensitivity analysis for the effects of the non-pharmacological interventions on parental post-traumatic stress disorder for parents with perinatal loss.


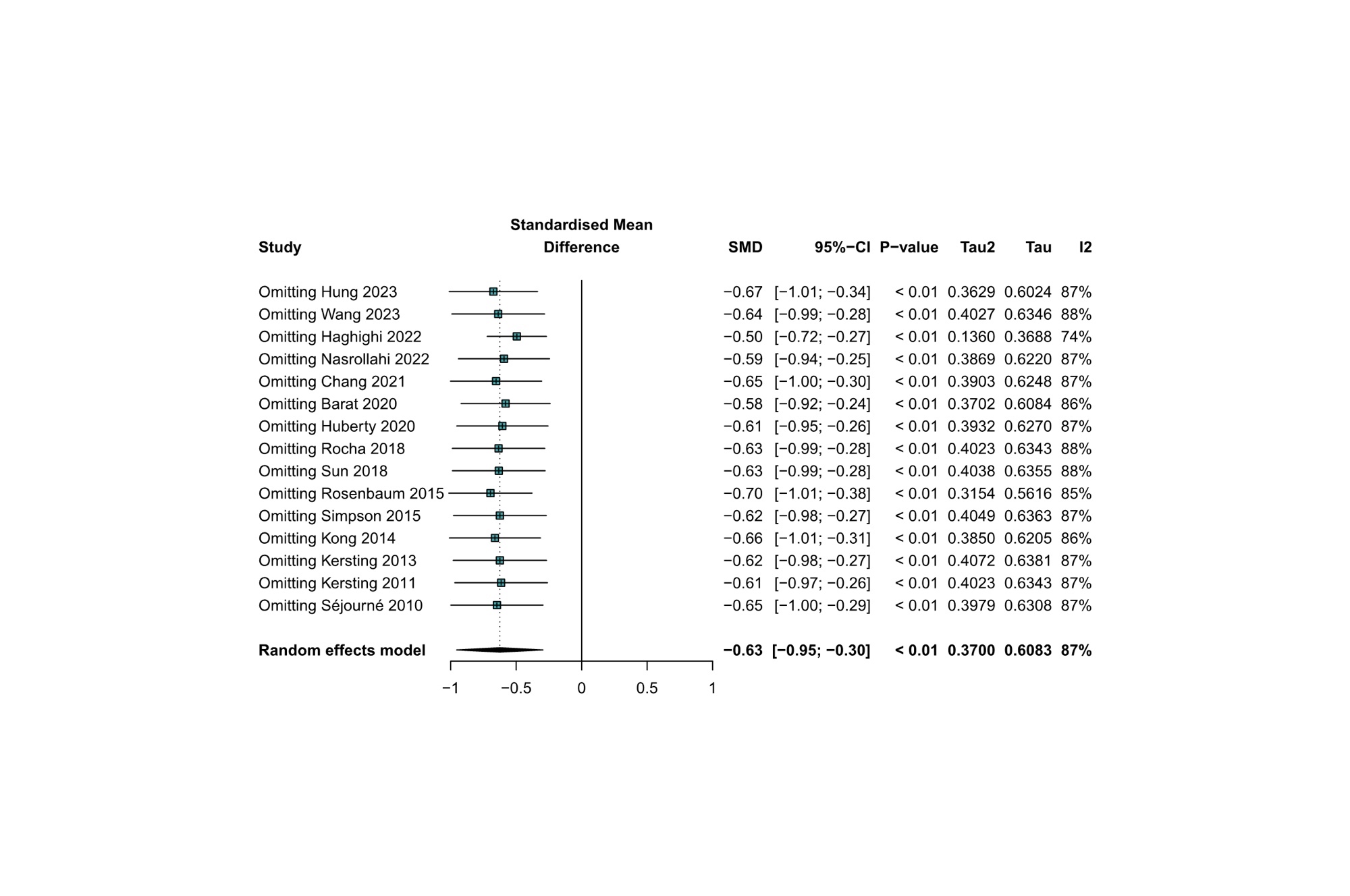


# Supplementary Figure 11. Sensitivity analysis for the effects of the non-pharmacological interventions on parental depression for parents with perinatal loss.


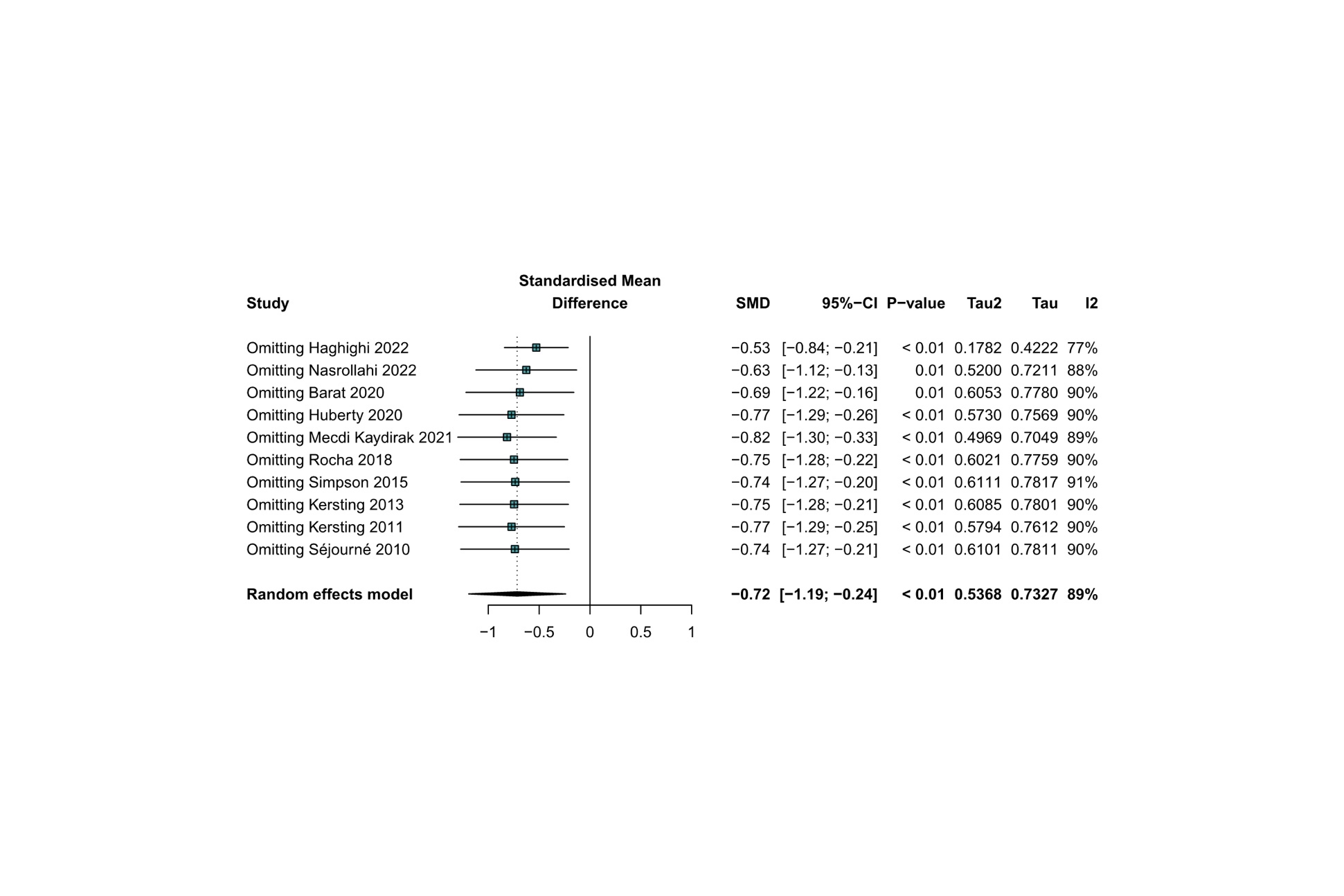


# Supplementary Figure 12. Sensitivity analysis for the effects of the non-pharmacological interventions on parental anxiety for parents with perinatal loss.


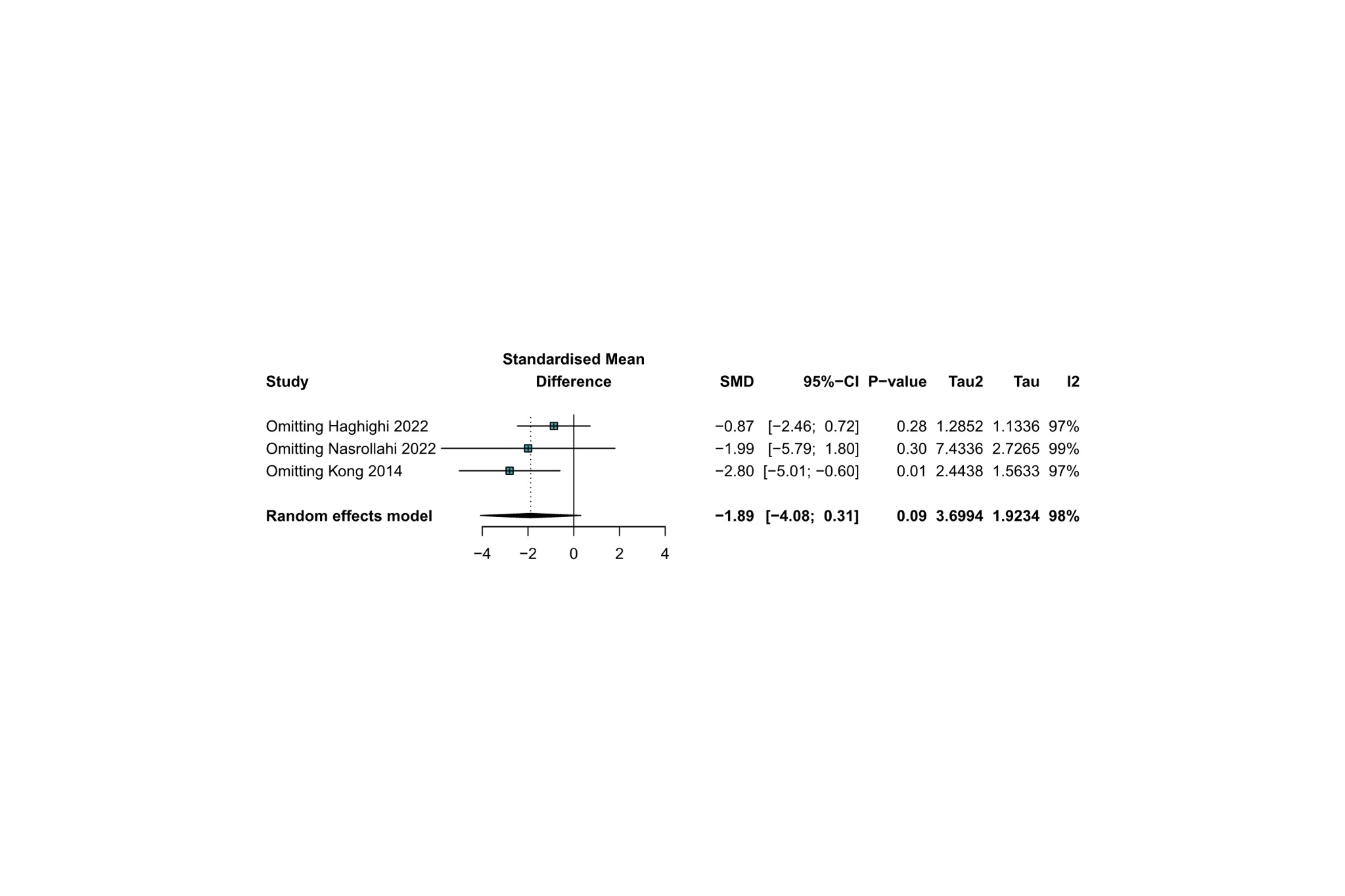


# Supplementary Figure 13. Sensitivity analysis for the effects of the non-pharmacological interventions on parental distress for parents with perinatal loss.


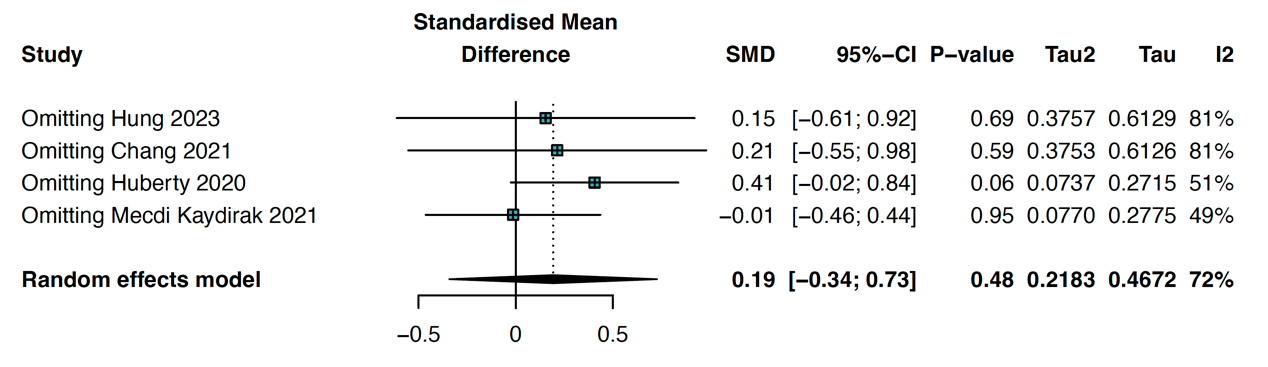


# Supplementary Figure 14. Sensitivity analysis for the effects of the non-pharmacological interventions on parental sleep quality for parents with perinatal loss.


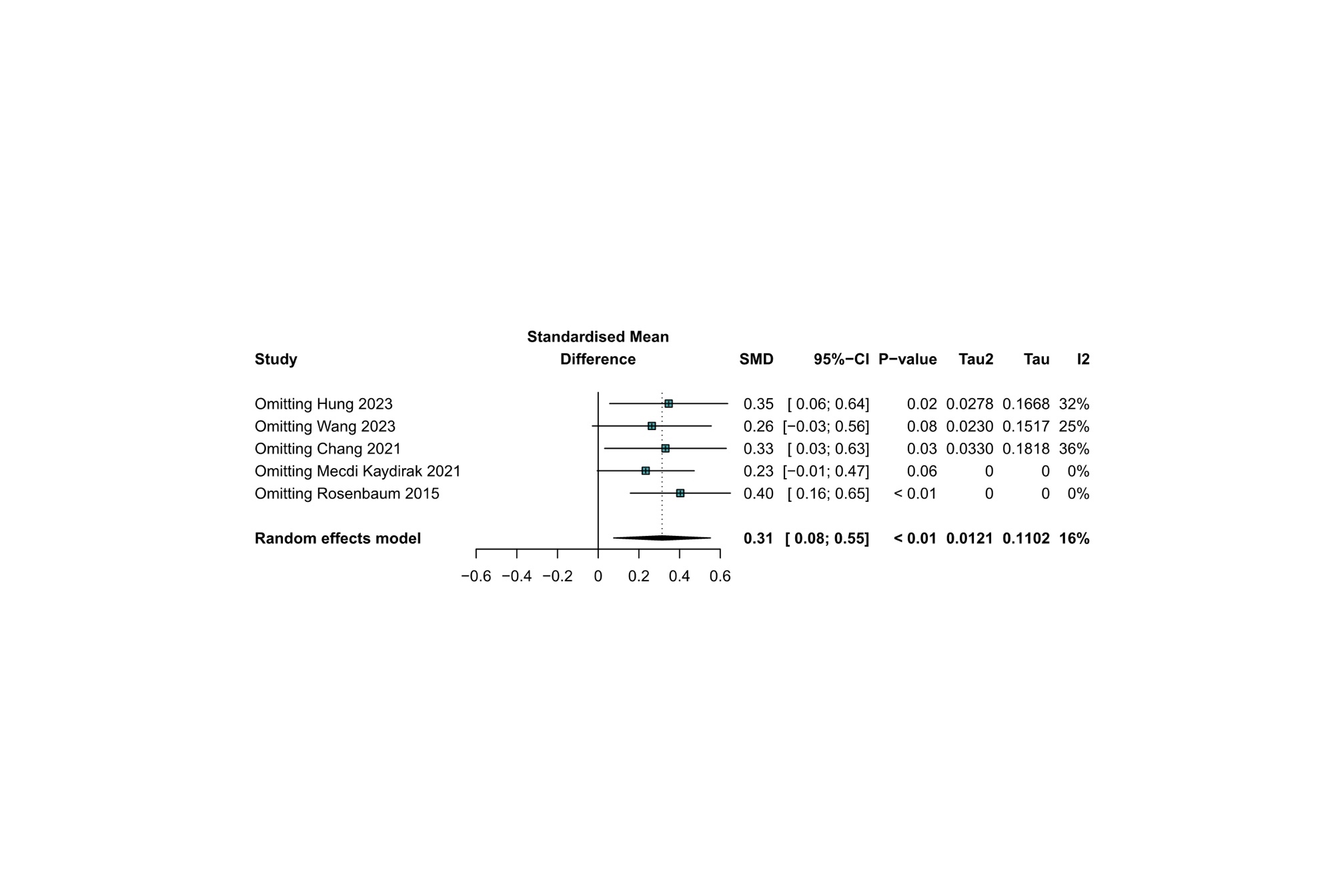


# Supplementary Figure 15. Sensitivity analysis for the effects of the non-pharmacological interventions on parental perceived social support for parents with perinatal loss.

# Explanation for the Results of Sensitivity Analysis

The sensitivity analyses conducted in this meta-analysis revealed that the results pertaining to parental grief, PTSD, depression, anxiety, and sleep quality were robust and consistent. However, a degree of variability was observed in the evaluations of parental stress, distress, and perceived social support, where positive effects were either observed or reversed with the exclusion of certain studies.

Specifically, for parental stress, the positive effects for non-pharmacological intervention were observed if removing the study conducted by Hung et al. (2023) (g=-1.66, 95% CI: [-3.30, -0.02], P-value for 95% CI = 0.05, I^2^=96%; Supplementary Figure 9) or Haghighi et al. (2022) (g=-0.68, 95% CI: [-1.33, -0.03], P = 0.04, I^2^=87%; Supplementary Figure 9).

Similarly, for parental distress, the positive effects for non-pharmacological intervention were observed if removing the study conducted by Kong et al. (2014) (g=-2.80, 95% CI: [-4.06, -0.60], P = 0.01, I^2^=97%; Supplementary Figure 13).

As for parental perceived social support, the positive effects for non-pharmacological intervention disappeared if removing the study conducted by Wang et al. (2023) (g=0.26, 95% CI: [-0.03, 0.56], P= 0.08, I^2^=25%; Supplementary Figure 15) or Mecdi et al. (2023) (g=0.23, 95% CI: [-0.01, 0.47], P = 0.06, I^2^=0%; Supplementary Figure 15).


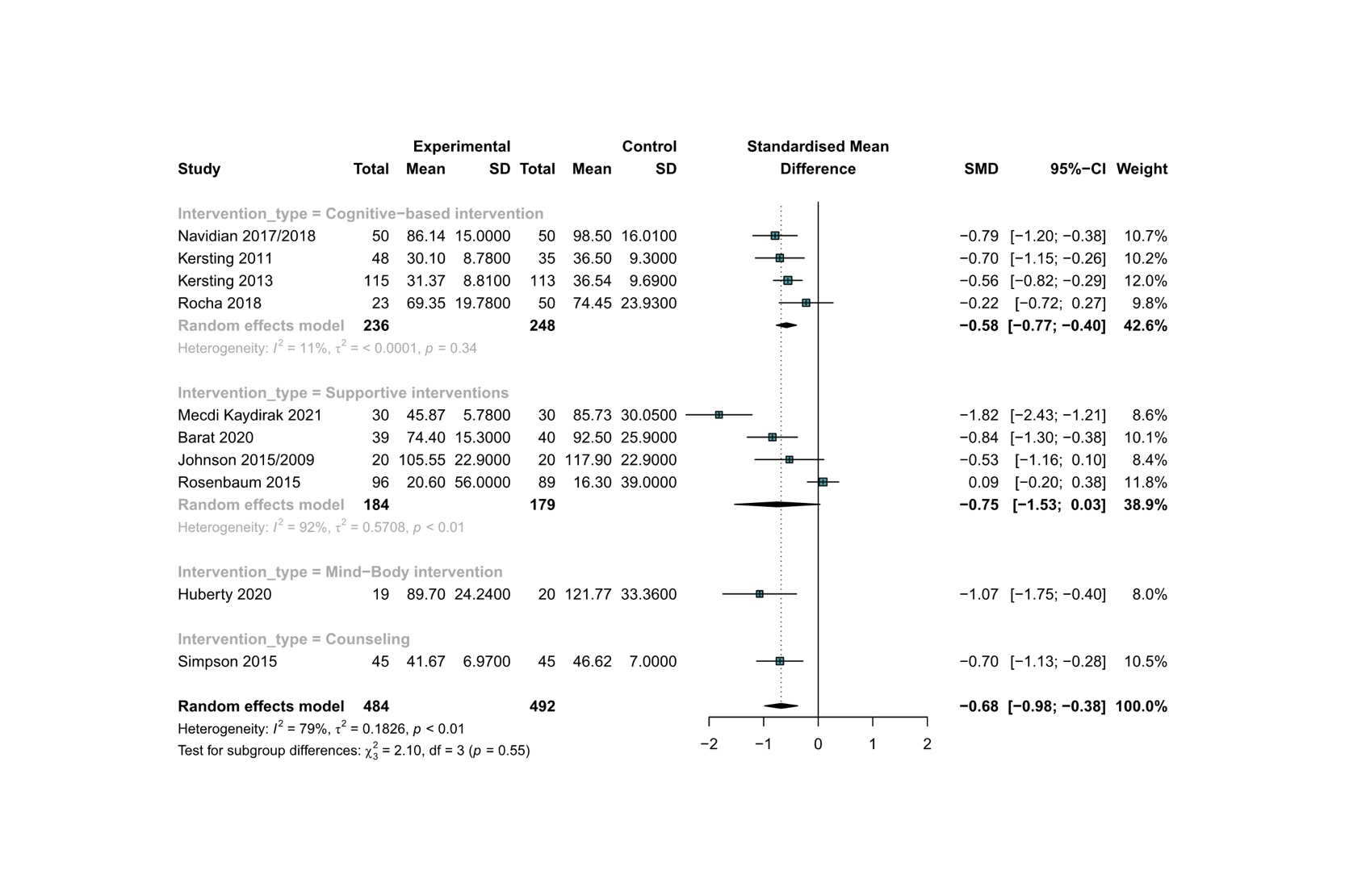


# Supplementary Figure 16. Subgroup analysis to compare the effects of different intervention types of the non-pharmacological interventions on parental grief for parents with perinatal loss.


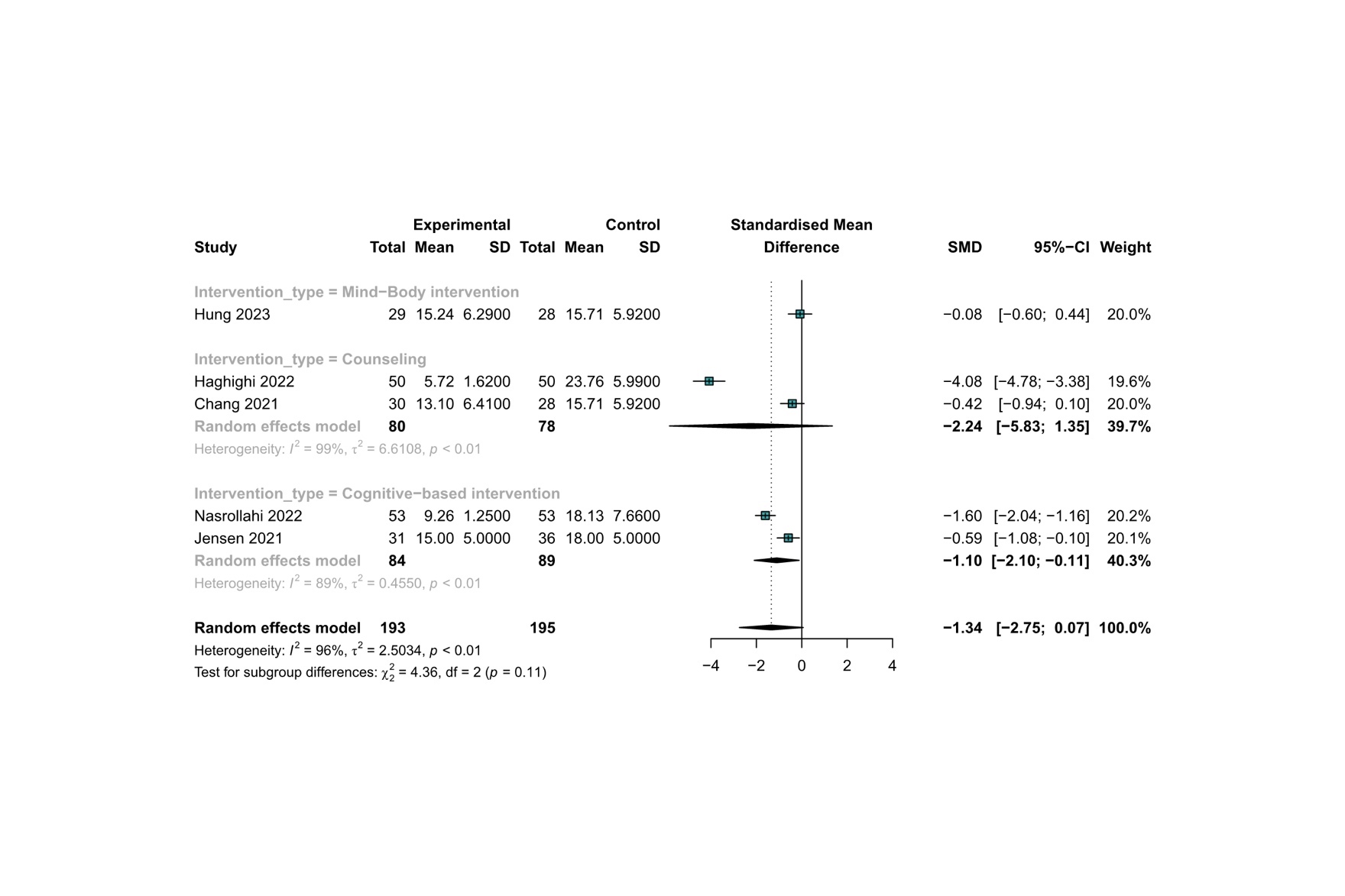


# Supplementary Figure 17. Subgroup analysis to compare the effects of different intervention types of the non-pharmacological interventions on parental stress for parents with perinatal loss.


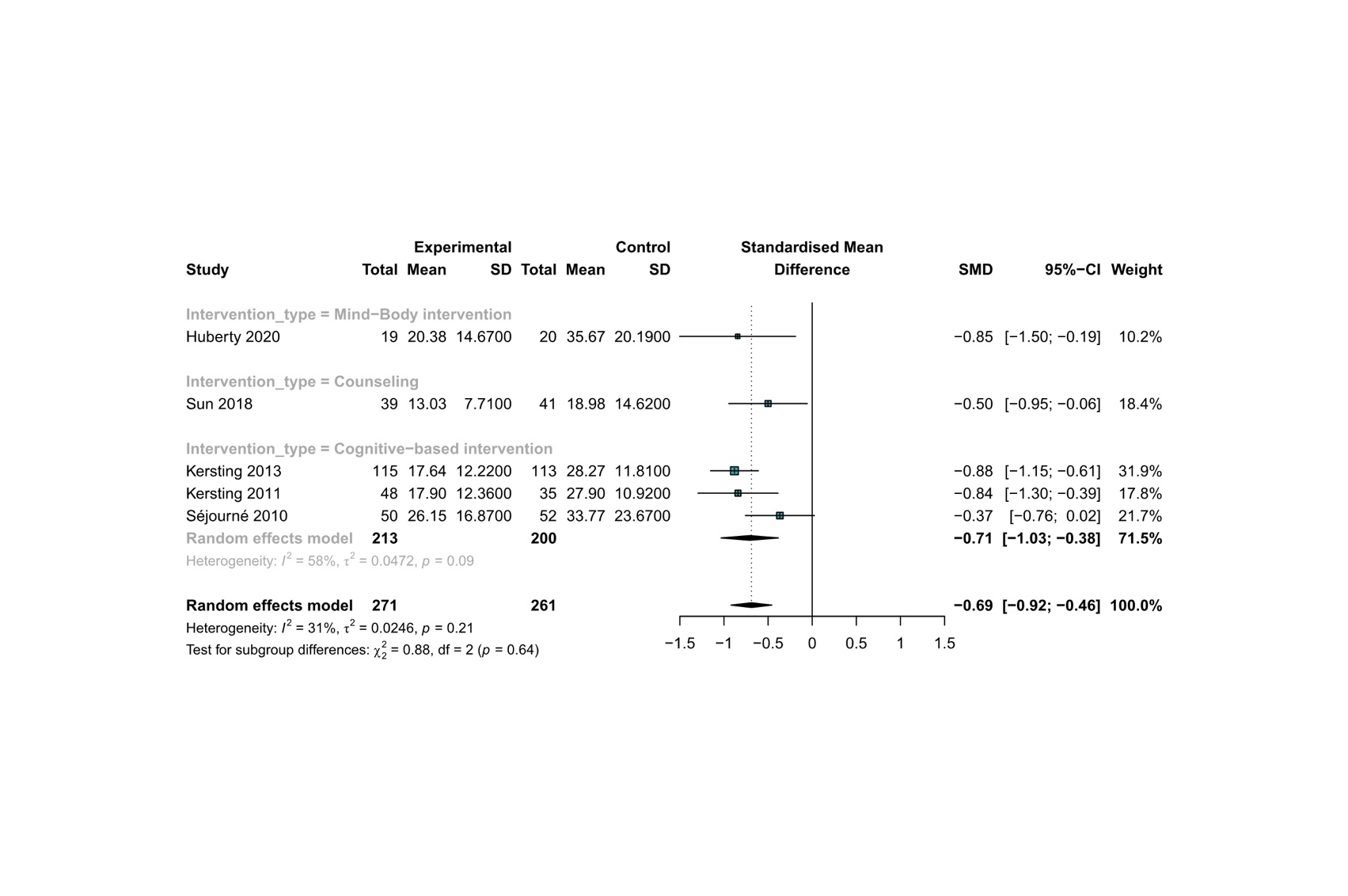


# Supplementary Figure 18. Subgroup analysis to compare the effects of different intervention types of the non-pharmacological interventions on parental post-traumatic stress disorder for parents with perinatal loss.


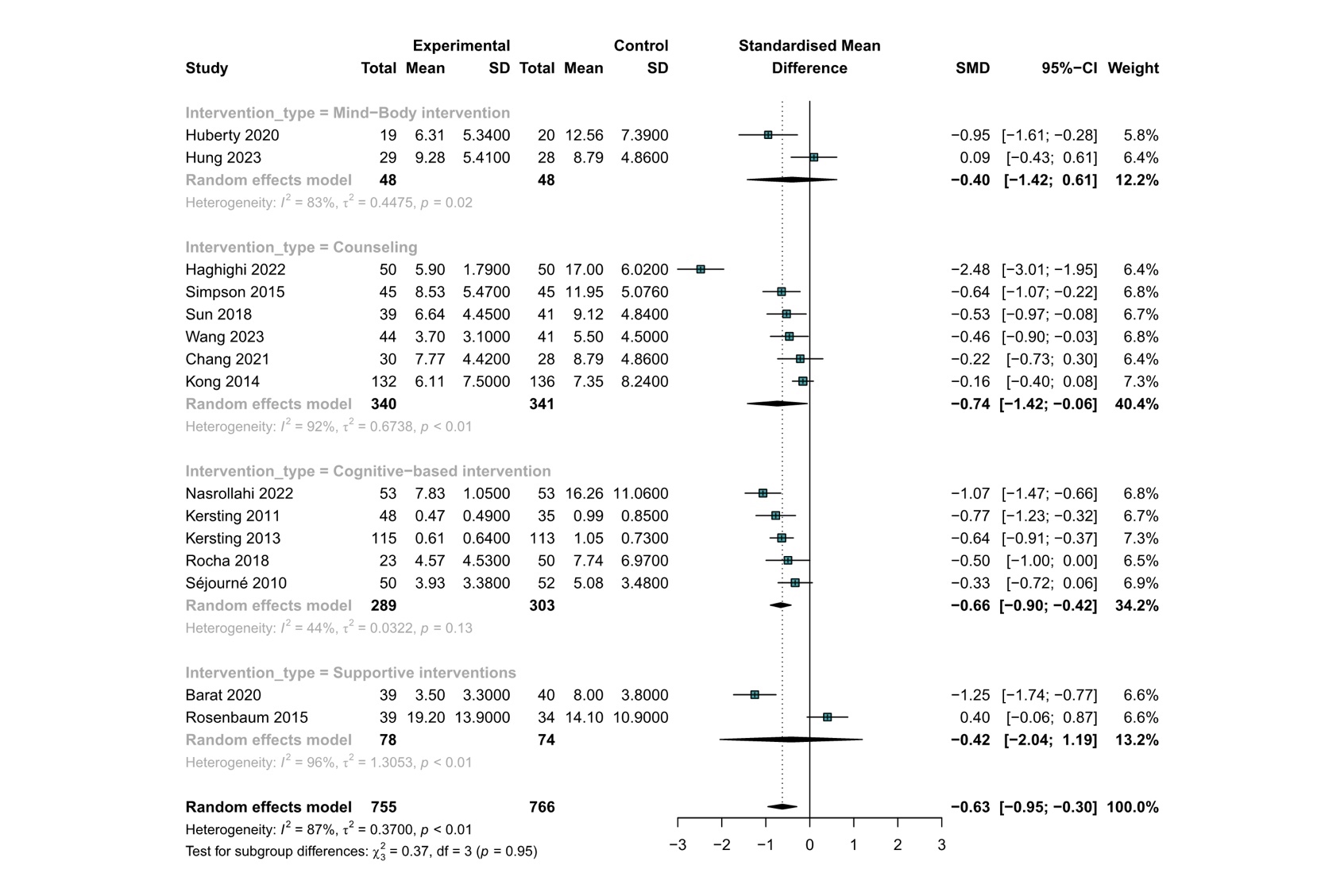


# Supplementary Figure 19. Subgroup analysis to compare the effects of different intervention types of the non-pharmacological interventions on parental depression for parents with perinatal loss.


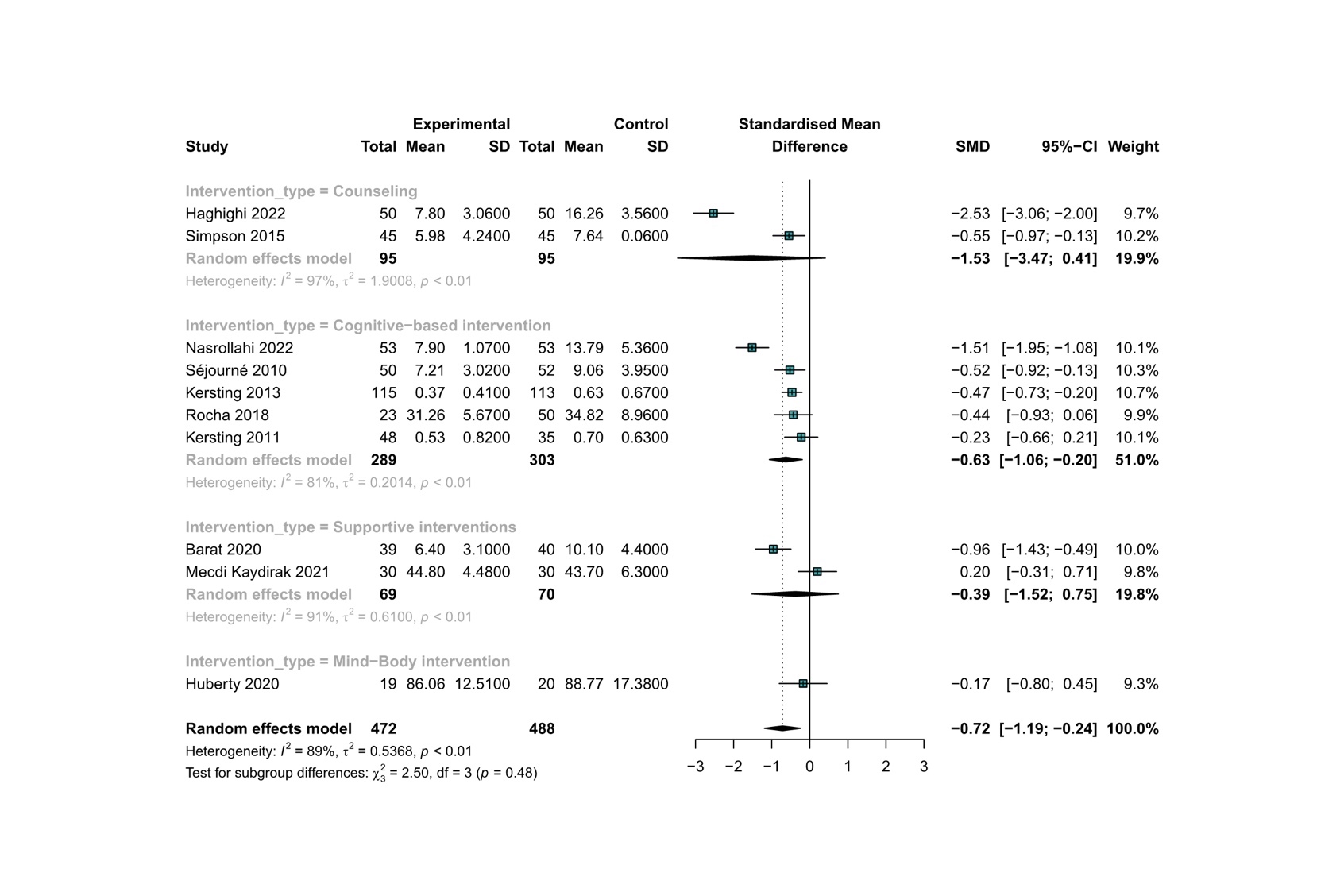


# Supplementary Figure 20. Subgroup analysis to compare the effects of different intervention types of the non-pharmacological interventions on parental anxiety for parents with perinatal loss.


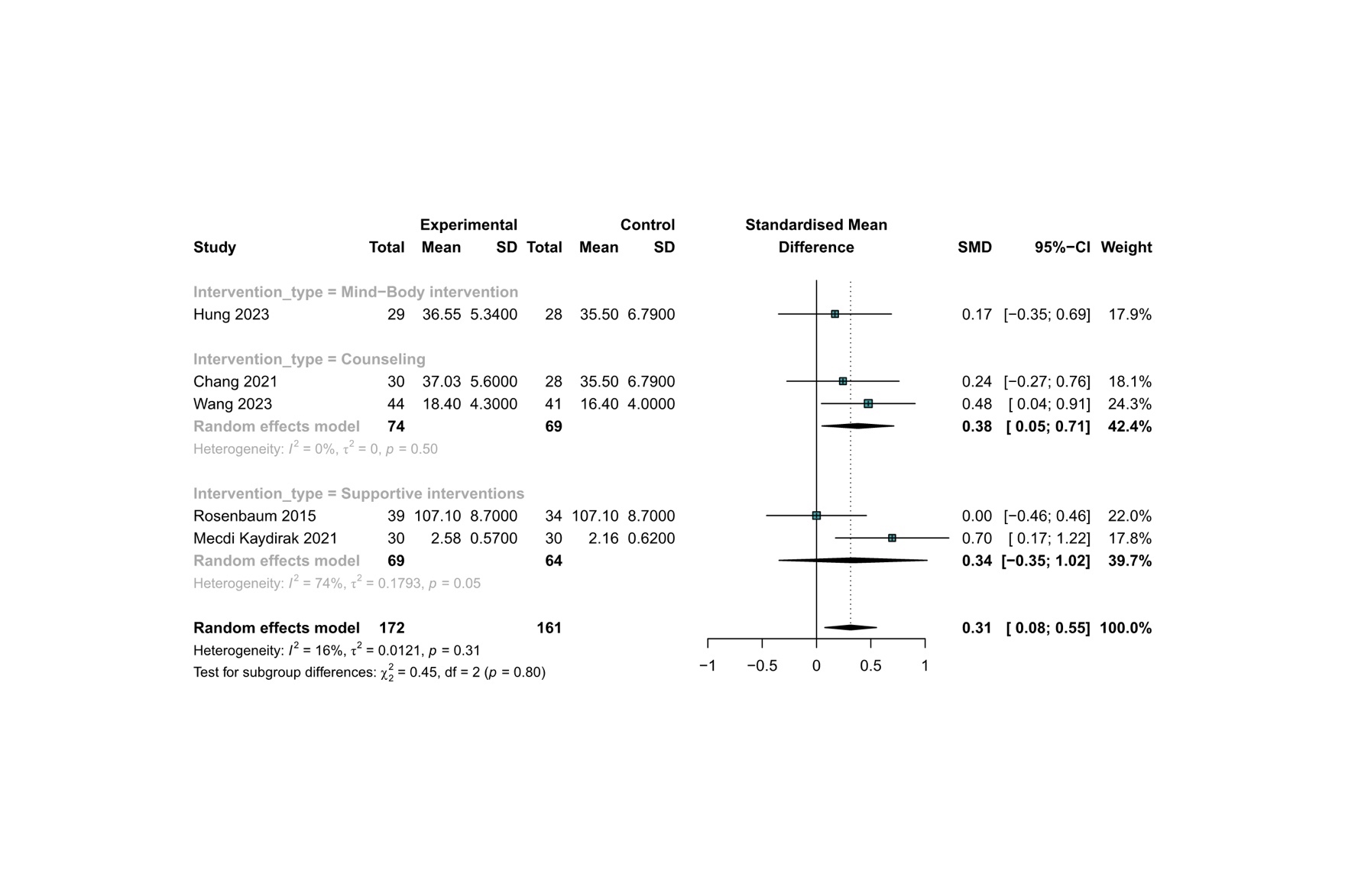


# Supplementary Figure 21. Subgroup analysis to compare the effects of different intervention types of the non-pharmacological interventions on perceived social support for parents with perinatal loss.

# Supplementary Table 5. Comparison of treatment effects among different intervention types of the non-pharmacological interventions for parents with perinatal loss.

| **Outcomes** | **Cognitive-based interventions** | | **Counseling** | | **Supportive interventions** | | **Mind-body interventions** | |
| --- | --- | --- | --- | --- | --- | --- | --- | --- |
|  | **k** | **Hedges’g (95%CI)** | **k** | **Hedges’g (95%CI)** | **k** | **Hedges’g (95%CI)** | **k** | **Hedges’g (95%CI)** |
| **Parental grief** | 4 | **-0.58 (95% CI: [-0.77, -0.40], P-value for chi-squared test = 0.34 I^2^=11%)** | 4 | -0.75 (95% CI: [-1.53, 0.03], P< 0.01, I^2^=92%) | 1 | - | 1 | - |
| **Parental stress** | 2 | **-1.10 (95% CI: [-2.10, -0.11], P<0.01, I^2^=89%)** | 2 | -2.24 (95% CI: [-5.83, 1.35], P<0.01, I^2^=99%) | 0 | - | 1 | - |
| **Parental post-traumatic stress disorder** | 3 | **-0.71 (95% CI: [-1.03, -0.38], P = 0.09, I^2^=58%)** | 1 | - | 0 | - | 1 | - |
| **Parental depression** | 5 | **-0.66 (95% CI: [-0.90, -0.42], P = 0.13, I^2^=44%)** | **6** | **-0.74 (95% CI: [-1.42, -0.06], P <0.01, I^2^=92%)** | 2 | **-0.42 (95% CI: [-2.04, -1.19], P<0.01, I^2^=96%)** | 2 | -0.40 (95% CI: [-1.42, 0.61], P = 0.02, I^2^=83%) |
| **Parental anxiety** | 5 | **-0.63 (95% CI: [-1.06, -0.20], P<0.01, I^2^=81%)** | 2 | -1.53 (95% CI: [-3.47, 0.41], P<0.01, I^2^=97%) | 2 | -0.39 (95% CI: [-1.52, 0.75], P<0.01, I^2^=91%) | 1 | - |
| **Parental distress** | 1 | - | 2 | -1.99 (95% CI: [-5.79, 1.80], P<0.01, I^2^=99%) | 0 | - | 0 | - |
| **Parental sleep quality** | 0 | - | 1 | - | 1 | - | 2 | -0.10 (95% CI: [-0.90, 0.69], P = 0.05, I^2^=73%) |
| **Perceived social support** | 0 | - | 2 | **0.38 (95% CI: [0.05, 0.71], P = 0.50, I^2^=0%)** | 2 | 0.34 (95% CI: [-0.35, 1.02], P = 0.05, I^2^=74%) | 1 | - |

k=number of RCT


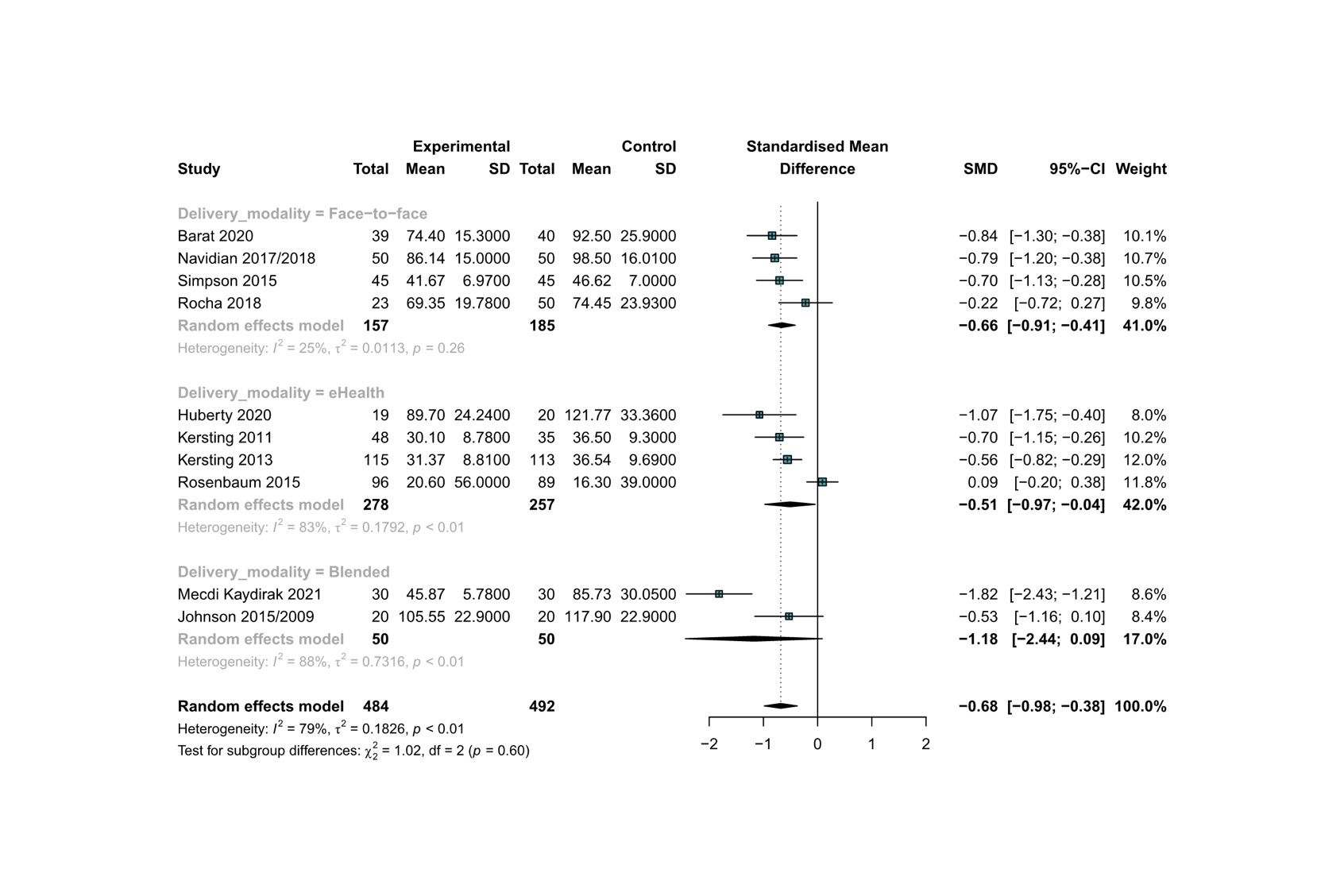


# Supplementary Figure 22. Subgroup analysis to compare the effects of different delivery modalities of the non-pharmacological interventions on parental grief for parents with perinatal loss.


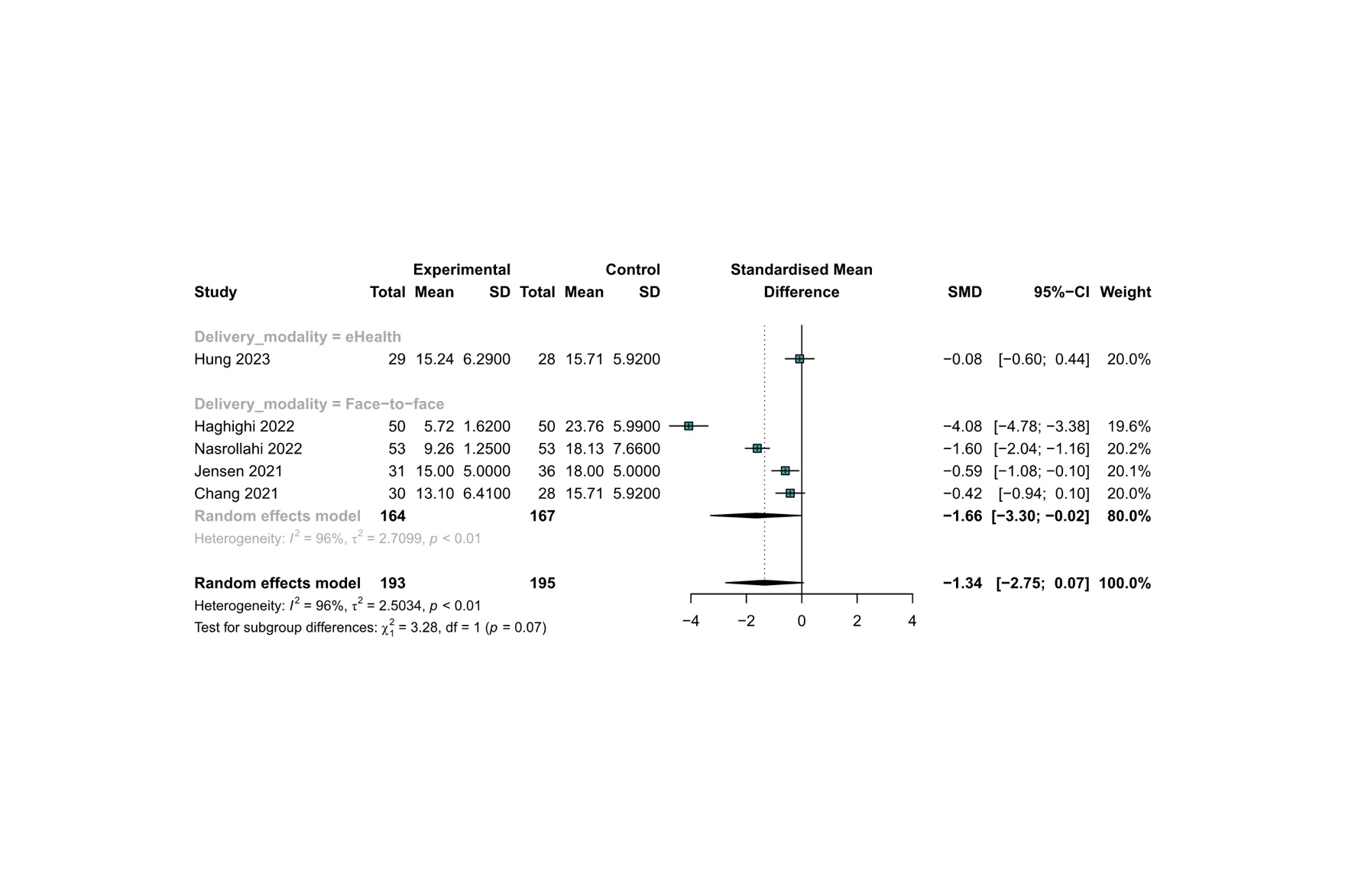


# Supplementary Figure 23. Subgroup analysis to compare the effects of different delivery modalities of the non-pharmacological interventions on parental stress for parents with perinatal loss.


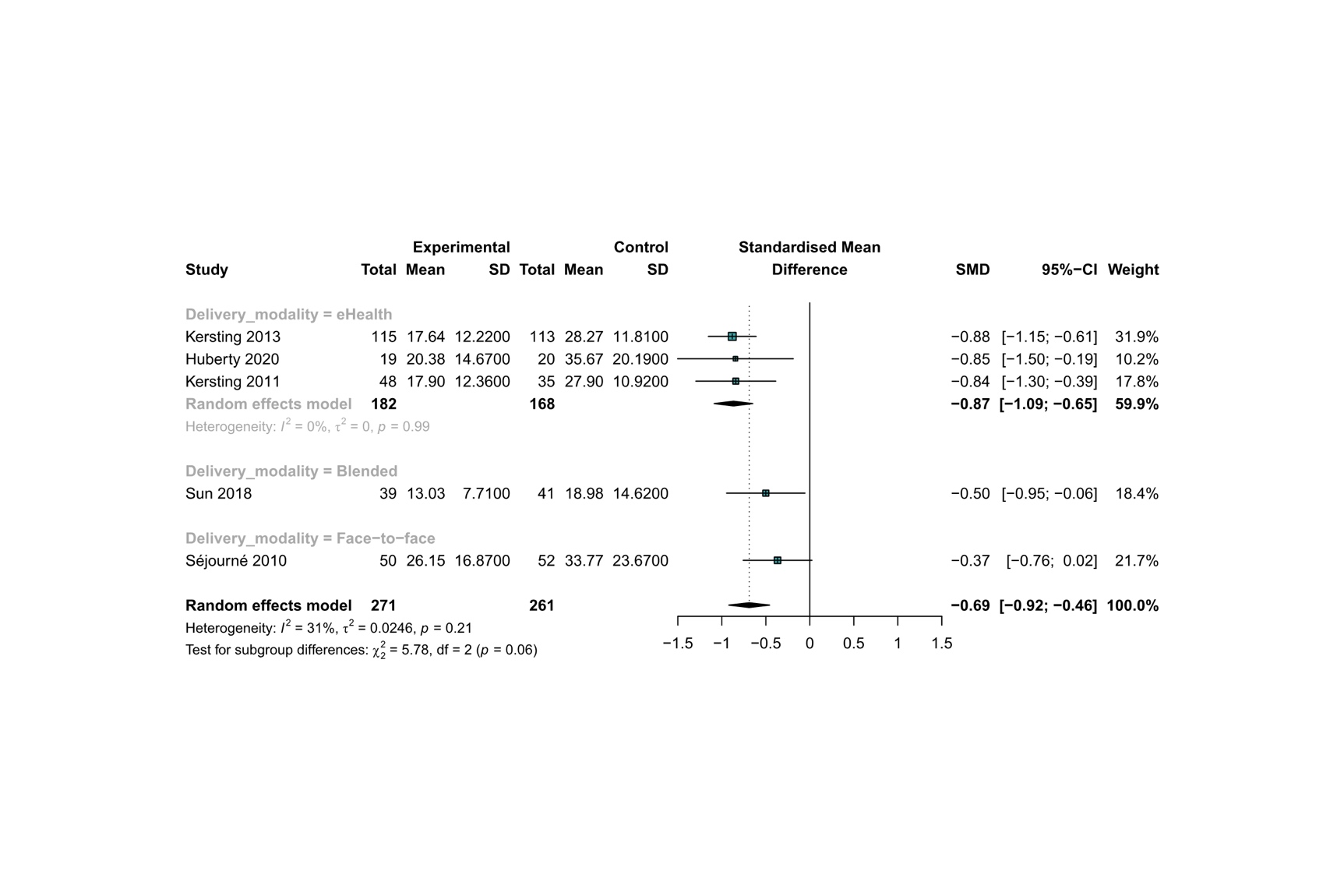


# Supplementary Figure 24. Subgroup analysis to compare the effects of different delivery modalities of the non-pharmacological interventions on parental post-traumatic stress disorder for parents with perinatal loss.


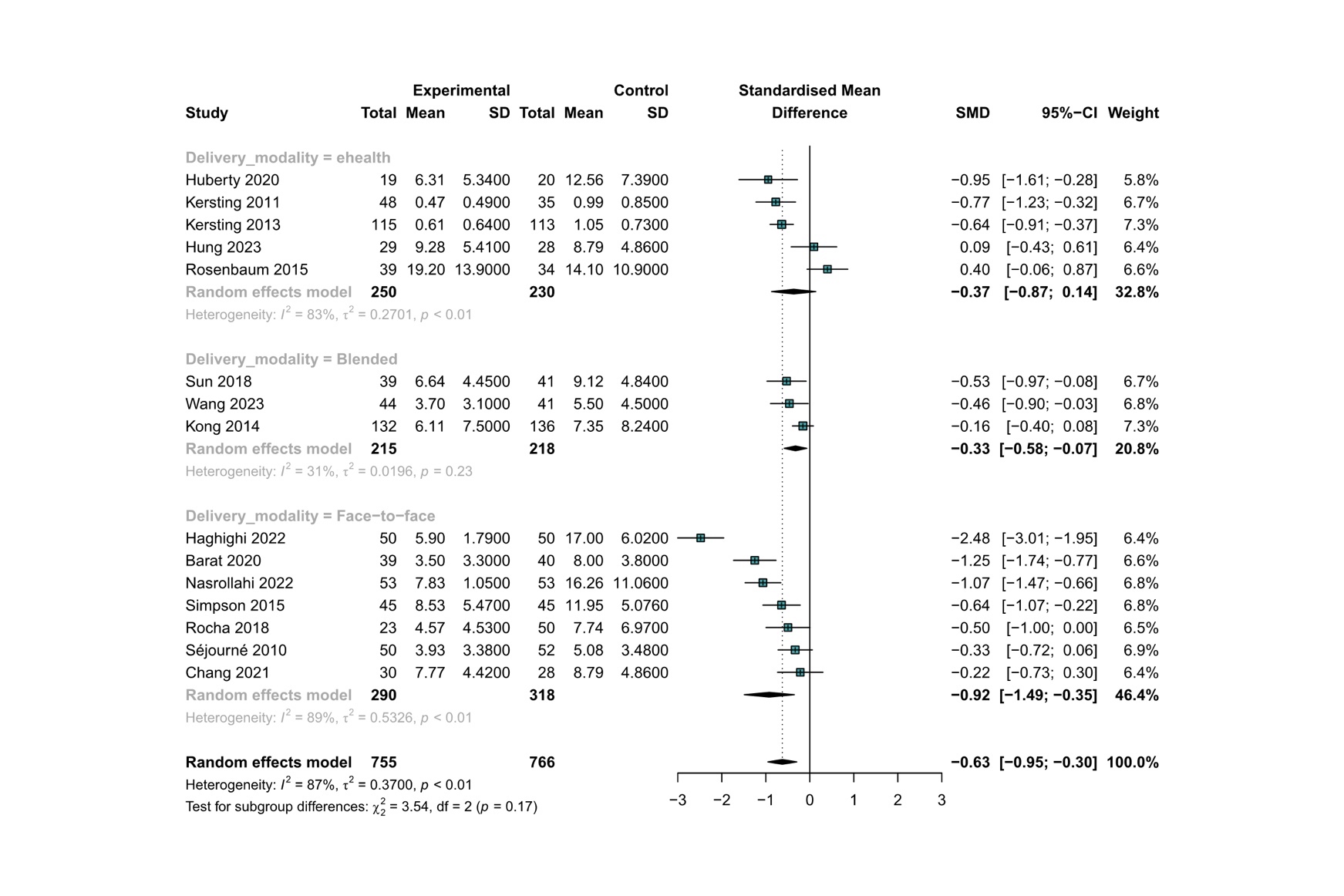


# Supplementary Figure 25. Subgroup analysis to compare the effects of different delivery modalities of the non-pharmacological interventions on parental depression for parents with perinatal loss.


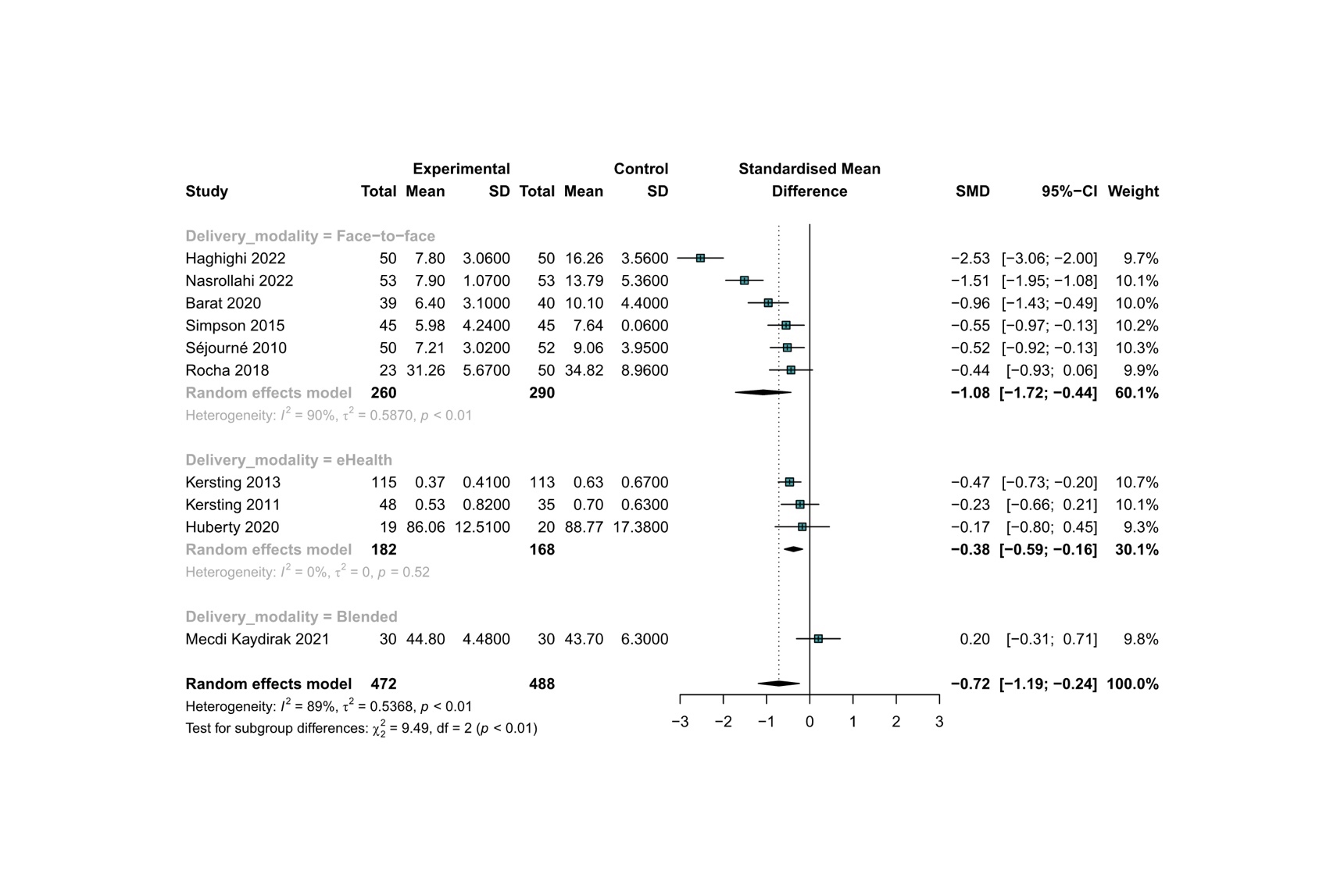


# Supplementary Figure 26. Subgroup analysis to compare the effects of different delivery modalities of the non-pharmacological interventions on parental anxiety for parents with perinatal loss.


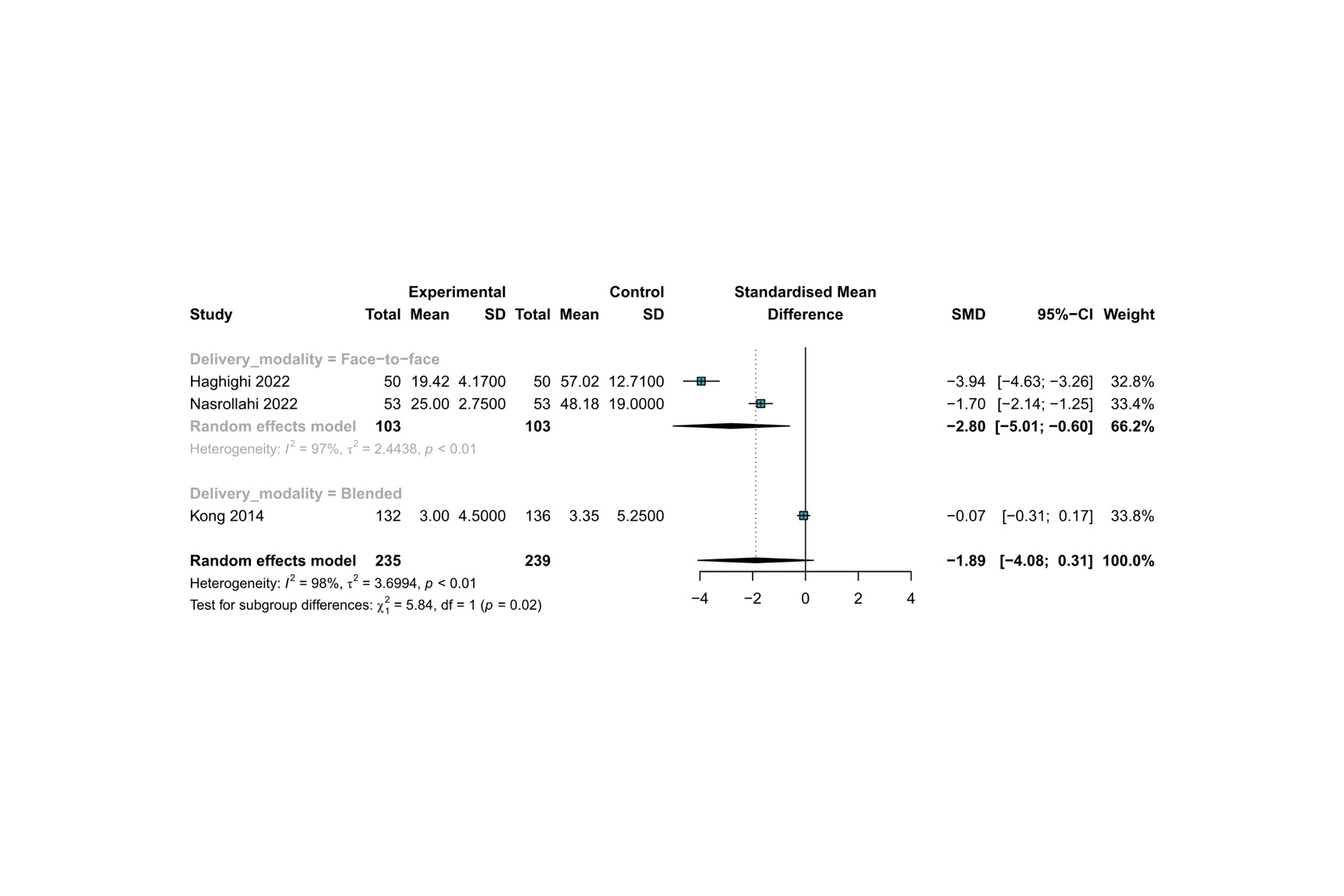


# Supplementary Figure 27. Subgroup analysis to compare the effects of different delivery modalities of the non-pharmacological interventions on parental distress for parents with perinatal loss.


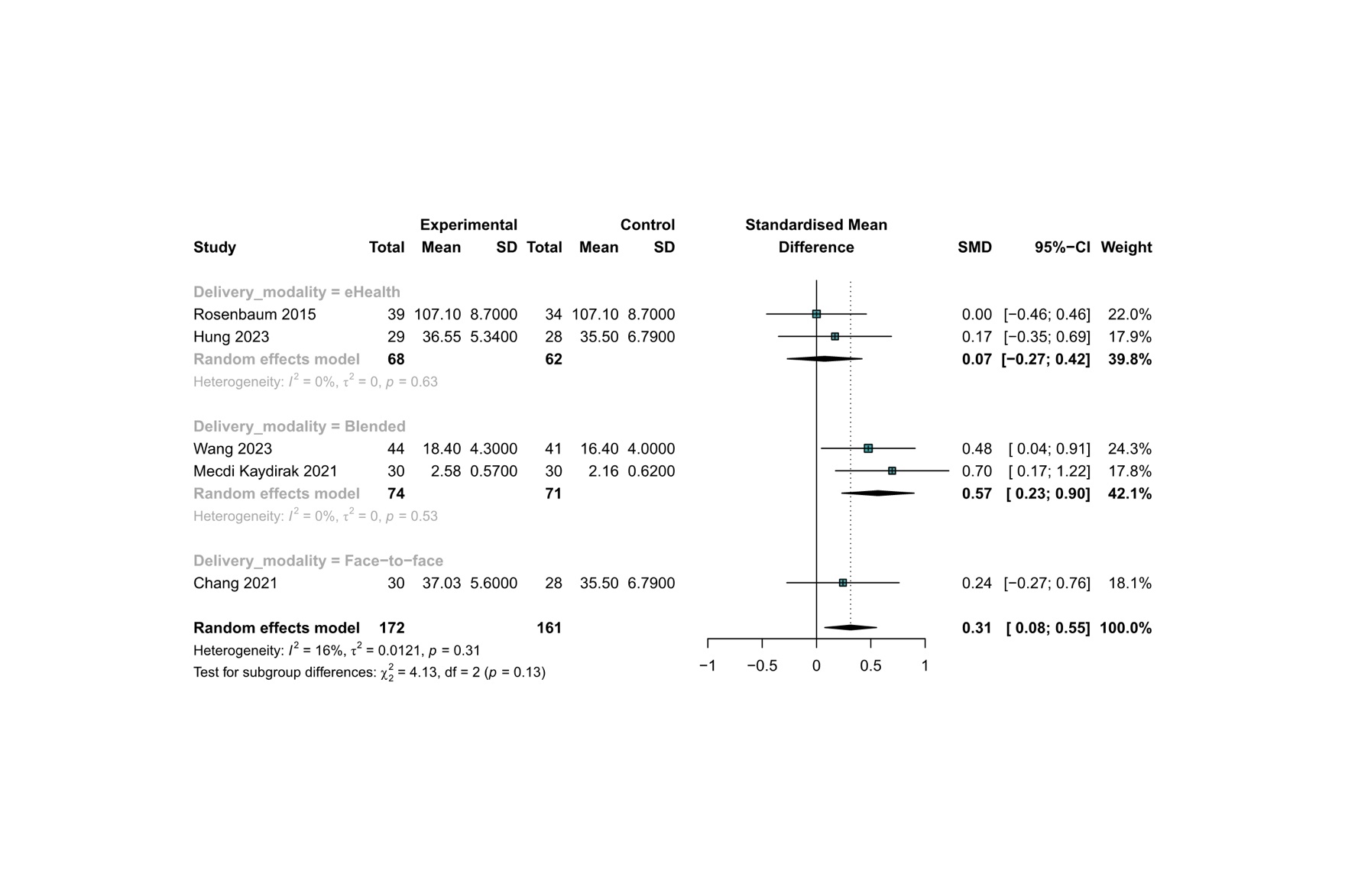


# Supplementary Figure 28. Subgroup analysis to compare the effects of different delivery modalities of the non-pharmacological interventions on perceived social support for parents with perinatal loss.

# Supplementary Table 6. Comparison of treatment effects among different delivery modalities of the non-pharmacological interventions for parents with perinatal loss.

| Outcomes | Face-to-Face | | eHealth | | Blended | |
| --- | --- | --- | --- | --- | --- | --- |
|  | k | Hedges’g (95%CI) | k | Hedges’g (95%CI) | k | Hedges’g (95%CI) |
| Parental grief | 4 | **-0.66 (95% CI: [-0.91, -0.41], P-value for chi-squared test =0.26, I^2^=25%)** | 4 | **-0.51 (95% CI: [-0.97, -0.04], P<0.01, I^2^=83%)** | 2 | -1.18 (95% CI: [-2.44, 0.09], P<0.01, I^2^=88%) |
| Parental stress | 3 | **-1.66 (95% CI: [-3.30, -0.02], P<0.01, I^2^=96%)** | 1 | - | 0 | - |
| Parental post-traumatic stress disorder | 1 | - | 3 | **-0.87 (95% CI: [-1.09, -0.65], P=0.99, I^2^=0%)** | 1 | - |
| Parental depression | 7 | **-0.92 (95% CI: [-1.49, -0.35], P<0.01, I^2^=89%)** | 5 | -0.37 (95% CI: [-0.87, 0.14], P<0.01, I^2^=83%) | 3 | **-0.33 (95% CI: [-0.58, -0.07], P=0.23, I^2^=31%)** |
| Parental anxiety | 6 | **-1.08 (95% CI: [-1.72, -0.44], P<0.01, I^2^=90%)** | 3 | **-0.38 (95% CI: [-0.59, -0.16], P=0.52, I^2^=0%)** | 1 | - |
| Parental distress | 2 | **-2.80 (95% CI: [-5.01, -0.60], P<0.01, I^2^=97%)** | 0 | - | 1 | - |
| Parental sleep quality | 1 | - | 2 | -0.10 (95% CI: [-0.90, -0.69], P=0.05, I^2^=73%) | 1 | - |
| Perceived social support | 1 | - | 2 | 0.07 (95% CI: [-0.27, 0.42], P=0.63, I^2^=0%) | 2 | **0.57 (95% CI: [0.23, 0.90], P=0.53, I^2^=0%)** |

k=number of RCT


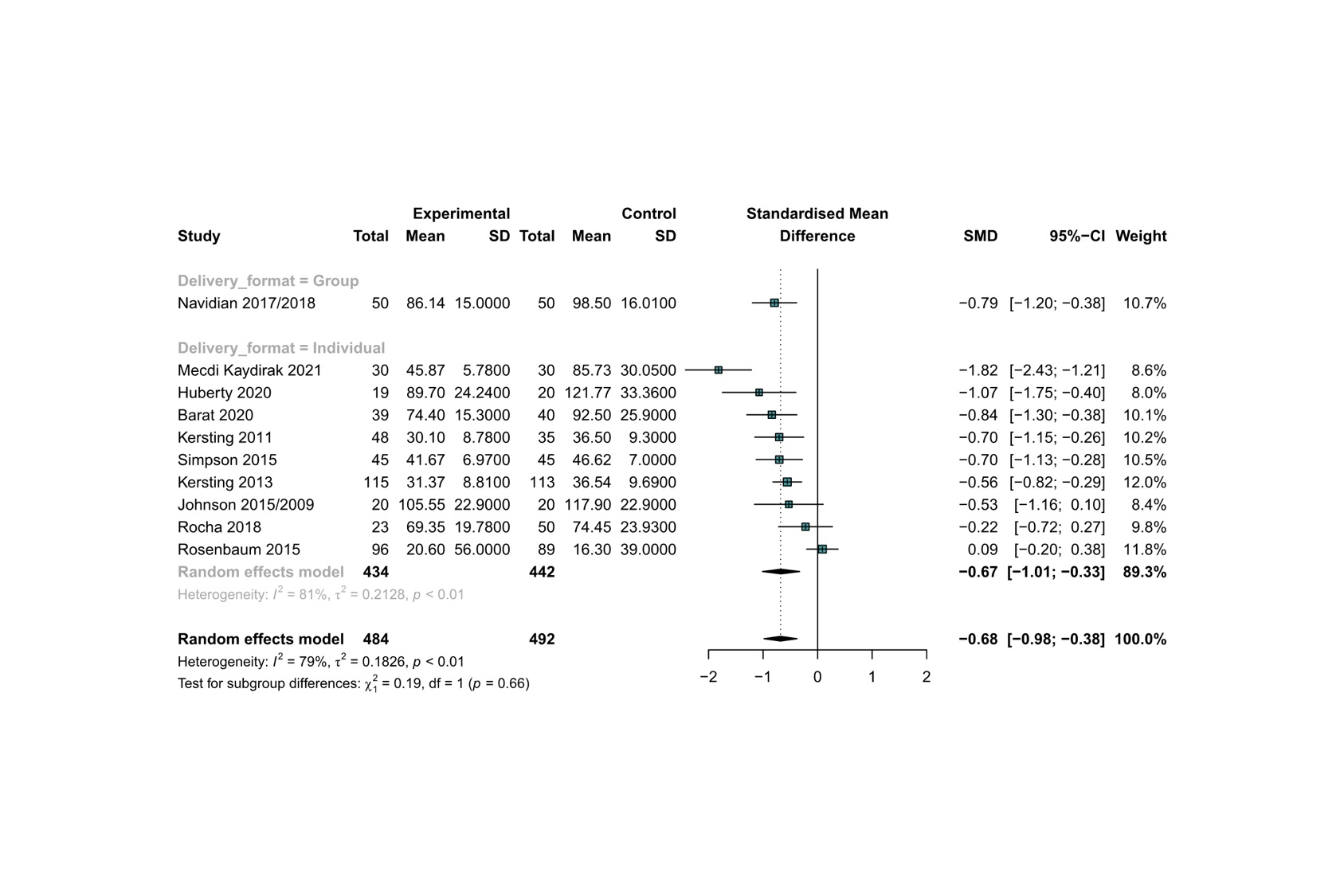


# Supplementary Figure 29. Subgroup analysis to compare the effects of different delivery formats of the non-pharmacological interventions on parental grief for parents with perinatal loss.


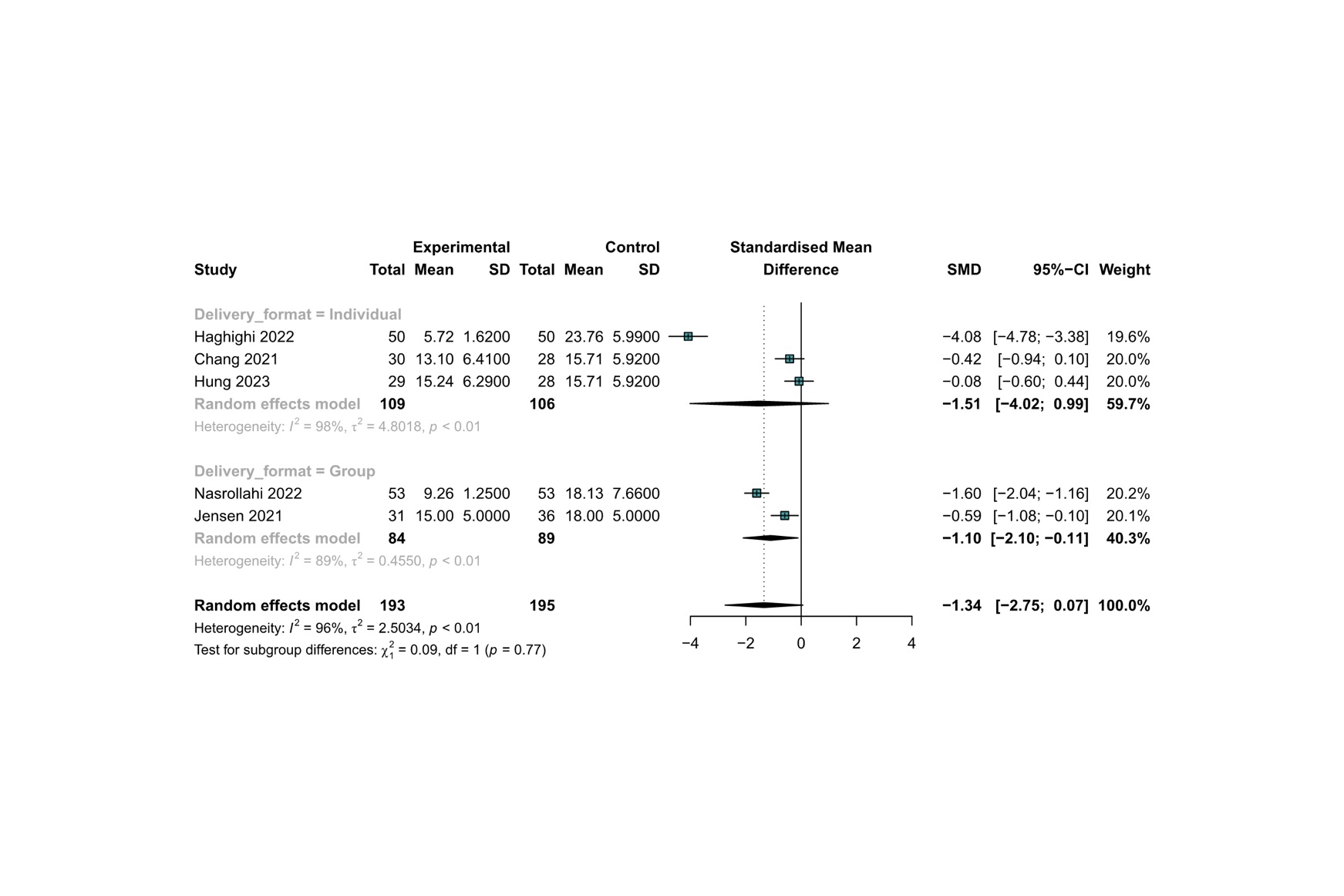


# Supplementary Figure 30. Subgroup analysis to compare the effects of different delivery formats of the non-pharmacological interventions on parental stress for parents with perinatal loss.


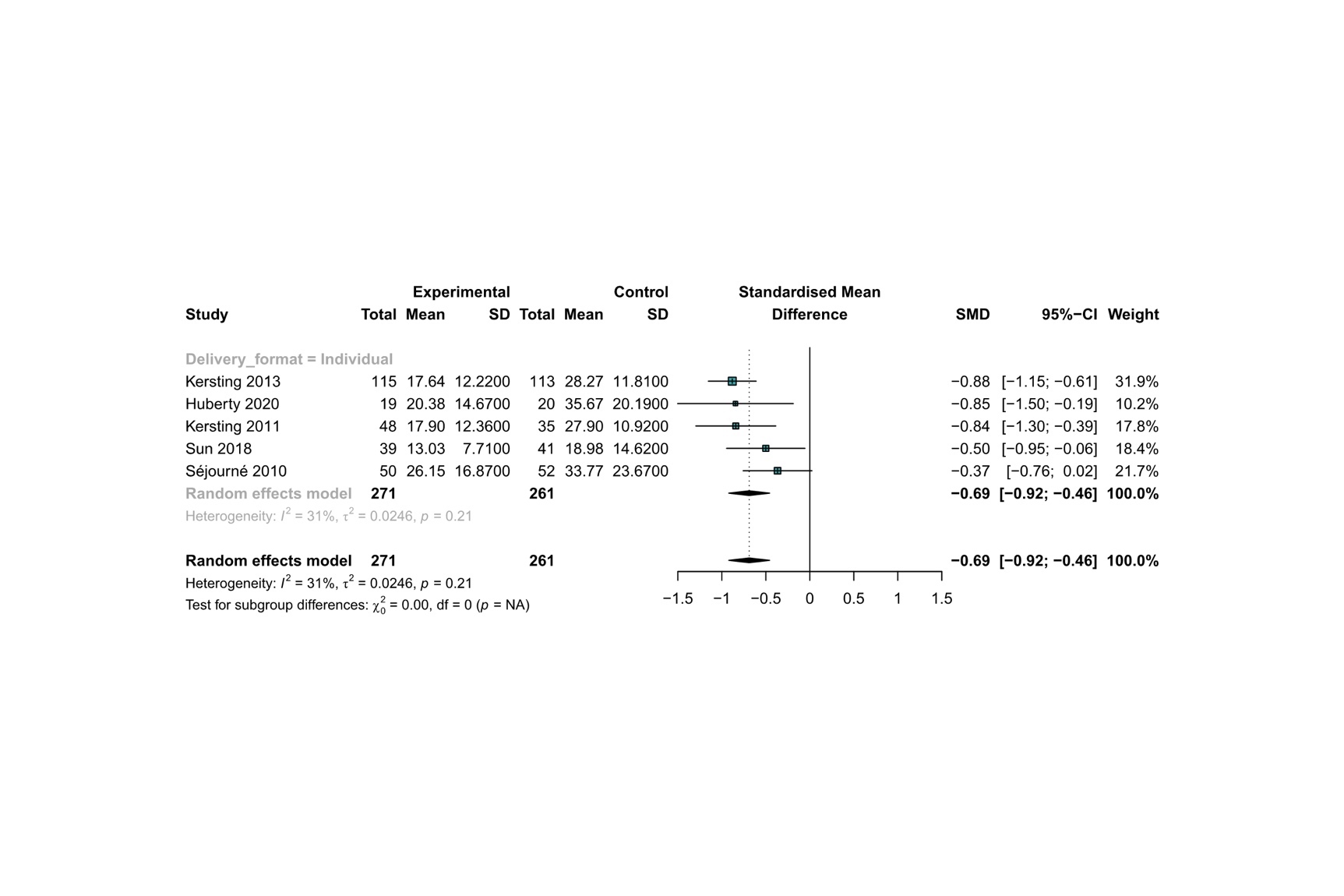


# Supplementary Figure 31. Subgroup analysis to compare the effects of different delivery formats of the non-pharmacological interventions on parental post-traumatic stress disorder for parents with perinatal loss.


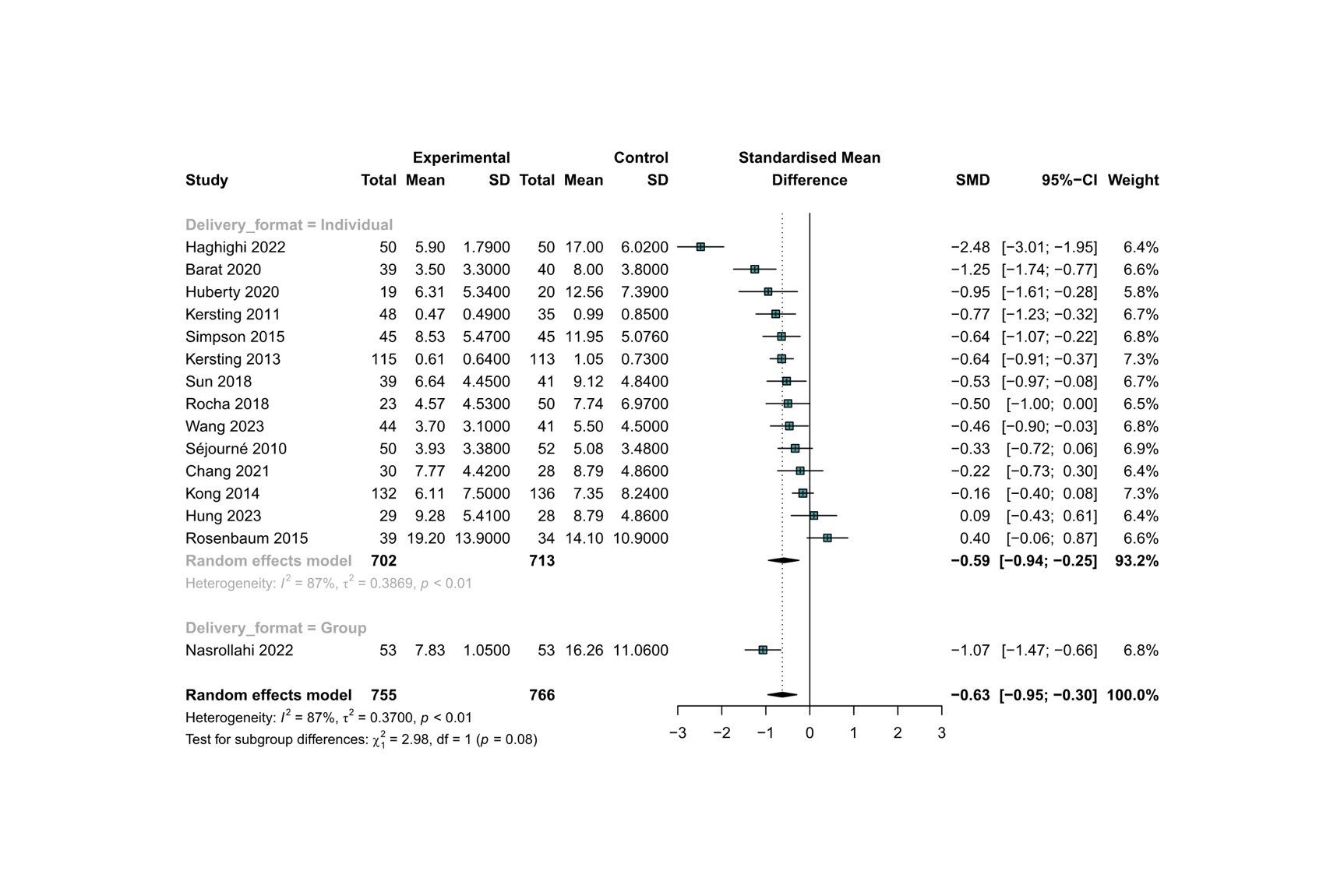


# Supplementary Figure 32. Subgroup analysis to compare the effects of different delivery formats of the non-pharmacological interventions on parental depression for parents with perinatal loss.


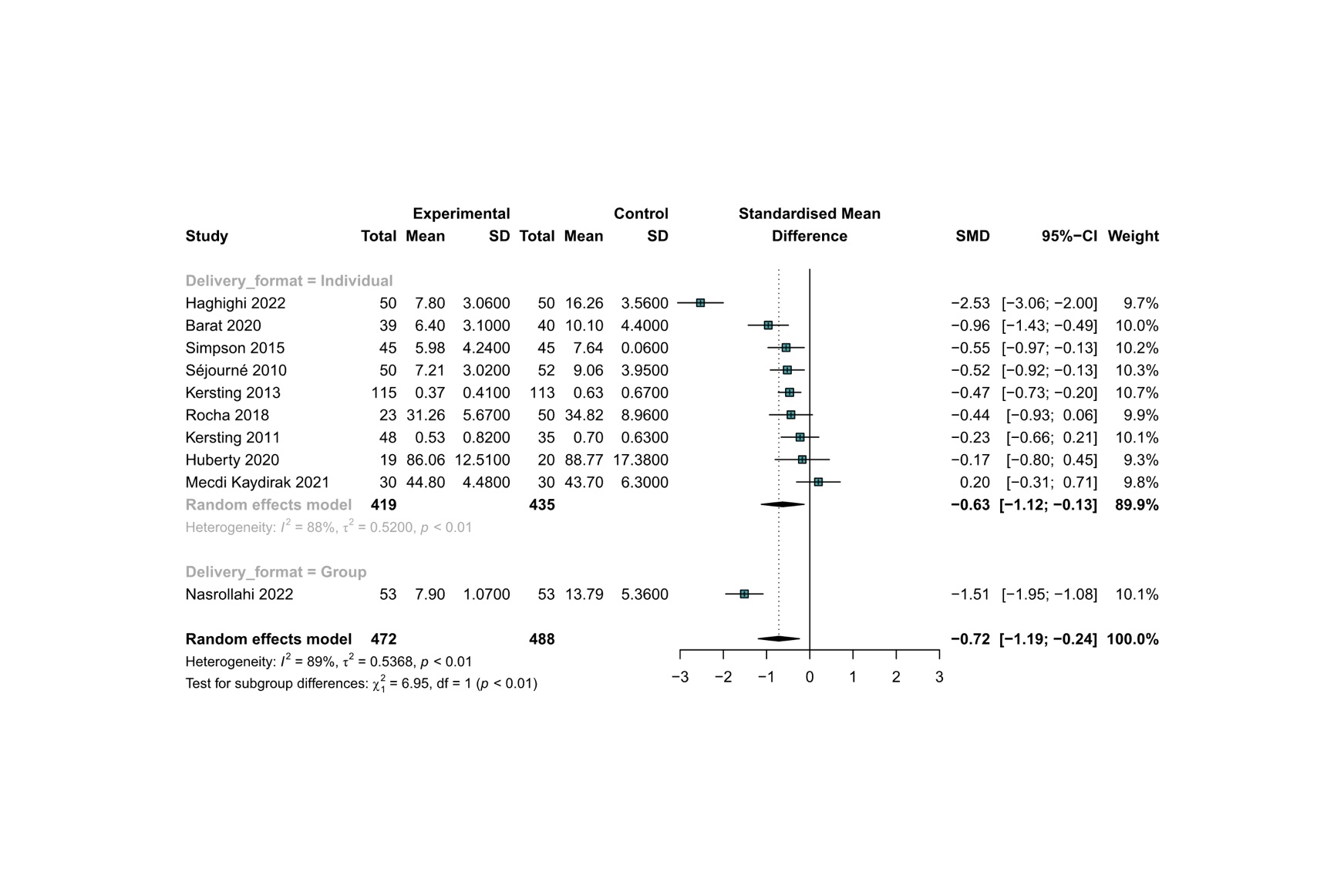


# Supplementary Figure 33. Subgroup analysis to compare the effects of different delivery formats of the non-pharmacological interventions on parental anxiety for parents with perinatal loss.


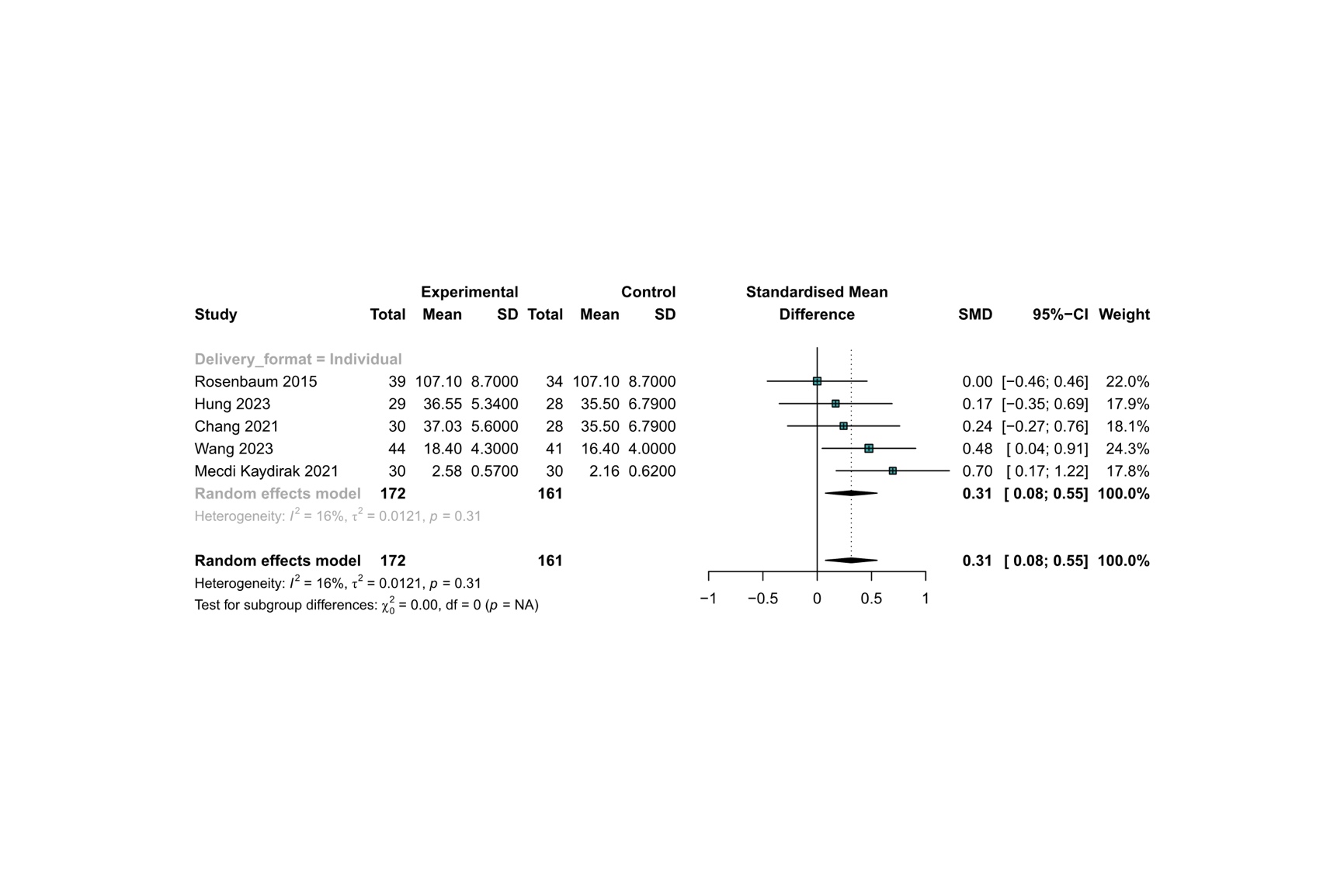


# Supplementary Figure 34. Subgroup analysis to compare the effects of different delivery formats of the non-pharmacological interventions on perceived social support for parents with perinatal loss.

# Supplementary Table 7. Comparison of treatment effects among different delivery formats of the non-pharmacological interventions for parents with perinatal loss.

| Outcomes | Individual | | Group | |
| --- | --- | --- | --- | --- |
|  | k | Hedges’g (95%CI) | k | Hedges’g (95%CI) |
| Parental grief | 9 | **-0.67 (95% CI: [-1.01, -0.33], P-value for chi-squared test <0.01, I^2^=81%)** | 1 | - |
| Parental stress | 3 | -1.51 (95% CI: [-4.02, 0.99], P<0.01, I^2^=98%) | 2 | **-1.10 (95% CI: [-2.10, -0.11], P<0.01, I^2^=89%)** |
| Parental post-traumatic stress disorder | 5 | **-0.69 (95% CI: [-0.92, -0.46], P=0.21, I^2^=31%)** | 0 | - |
| Parental depression | 14 | **-0.59 (95% CI: [-0.94, -0.25], P<0.01, I^2^=87%)** | 1 | - |
| Parental anxiety | 9 | **-0.63 (95% CI: [-1.12, -0.13], P<0.01, I^2^=88%)** | 1 | - |
| Parental distress | 2 | -1.99 (95% CI: [-5.79, 1.80], P<0.01, I^2^=99%) | 1 | - |
| Parental sleep quality | 4 | 0.19 (95% CI: [-0.34, 0.73], P=0.01, I^2^=72%) | 0 | - |
| Perceived social support | 5 | **0.31 (95% CI: [0.08, 0.55], P=0.31, I^2^=16%)** | 0 | - |

k=number of RCT


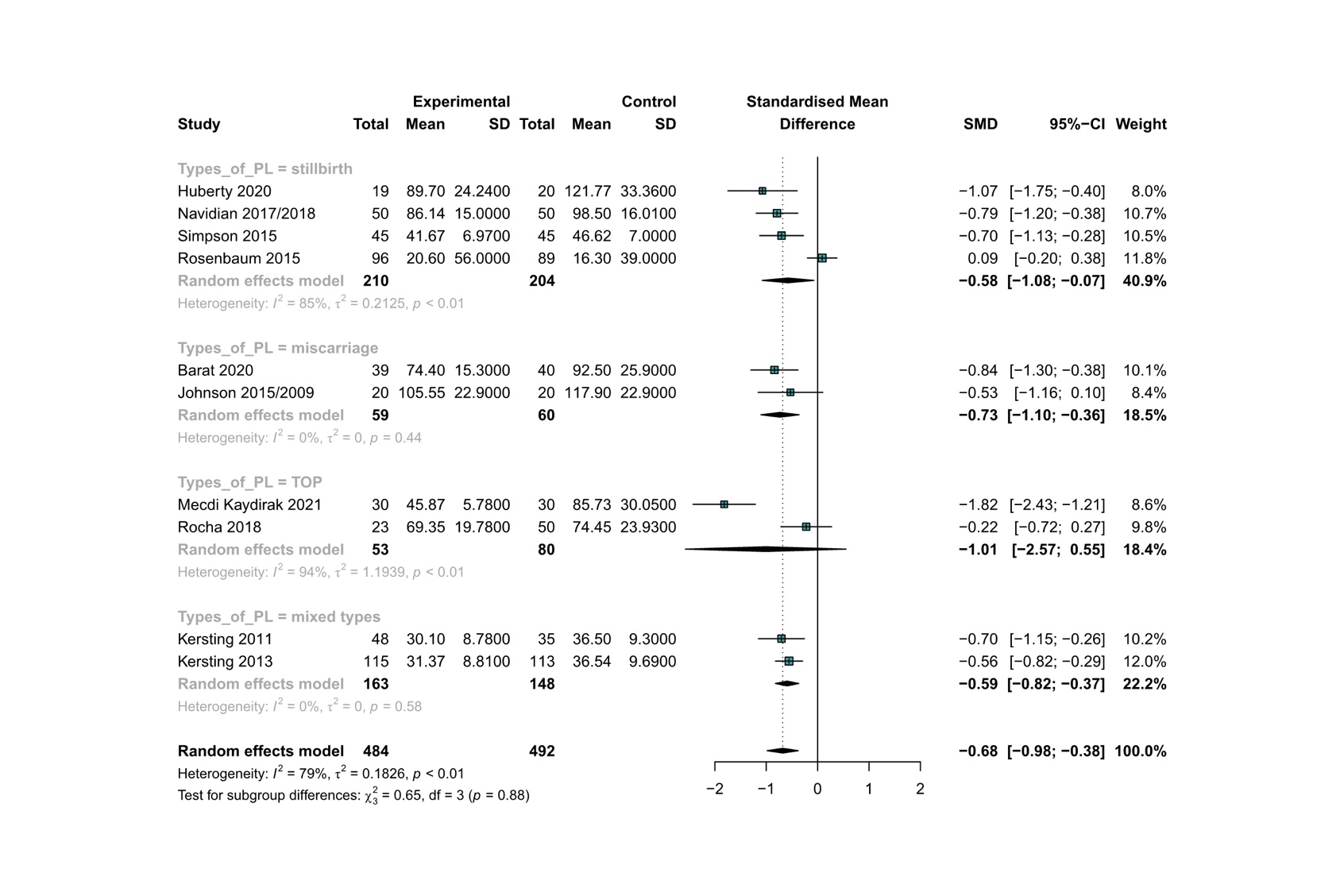


# Supplementary Figure 35. Subgroup analysis to compare the effects of different types of perinatal loss of the non-pharmacological interventions on parental grief for parents with perinatal loss.


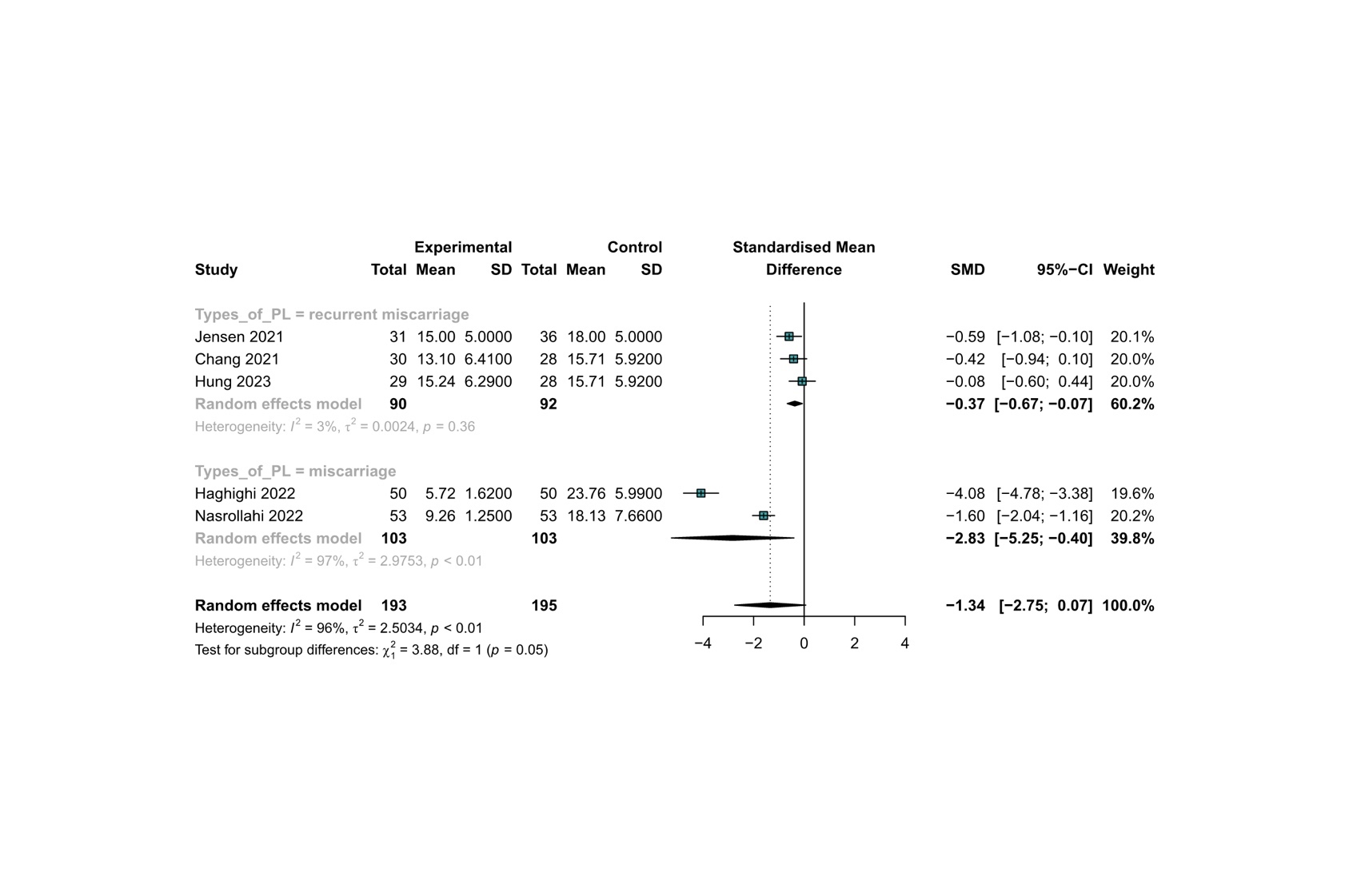


# Supplementary Figure 36. Subgroup analysis for the effects of different types of perinatal loss the non-pharmacological interventions on parental stress for parents with perinatal loss.


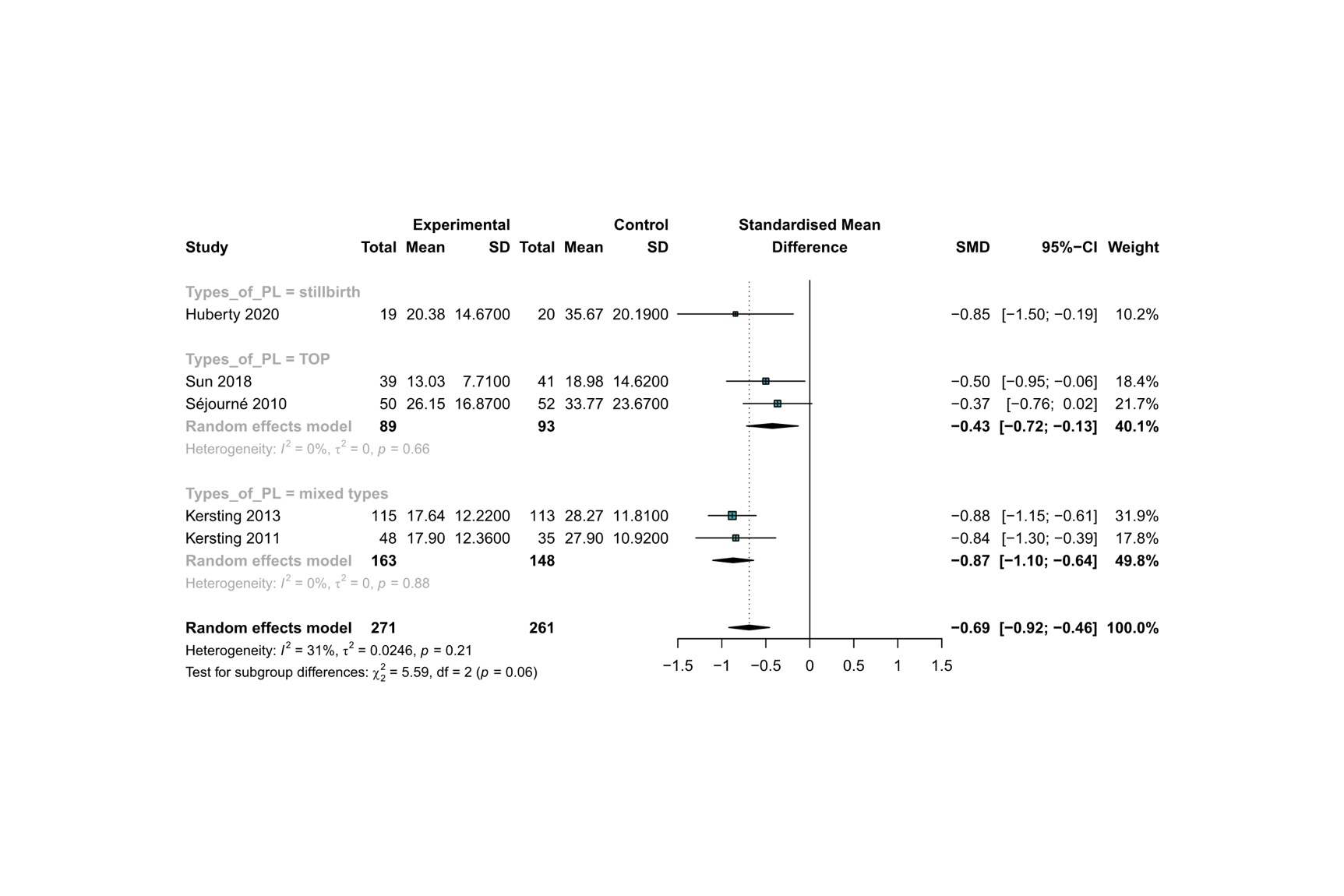


# Supplementary Figure 37. Subgroup analysis for the effects of different types of perinatal loss the non-pharmacological interventions on parental post-traumatic stress disorder for parents with perinatal loss.


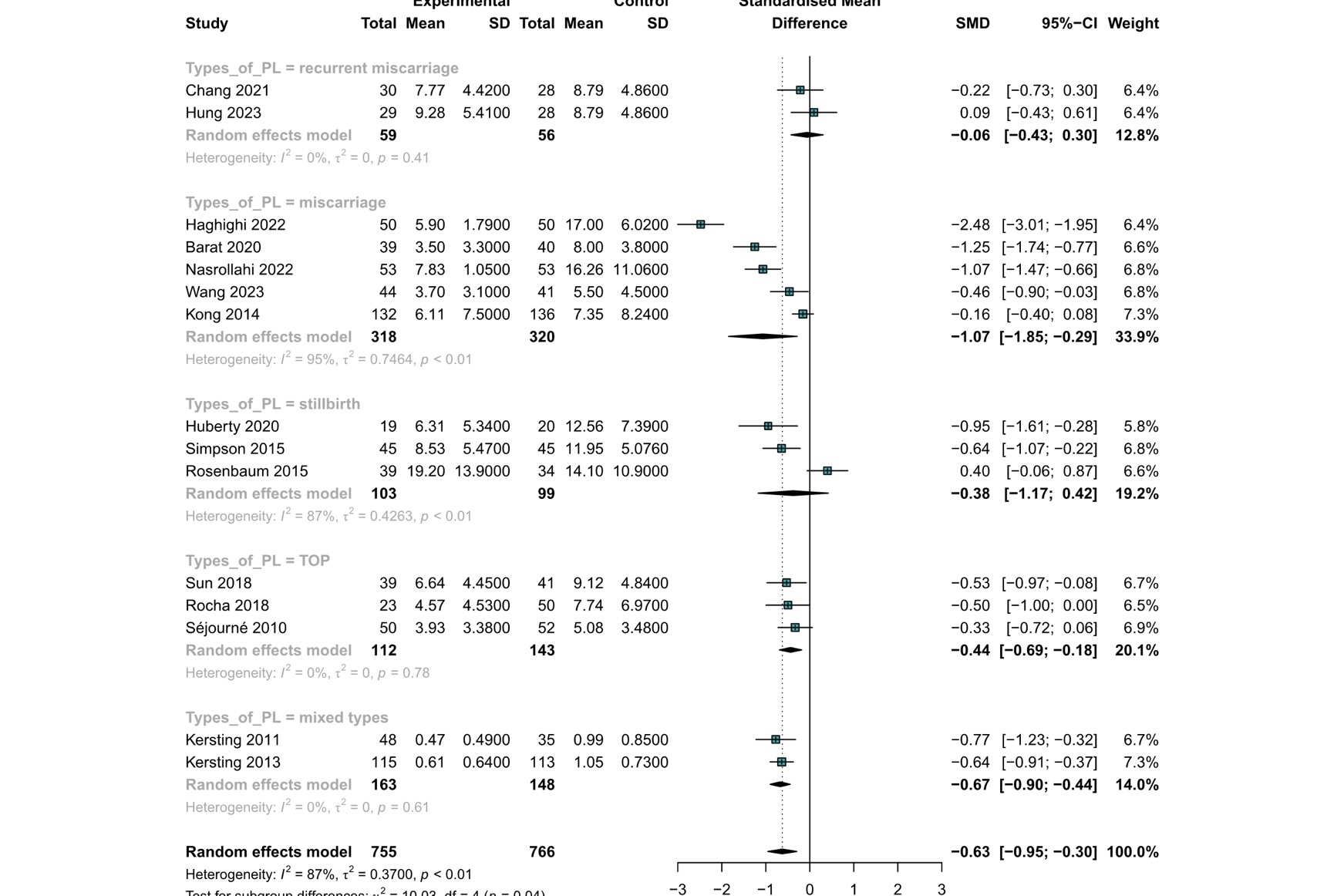


# Supplementary Figure 38. Subgroup analysis for the effects of different types of perinatal loss the non-pharmacological interventions on parental depression for parents with perinatal loss.


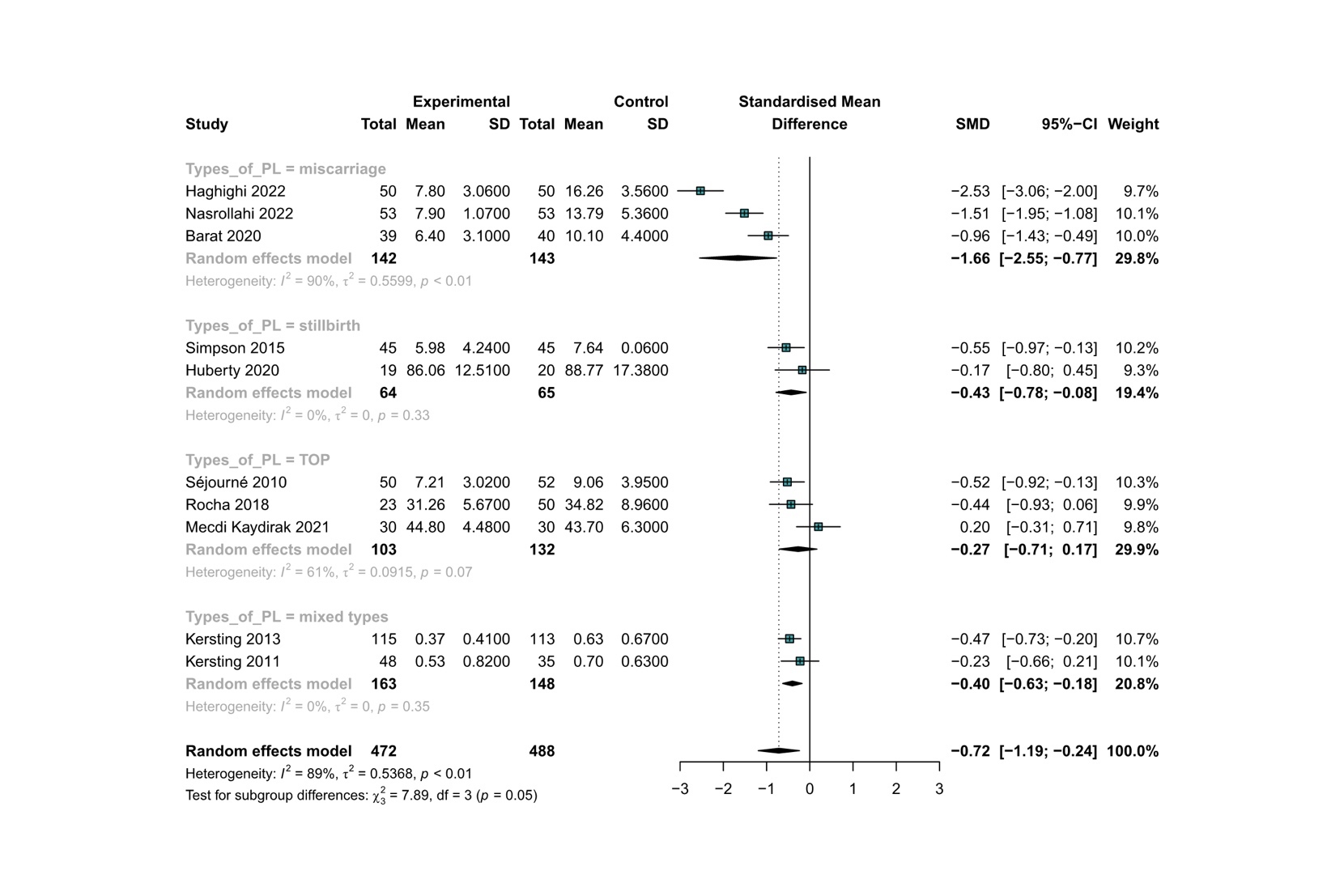


# Supplementary Figure 39. Subgroup analysis for the effects of different types of perinatal loss the non-pharmacological interventions on parental anxiety for parents with perinatal loss.

# Supplementary Table 8. Comparison of treatment effects among different types of perinatal loss of the non-pharmacological interventions for parents with perinatal loss.

| **Outcomes** | **Miscarriage** | | **Stillbirth** | | **TOP** | | **Mixed types** | | **Recurrent miscarriage** | |
| --- | --- | --- | --- | --- | --- | --- | --- | --- | --- | --- |
|  | **k** | **Hedges’g (95%CI)** | **k** | **Hedges’g (95%CI)** | **k** | **Hedges’g (95%CI)** | **k** | **Hedges’g (95%CI)** | **k** | **Hedges’g (95%CI)** |
| **Parental grief** | 2 | **-0.73 (95% CI: [-1.10, -0.36], P-value for chi-squared test =0.14, I^2^=0%)** | 4 | **-0.58 (95% CI: [-1.08, -0.07], P<0.01, I^2^=85%)** | 2 | -1.01 (95% CI: [-2.57, 0.55], P<0.01, I^2^=94%) | 2 | **-0.59 (95% CI: [-0.82, -0.37], P=0.58, I^2^=0%)** | 0 | - |
| **Parental stress** | 2 | **-2.83 (95% CI: [-5.25, -0.40], P<0.01, I^2^=97%)** | 0 | - | 0 | - | 0 | - | **3** | **-0.37 (95% CI: [-0.67, -0.07], P=0.36, I^2^=3%)** |
| **Parental post-traumatic stress disorder** | 0 | - | 1 | - | 2 | **-0.43 (95% CI: [-0.72, -0.13], P=0.66, I^2^=0%)** | 2 | **-0.87 (95% CI: [-1.10, -0.64], P=0.88, I^2^=0%)** | 0 | - |
| **Parental depression** | 5 | **-1.07 (95% CI: [-1.85, -0.29], P<0.01, I^2^=95%)** | 3 | -0.38 (95% CI: [-1.17, 0.42], P<0.01, I^2^=87%) | 3 | **-0.44 (95% CI: [-0.69, -0.18], P=0.78, I^2^=0%)** | 2 | **-0.67 (95% CI: [-0.90, -0.44], P=0.61, I^2^=0%)** | 2 | -0.06 (95% CI: [-0.43, 0.30], P=0.41, I^2^=0%) |
| **Parental anxiety** | 3 | **-1.66 (95% CI: [-2.55, -0.77], P<0.01, I^2^=90%)** | 2 | **-0.43 (95% CI: [-0.78, -0.08], P=0.33, I^2^=0%)** | 3 | -0.27 (95% CI: [-0.71, 0.17], P=0.07, I^2^=61%) | 2 | **-0.40 (95% CI: [-0.63, -0.18], P=0.35, I^2^=0%)** | 0 | - |
| **Parental distress** | 3 | -1.89 (95% CI: [-4.08, 0.31], P<0.01, I^2^=98%) | 0 | - | 0 | - | 0 | - | 0 | - |
| **Parental sleep quality** | 0 | - | 1 | - | 1 | - | 0 | - | 2 | 0.19 (95% CI: [-0.17, 0.56], P=0.64, I^2^=0%) |
| **Perceived social support** | 1 | - | 1 | - | 1 | - | 0 | - | 2 | 0.21 (95% CI: [-0.16, 0.57], P=0.84, I^2^=0%) |

k=number of RCT; TOP=termination of pregnancy due to detection of fetal health problems or fetal abnormality.


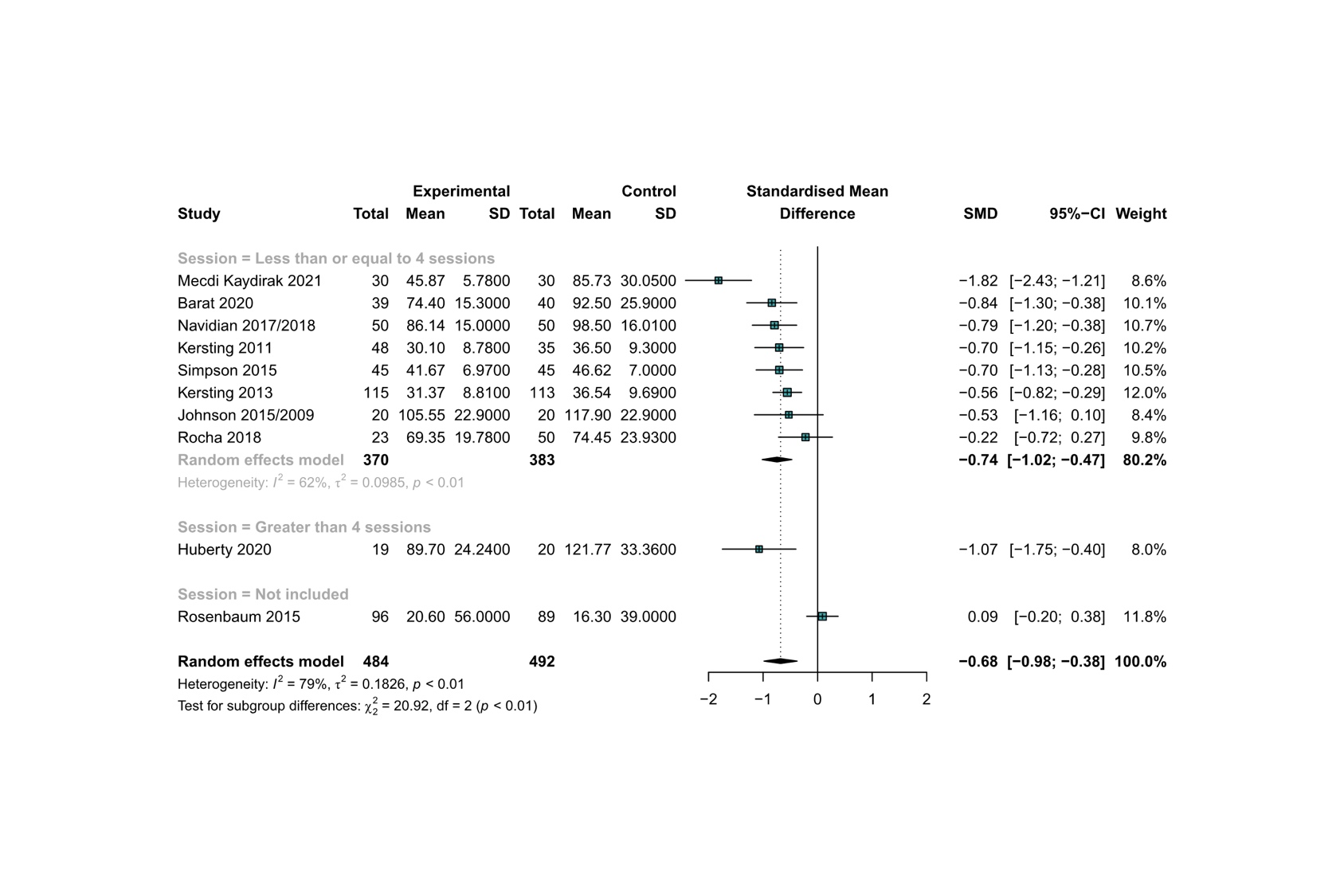


# Supplementary Figure 40. Subgroup analysis for the effects of different intervention sessions the non-pharmacological interventions on parental grief for parents with perinatal loss.


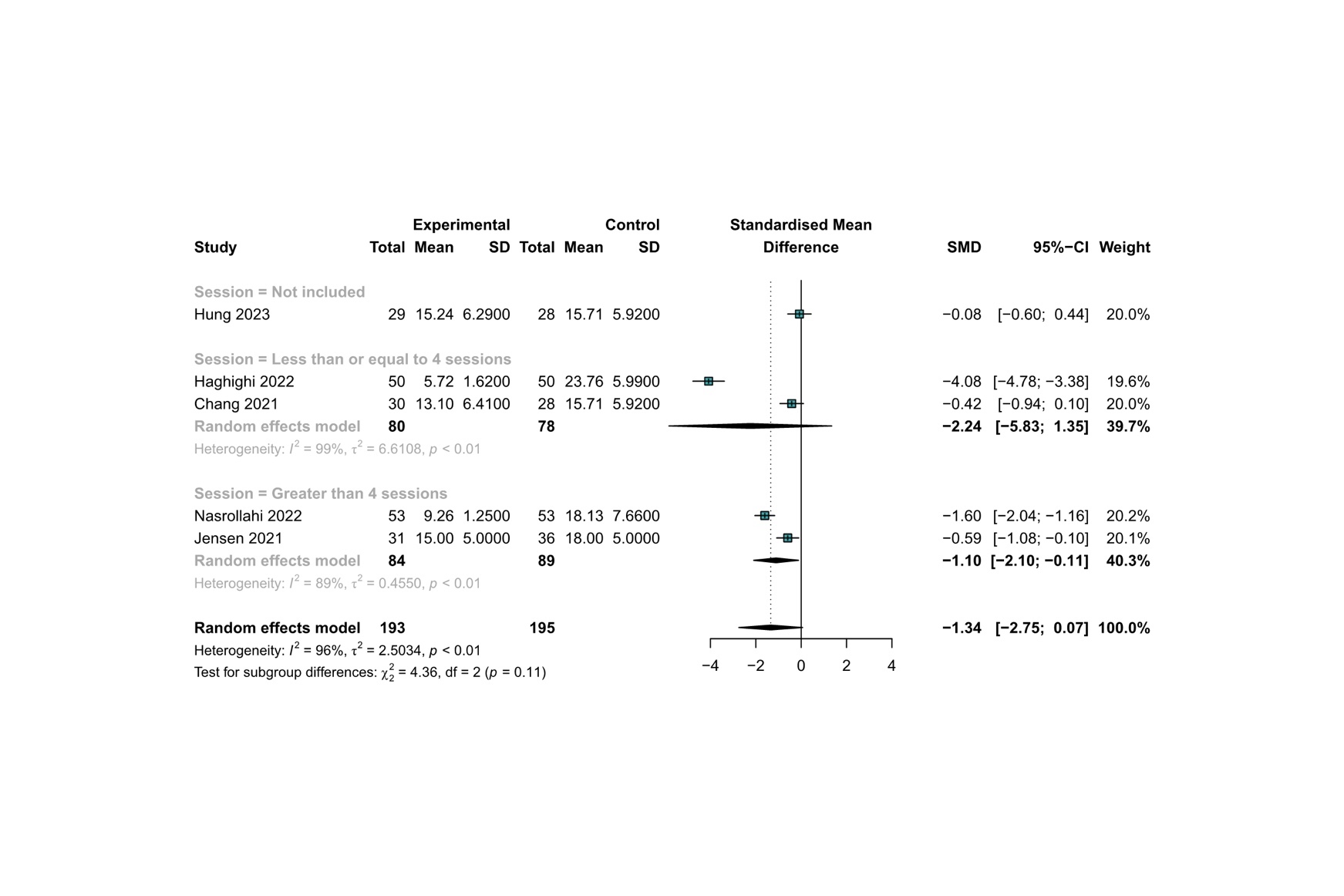


# Supplementary Figure 41. Subgroup analysis for the effects of different intervention sessions the non-pharmacological interventions on parental stress for parents with perinatal loss.


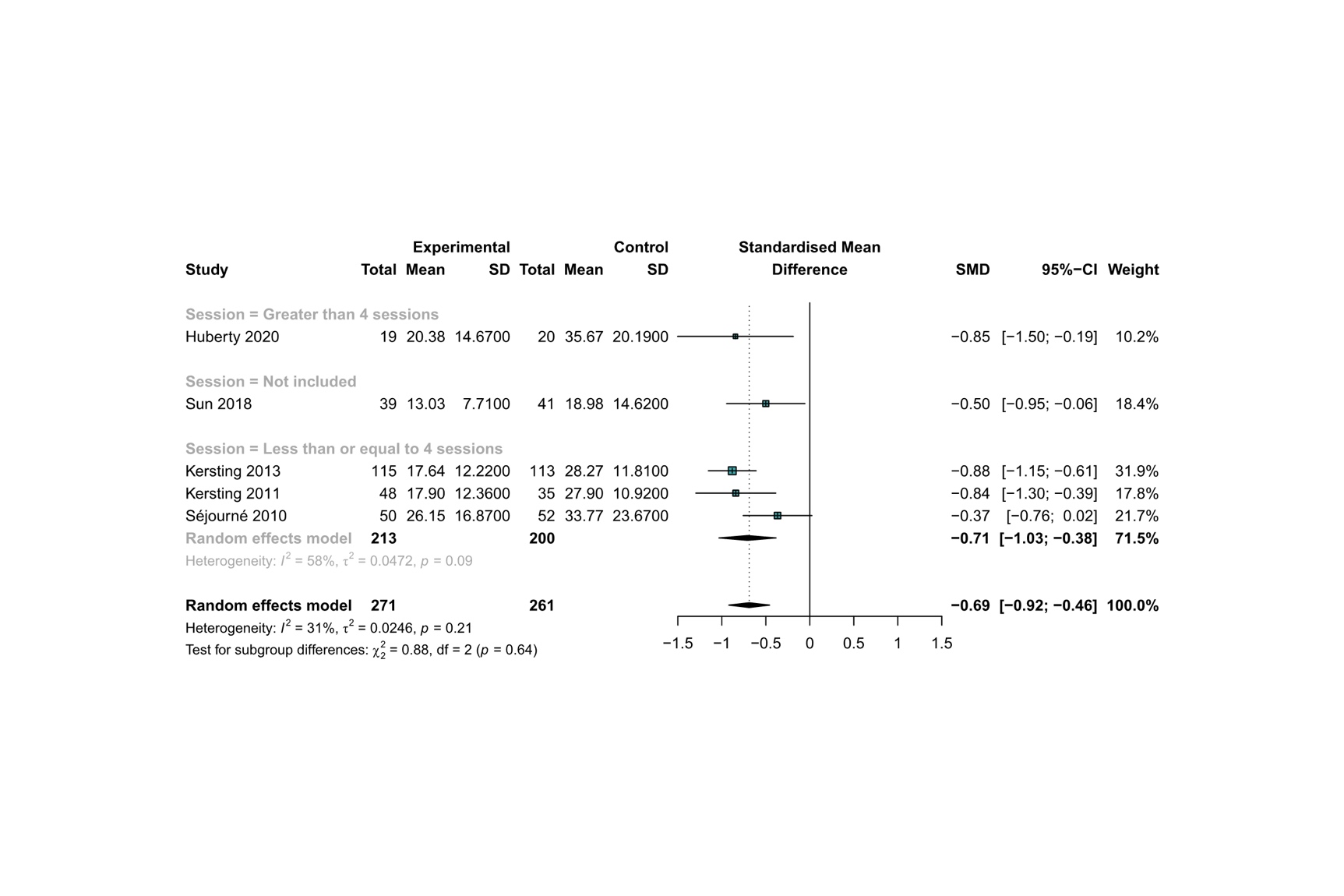


# Supplementary Figure 42. Subgroup analysis for the effects of different intervention sessions the non-pharmacological interventions on parental post-traumatic stress disorder for parents with perinatal loss.


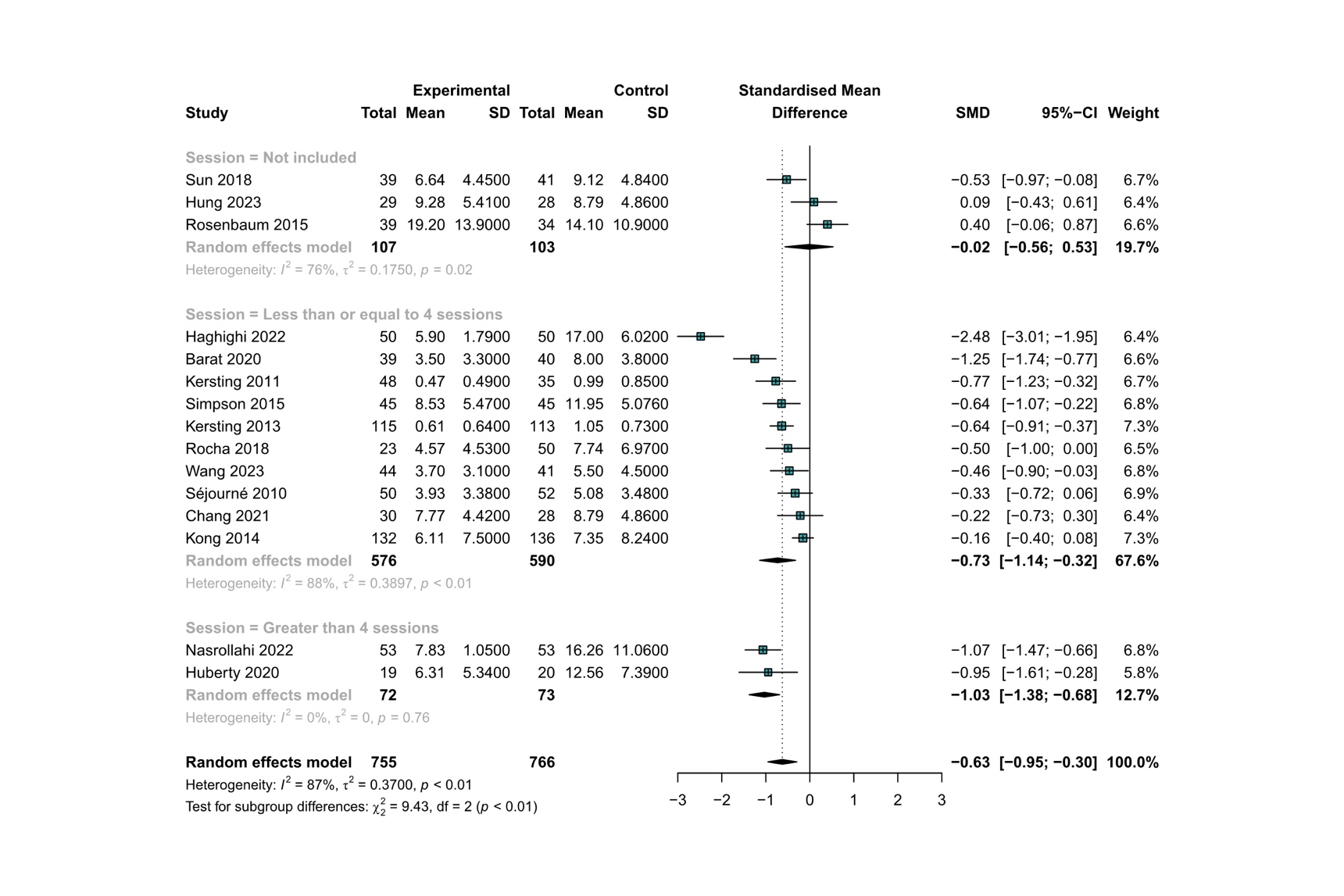


# Supplementary Figure 43. Subgroup analysis for the effects of different intervention sessions the non-pharmacological interventions on parental depression for parents with perinatal loss.


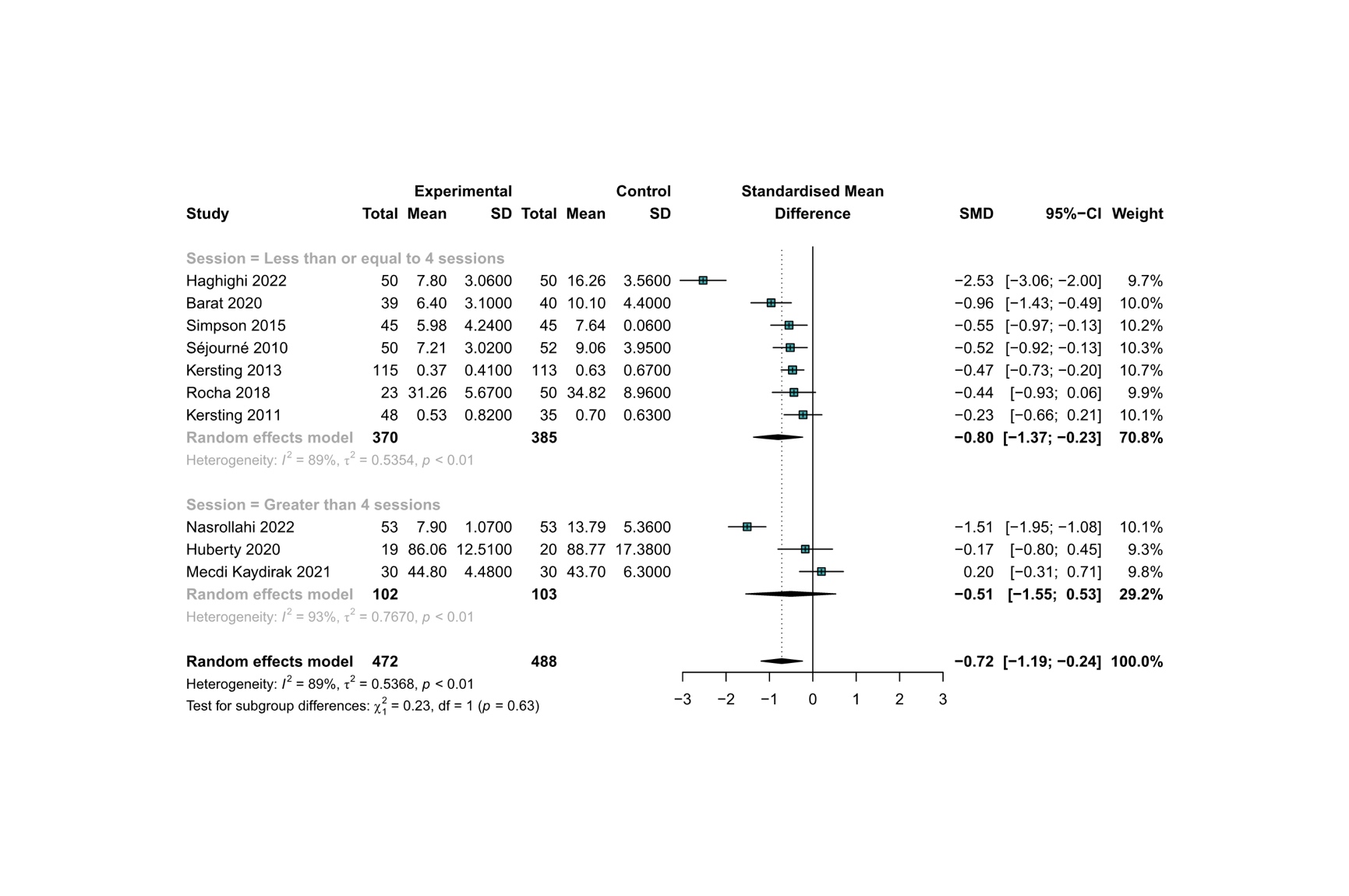


# Supplementary Figure 44. Subgroup analysis for the effects of different intervention sessions the non-pharmacological interventions on parental anxiety for parents with perinatal loss.


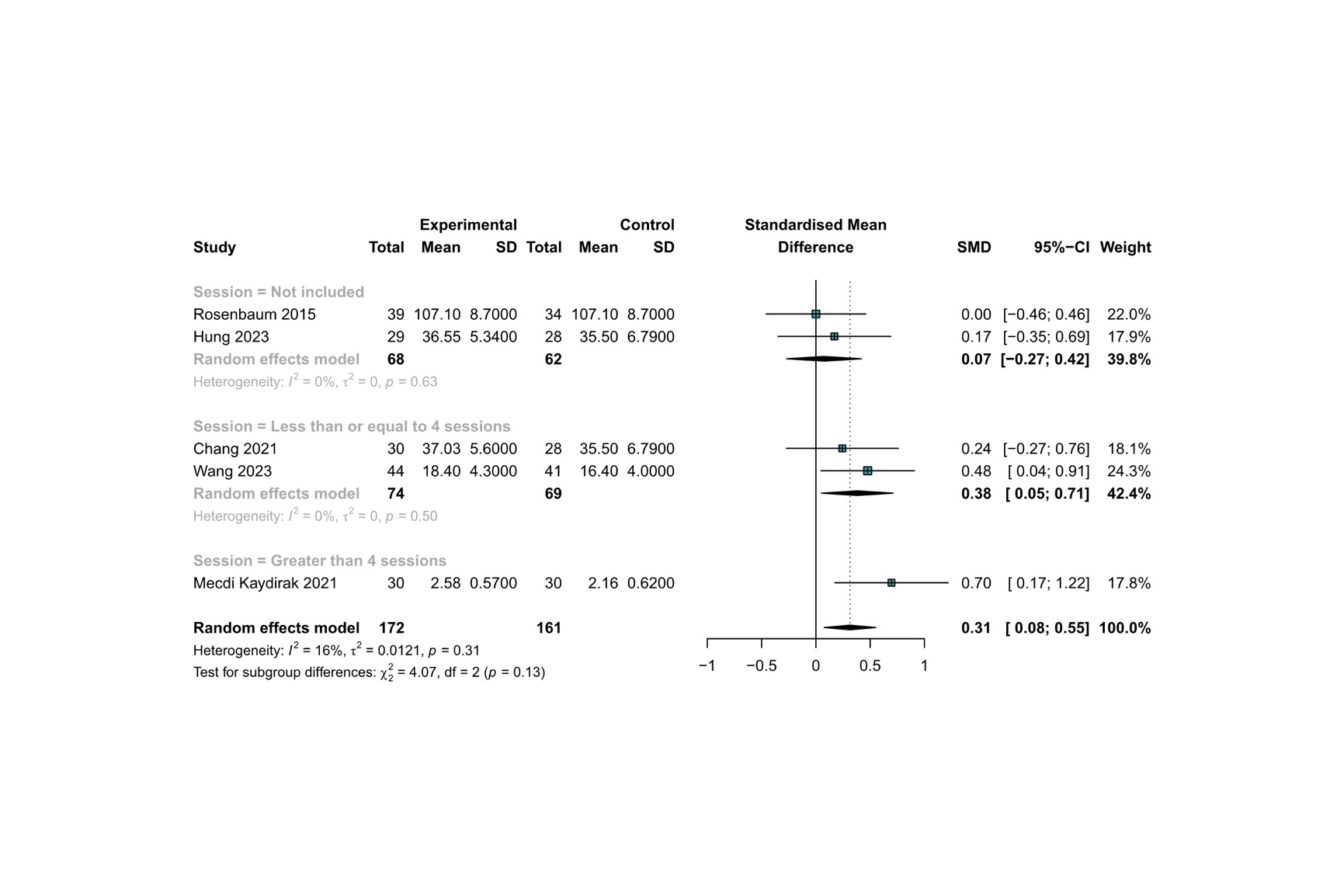


# Supplementary Figure 45. Subgroup analysis for the effects of different intervention sessions the non-pharmacological interventions on perceived social support for parents with perinatal loss.

# Supplementary Table 9. Comparison of treatment effects among different intervention sessions of the non-pharmacological interventions for parents with perinatal loss.

| **Outcomes** | **Less than or equal to 4 sessions** | | **Greater than 4 sessions** | |
| --- | --- | --- | --- | --- |
|  | **k** | **Hedges’g (95%CI)** | **k** | **Hedges’g (95%CI)** |
| **Parental grief** | 8 | **-0.74 (95% CI: [-1.02, -0.47], P-value for chi-squared test <0.01, I^2^=62%)** | 1 | - |
| **Parental stress** | 2 | **-1.10 (95% CI: [-2.10, -0.11], P<0.01, I^2^=89%)** | 2 | -2.24 (95% CI: [-5.83, 1.35], P<0.01, I^2^=99%) |
| **Parental post-traumatic stress disorder** | 3 | **-0.71 (95% CI: [-1.03, -0.38], P=0.09, I^2^=58%)** | 1 | **-** |
| **Parental depression** | 10 | **-0.74 (95% CI: [-1.14, -0.32], P<0.01, I^2^=88%)** | 2 | **-1.03 (95% CI: [-1.38, -0.68], P=0.76, I^2^=0%)** |
| **Parental anxiety** | 5 | **-0.80 (95% CI: [-1.37, -0.23], P<0.01, I^2^=89%)** | 3 | -0.51 (95% CI: [-1.55, 0.53], P<0.01, I^2^=93%) |
| **Parental distress** | 2 | -1.99 (95% CI: [-5.79, 1.80], P<0.01, I^2^=99%) | 1 | - |
| **Parental sleep quality** | 1 | - | 2 | 0.17 (95% CI: [-1.18, 1.51], P<0.01, I^2^=90%) |
| **Perceived social support** | 2 | **0.38 (95% CI: [0.05, 0.71], P=0.50, I^2^=0%)** | 1 | - |

k=number of RCT

# Reference

Antill, J. K., & Cotton, S. (1982). Spanier's dyadic adjustment scale: Some confirmatory analyses. *Australian Psychologist, 17*(2), 181-189.

Barat, S., Yazdani, S., Faramarzi, M., Khafri, S., Darvish, M., Rad, M. N., & Asnafi, N. (2020). The Effect of Brief Supportive Psychotherapy on Prevention of Psychiatric Morbidity in Women with Miscarriage: A Randomized Controlled Trial about the First 24-hours of Hospitalization. *Oman Med J, 35*(3), e130. doi:10.5001/omj.2020.48

Beck, A., Steer, R., & Brown, G. J. T. T. P. A. (1996). Manual for the Beck Depression Inventory, (BDI-II) San Antonio.

Beck, A. T., Ward, C., Mendelson, M., Mock, J., & Erbaugh, J. (1961). Beck depression inventory (BDI). *Arch Gen Psychiatry, 4*(6), 561-571.

Buysse, D. J., Reynolds III, C. F., Monk, T. H., Berman, S. R., & Kupfer, D. J. (1989). The Pittsburgh Sleep Quality Index: a new instrument for psychiatric practice and research. *Psychiatry research, 28*(2), 193-213.

Chang, S. C., Kuo, P. L., & Chen, C. H. (2021). Effectiveness of empathic caring on stress and depression for women with recurrent miscarriage: A randomized controlled trial. *Complement Ther Clin Pract, 43*, 101367. doi:10.1016/j.ctcp.2021.101367

Cohen, S., & Hoberman, H. (1983). Interpersonal support evaluation list (ISEL). *Journal of Applied Social Psychology, 13*(1), 99-125.

Cohen, S., Kamarck, T., & Mermelstein, R. (1983). A global measure of perceived stress. *Journal of health and social behavior*, 385-396.

Cox, J. L., Holden, J. M., & Sagovsky, R. (1987). Detection of postnatal depression: development of the 10-item Edinburgh Postnatal Depression Scale. *The British Journal of Psychiatry, 150*(6), 782-786.

Creamer, M., Bell, R., & Failla, S. (2003). Psychometric properties of the impact of event scale—revised. *Behav Res Ther, 41*(12), 1489-1496.

Cutrona, C. E. (1986). Objective determinants of perceived social support. *Journal of personality and social psychology, 50*(2), 349.

Dahlem, N. W., Zimet, G. D., & Walker, R. R. (1991). The multidimensional scale of perceived social support: a confirmation study. *Journal of clinical psychology, 47*(6), 756-761.

Derogatis, L. R. (1975). Brief symptom inventory. *European Journal of Psychological Assessment*.

Goldberg, D. P. (1978). General Health Questionnaire-12. *Australian Journal of Psychology*.

Haghighi, M., Oladbaniadam, K., Mohaddesi, H., & Rasuli, J. (2022). Individual counseling in mothers bereaved by pregnancy loss: A randomized clinical trial. *J Educ Health Promot, 11*, 209. doi:10.4103/jehp.jehp_1765_21

Hamilton, M. (1960). A rating scale for depression. *Journal of neurology, neurosurgery, and psychiatry, 23*(1), 56.

Huberty, J., Sullivan, M., Green, J., Kurka, J., Leiferman, J., Gold, K., & Cacciatore, J. (2020). Online yoga to reduce post traumatic stress in women who have experienced stillbirth: a randomized control feasibility trial. *BMC Complement Med Ther, 20*(1), 173. doi:10.1186/s12906-020-02926-3

Hung, H.-M., Kuo, P.-L., Lee, C. S., & Chen, C.-H. (2023). Effectiveness of mental health website intervention on stress and depression for women with recurrent miscarriage: A randomized controlled trial. *Health Care for Women International, 44*(4), 496-508. doi:10.1080/07399332.2022.2141744

Jensen, K. H. K., Krog, M. C., Koert, E., Hedegaard, S., Chonovitsch, M., Schmidt, L., . . . Nielsen, H. S. (2021). Meditation and mindfulness reduce perceived stress in women with recurrent pregnancy loss: a randomized controlled trial. *Reprod Biomed Online, 43*(2), 246-256. doi:10.1016/j.rbmo.2021.04.018

Johnson, J. E., Price, A. B., Kao, J. C., Fernandes, K., Stout, R., Gobin, R. L., & Zlotnick, C. (2016). Interpersonal psychotherapy (IPT) for major depression following perinatal loss: a pilot randomized controlled trial. *Arch Womens Ment Health, 19*(5), 845-859. doi:10.1007/s00737-016-0625-5

Johnson, O. (2009). The impact of bereavement intervention on levels of grief in pregnant women who experience pre-twenty-week loss (Order No. 3400972). Available from GenderWatch; ProQuest Dissertations & Theses A&I; ProQuest Dissertations & Theses Global: The Humanities and Social Sciences Collection; ProQuest Dissertations & Theses Global A&I: The Sciences and Engineering Collection. (305138960). Retrieved from http://easyaccess.lib.cuhk.edu.hk/login?url=https://www.proquest.com/dissertations-theses/impact-bereavement-intervention-on-levels-grief/docview/305138960/se-2.

Johnson, O. P., & Langford, R. W. (2015). A randomized trial of a bereavement intervention for pregnancy loss. *Journal of Obstetric, Gynecologic & Neonatal Nursing, 44*(4), 492-499.

Kersting, A., Dölemeyer, R., Steinig, J., Walter, F., Kroker, K., Baust, K., & Wagner, B. (2013). Brief Internet-based intervention reduces posttraumatic stress and prolonged grief in parents after the loss of a child during pregnancy: a randomized controlled trial. *Psychother Psychosom, 82*(6), 372-381. doi:10.1159/000348713

Kersting, A., Kroker, K., Schlicht, S., Baust, K., & Wagner, B. (2011). Efficacy of cognitive behavioral internet-based therapy in parents after the loss of a child during pregnancy: pilot data from a randomized controlled trial. *Archives of Women's Mental Health, 14*(6), 465-477. doi:10.1007/s00737-011-0240-4

Kong, G. W., Chung, T. K., & Lok, I. H. (2014). The impact of supportive counselling on women's psychological wellbeing after miscarriage--a randomised controlled trial. *Bjog, 121*(10), 1253-1262. doi:10.1111/1471-0528.12908

Kroenke, K., Spitzer, R. L., & Williams, J. B. W. (2001). The PHQ-9. *Journal of General Internal Medicine, 16*(9), 606-613. doi:10.1046/j.1525-1497.2001.016009606.x

Lovibond, P. F., & Lovibond, S. H. (1995). The structure of negative emotional states: Comparison of the Depression Anxiety Stress Scales (DASS) with the Beck Depression and Anxiety Inventories. *Behav Res Ther, 33*(3), 335-343. doi:https://doi.org/10.1016/0005-7967(94)00075-U

Mecdi Kaydirak, M., & Aslan, E. (2021). Efficacy of Nursing Support in the Pre- and Postmedical Termination of Pregnancy Phases: A Randomized Study. *Omega (Westport), 84*(1), 51-68. doi:10.1177/0030222819877791

Nasrollahi, M., Ghazanfar Pour, M., Ahmadi, A., Mirzaee, M., & Alidousti, K. (2022). Effectiveness of mindfulness-based stress reduction on depression, anxiety, and stress of women with the early loss of pregnancy in southeast Iran: a randomized control trial. *Reprod Health, 19*(1), 233. doi:10.1186/s12978-022-01543-2

Navidian, A., & Saravani, Z. (2018). Impact of cognitive behavioral-based counseling on grief symptoms severity in mothers after stillbirth. *Iranian Journal of Psychiatry and Behavioral Sciences, 12*(1).

Navidian, A., Saravani, Z., & Shakiba, M. (2017). Impact of psychological grief counseling on the severity of post-traumatic stress symptoms in mothers after stillbirths. *Issues in mental health nursing, 38*(8), 650-654.

Newham, J. J., Westwood, M., Aplin, J. D., & Wittkowski, A. (2012). State–trait anxiety inventory (STAI) scores during pregnancy following intervention with complementary therapies. *Journal of affective disorders, 142*(1-3), 22-30.

Nikcevic, A., Snijders, R., Nicolaides, K., & Kupek, E. (1999). Some psychometric properties of the Texas Grief Inventory adjusted for miscarriage. *British Journal of Medical Psychology, 72*(2), 171-178.

Prigerson, H. G., Maciejewski, P. K., Reynolds III, C. F., Bierhals, A. J., Newsom, J. T., Fasiczka, A., . . . Miller, M. (1995). Inventory of Complicated Grief: a scale to measure maladaptive symptoms of loss. *Psychiatry research, 59*(1-2), 65-79.

Radloff, L. S. (1977). The CES-D Scale: A self-report depression scale for research in the general population [Press release]

Ritsher, J. B., & Neugebauer, R. (2002). Perinatal Bereavement Grief Scale: distinguishing grief from depression following miscarriage. *Assessment, 9*(1), 31-40.

Rocha, J., Nunes, C., Leonardo, A., Correia, M. J., Fernandes, M., Paúl, M. C., & Almeida, V. (2018). Women generating narratives after an unwanted prenatal diagnosis result: randomized controlled trial. *Arch Womens Ment Health, 21*(4), 453-459. doi:10.1007/s00737-018-0822-5

Rosenbaum, J. L., Smith, J. R., Yan, Y., Abram, N., & Jeffe, D. B. (2015). Impact of a neonatal-bereavement-support DVD on parental grief: A randomized controlled trial. *Death studies, 39*(4), 191-200.

Sahın, N., & Durak, A. (1995). A brief coping styles inventory for university-students. Turk Psikoloji Dergisi, 10(34).

Séjourné, N., Callahan, S., & Chabrol, H. (2010). The utility of a psychological intervention for coping with spontaneous abortion. *Journal of Reproductive and Infant Psychology, 28*(3), 287-296. doi:10.1080/02646830903487334

Sherbourne, C. D., & Stewart, A. L. (1991). The MOS social support survey. *Social Science & Medicine, 32*(6), 705-714.

Simpson, C., Lee, P., & Lionel, J. (2015). The effect of bereavement counseling on women with psychological problems associated with late pregnancy loss. *Journal of Asian Midwives (JAM), 2*(2), 5-20.

Spielberger, C. D., Gonzalez-Reigosa, F., Martinez-Urrutia, A., Natalicio, L. F., & Natalicio, D. S. (1971). The state-trait anxiety inventory. *Revista Interamericana de Psicologia/Interamerican journal of psychology, 5*(3 & 4).

Sun, S., Li, J., Ma, Y., Bu, H., Luo, Q., & Yu, X. (2018). Effects of a family-support programme for pregnant women with foetal abnormalities requiring pregnancy termination: A randomized controlled trial in China. *Int J Nurs Pract, 24*(1). doi:10.1111/ijn.12614

Swanson, K. M., Chen, H.-T., Graham, J. C., Wojnar, D. M., & Petras, A. (2009). Resolution of depression and grief during the first year after miscarriage: a randomized controlled clinical trial of couples-focused interventions. *Journal of Women's Health, 18*(8), 1245-1257.

Toedter, L. J., Lasker, J. N., & Alhadeff, J. M. (1988). The Perinatal Grief Scale: development and initial validation. *American Journal of Orthopsychiatry, 58*(3), 435.

Wang, N., Wang, M., Huang, J., Allen, J., Elder, E., Fu, L., . . . Gamble, J. (2023). Effects of the STress-And-coping suppoRT (START) intervention on depression and coping of Chinese women seeking a first-trimester abortion: A randomized controlled trial. *J Affect Disord, 324*, 121-128. doi:10.1016/j.jad.2022.12.086

Wewers, M. E., & Lowe, N. K. (1990). A critical review of visual analogue scales in the measurement of clinical phenomena. *Research in Nursing & Health, 13*(4), 227-236.

Zigmond, A. S., & Snaith, R. P. J. A. p. s. (1983). The hospital anxiety and depression scale. *67*(6), 361-370.

Zung, W. W. (1971). A rating instrument for anxiety disorders. *Psychosomatics, 12*(6), 371-379. doi:10.1016/s0033-3182(71)71479-0
